# Tetramethylguanidine-functionalized melamine as a multifunctional organocatalyst for the expeditious synthesis of 1,2,4-triazoloquinazolinones

**DOI:** 10.1038/s41598-021-91463-1

**Published:** 2021-07-14

**Authors:** Mahnoush Keshavarz, Mohammad G. Dekamin, Manouchehr Mamaghani, Mohammad Nikpassand

**Affiliations:** 1 Department of Chemistry, Faculty of Basic Sciences, Rasht Branch , Islamic Azad University, P.O. Box 41335-3516, Rasht, Iran; 2 Department of Chemistry, Iran University of Science and Technology, 16846-13114 Tehran, Iran; 3 Department of Chemistry, Faculty of Sciences, University of Guilan, P.O. Box 41335-1914, Rasht, Iran

**Keywords:** Environmental chemistry, Catalysis, Green chemistry, Materials chemistry, Medicinal chemistry, Organic chemistry, Supramolecular chemistry

## Abstract

Novel nano-ordered 1,1,3,3-tetramethylguanidine-functionalized melamine (Melamine@TMG) organocatalyst was prepared and adequately identified by various techniques including FTIR, EDX, XRD and SEM spectroscopic or microscopic methods as well as TGA and DTG analytical methods. The Melamine@TMG, as an effective multifunctional organocatalyst, was found to promote smoothly the three-component synthesis of 1,2,4-triazoloquinazolinone derivatives using cyclic dimedone, 3-amino-1,2,4-triazole and different benzaldehyde derivatives in EtOH at 40 °C. This practical method afforded the desired products in high to excellent yields (86–99%) and short reaction times (10–25 min). The main advantages of this new method are the use of heterogeneous multifunctional nanocatalyst, simple work-up procedure with no need for chromatographic purification, highly selective conversion of substrates and recyclability of the catalyst, which could be used in five consecutive runs with only a small decrease in its activity.

## Introduction

The discovery of efficient catalytic systems for synthetic chemical reactions is a milestone and key principle in green and sustainable chemistry, which has emerged as an active area of research. Organocatalysis is one of the most popular and rapidly growing research fields since its fundamental “renaissance” at the beginning of the twenty-first century^1–12^. Organocatalytic systems are generally homogenous in nature^2,3,11–13^. However, when they are changed into nano-ordered systems it has been proven to possess heterogeneous catalytic behaviour. This may lead to the designing and exploration of new carbonaceous materials which itself is a hot topic in both academia and industry^14–21^. In addition, a high surface to volume ratio is leading to high activity, selectivity, and often more stability. Nano-ordered organocatalysts can reduce the temperature and the amount of hazardous waste in chemical transformations, and by enhancing the selectivity of a reaction avoid undesired products as well as be simply recovered from the crude products and reused in multiple reactions. Therefore, heterogeneous organocatalysts have resulted in higher yields and cleaner synthesis^22–33^. In this regard, 1,3,5-triazine-2,4,6-triamine is an appropriate choice for designing new heterogeneous organocatalysts by anchoring proper organic molecules to its surface in order to produce multifunctional catalytic systems. 1,3,5-triazine-2,4,6-triamine, namely melamine or cyanuramide, is symmetric heterocyclic base and has been widely used in different industries such as plastics, resins, fertilizers, and insecticides^34^. Furthermore, the various catalysts consist of functionalized melamine have been reported for the synthesis of heterocyclic scaffolds having nitrogen, oxygen, or sulfur in their structures. In fact, melamine-based catalytic systems promote organic transformations smoothly, and hence are more environment friendly and cost-effective^19,35–43^. On the other hand, guanidine and its derivatives have been used, as homogeneous bifunctional organocatalysts, in different organic transformations in recent years^4,5,44–50^. Therefore, designing and exploration of new heterogeneous organocatalytic systems based on melamine and guanidine derivatives would be very desirable.

Quinazolinone derivatives have received considerable attention as a well-known family of *N*-containing heterocyclic scaffolds in medicinal chemistry. They possess significant biological and medicinal properties such as antimicrobial^51,52^, anticonvulsant^53^, antihistaminic^54^, antihypertensive^55^, etc. Among various reported procedures for the synthesis of 1,2,4-triazoloquinazolinone derivatives, the three-component reaction of enolizable 1,3-dicarbonyls, 3-amino-1,2,4-triazole, and aldehydes/ketones has been known as the most beneficial method in terms of obtained yield, selectivity and green chemistry principles^51,56–66^. Some recent examples include DABCO based ionic liquid^67^, TiO_2_ nanoparticles supported ionic liquids^68^, magnetic nanoparticles coated by silica with bis dicationic bridge^69^, sulfonic acid functionalized SBA-15^70^, nanoporous silica^71^, H_4_[W_12_SiO_40_] grafted on magnetic chitosan^72^, l-proline^51^, *p*-toluenesulfonic acid monohydrate^73^, sulfamic acid^74^, anthranilic acid^75^, molecular iodine^76^, etc^77,78^. However, there is a lot more room to explore new and more efficient catalytic systems for this MCR which can work under green conditions using organocatalytic systems.

**Scheme 1:**
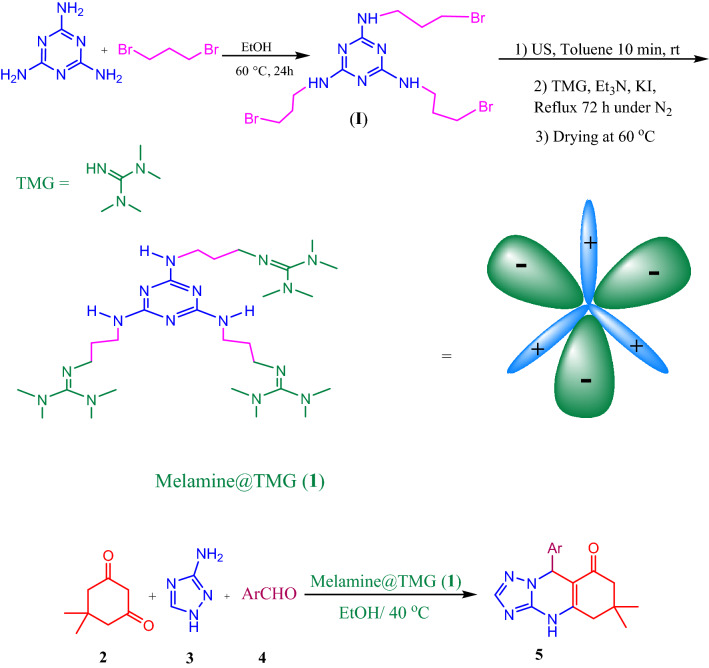
Preparation of nano-ordered multifunctional Melamine@TMG organocatalyst and its application for the three-component synthesis of 1,2,4-triazoloquinazolinones **5a–j**.

In this present work, we hereby report a green approach for the synthesis of 1,2,4-triazoloquinazolinones catalyzed by the 1,1,3,3-tetramethylguanidine superbase^79^ anchored onto melamine (Melamine@TMG, **1**) as a novel nano-ordered multifunctional and heterogeneous organocatalyst (Scheme 1). The Melamine@TMG catalyst was prepared in two definite steps: The melamine surface modification with 3-bromopropyl groups was the first step to afford Melamine@PrBr intermediate (**I**) ^41^; the second step was functionalization of the obtained Melamine@PrBr nanoparticles with the TMG base through bimolecular nucleophilic substitution (S_N_2). The obtained nanocatalyst was characterized by different spectroscopic, microscopic, and analytical techniques. The efficiency of the Melamine@TMG nanocatalyst was examined in the synthesis of 1,2,4-triazoloquinazolinones. After completion of reactions, the Melamine@TMG nanocatalyst was separated from the crude products by washing in EtOH during crystallization of the desired products and exhibited the best catalytic performance in the first cycle (98% isolated product) and it’s activity decreased slightly after four times of recycling (84% isolated product).

## Experimental

### Materials and instruments

All chemicals, reagents, and solvents were supplied by Merck, Sigma-Aldrich, or local Companies and used as received, except for benzaldehyde which a fresh-distilled sample was used. X-ray diffraction (XRD) patterns were obtained using an X’Pert PRO MPD PANalytical Company. The infrared spectra of the catalyst and products were measured by a Bruker-Vector 33 Fourier transform infrared spectrometer (FTIR) using KBr discs. Scanning electron microscopy (SEM) and energy-dispersive X-ray (EDX) analysis were carried out on a TESCAN-Mira III. The thermogravimetric analysis (TGA) and derivative thermogravimetric (DTG) curves were determined on a TGA/DSC Mettler Toledo apparatus. ^1^H and ^13^C nuclear magnetic resonance (NMR) spectra were recorded on a Bruker AVANCE 500 in DMSO-*d*_6_ at ambient temperature. The CHN analysis were measured by a Thermo Scientific Eager 800. All compounds, except **5c** and **5i**, are known and their structures were confirmed by comparison of their melting points as well as FTIR and ^1^H NMR spectral data with the authentic samples (Supplementary Information).

### Preparation of the Melamine@TMG catalyst (1)

#### Preparation of ***N***^2^,***N***^4^,***N***^6^-tris(3-bromopropyl)-1,3,5-triazine-2,4,6-triamine (Melamine@PrBr, **I**)

Melamine (10 mmol, 1.261 g) was dispersed in EtOH (150 mL) by sonication for 10 min. Then, the obtained mixture was transferred into a round bottom flask and 1,3-dibromopropane (29.4 mmol, d = 1.98 g.cm^-3^, 3.0 mL) was added^41^. The obtained colloidal mixture was heated at 60 °C under stirring by a mechanical stirrer (400 rpm) for 24 h. Afterward, the reaction mixture was filtered off and the white solid was washed twice with hot EtOH and dried under vacuum to afford Melamine@PrBr (**I**) in 96% yield.

#### Preparation of 1,1,3,3-tetramethylguanidine-functionalized melamine nanocatalyst (Melamine@TMG, **1**)

The as-prepared Melamine@PrBr (1.2 g) was dispersed in dry toluene (150 mL) in a round-bottom flask by ultrasonication for 10 min. Then, 1,1,3,3-tetramethylguanidine (TMG, 39.9 mmol, d = 0.918 g.cm^-3^, 5.0 mL) in triethylamine (79 mmol, d = 0.73 g.cm^-3^, 11 mL) and KI (1.2 mmol, 0.02 g) were added to the obtained mixture under mechanical stirring (400 rpm). The reaction mixture was then heated under N_2_ atmosphere for 72 h and the final product was filtered off and washed twice with EtOH and dried at 60 °C under vacuum for 2 h to afford the Melamine@TMG, as a white solid, in 91% yield.

### General procedure for the synthesis of 1,2,4-triazoloquinazolinones 5a–j catalyzed by Melamine@TMG (1)

To a mixture of dimedone (**2**, 1 mmol, 0.140 g), 3-amino-1,2,4-triazole (**3**, 1 mmol, 0.084 g) and arylaldehyde (**4**, 1 mmol) in EtOH (3 mL), Melamine@TMG (**1**, 2.5 mol%, 15 mg) was added. The reaction mixture was stirred at 40 °C for times mentioned in Table 2 and the progress of the reaction was observed by TLC. After completion of the reaction, a precipitate was formed which was dissolved in additional EtOH (3 mL) by heating and filtered off to separate the catalyst **1**. The filtrate was allowed to stand at room temperature to give pure products **5a–j**. The separated catalyst was then washed with EtOAc and *n*-hexane (2 mL) for 10 min., respectively, and finally dried at 60 °C under vacuum for 0.5 h to be used for further next runs.

## Results and discussion

### Characterization of the nano-ordered Melamine@TMG organocatalyst (1)

The as-prepared nano-ordered multifunctional and heterogeneous Melamine@TMG organocatalyst (**1**) was properly characterized by Fourier transform infrared (FTIR) and energy dispersive X-Ray (EDX) spectroscopy, X-ray diffraction (XRD), scanning electron microscopy (SEM), and thermal gravimetric analysis (TGA) and derivative thermogravimetry (DTG) techinques before investigation of its catalytic activity in the synthesis of 1,2,4-triazoloquinazolinones .

#### FTIR spectra of Melamine@TMG organocatalyst (**1**)

**Figure 1:**
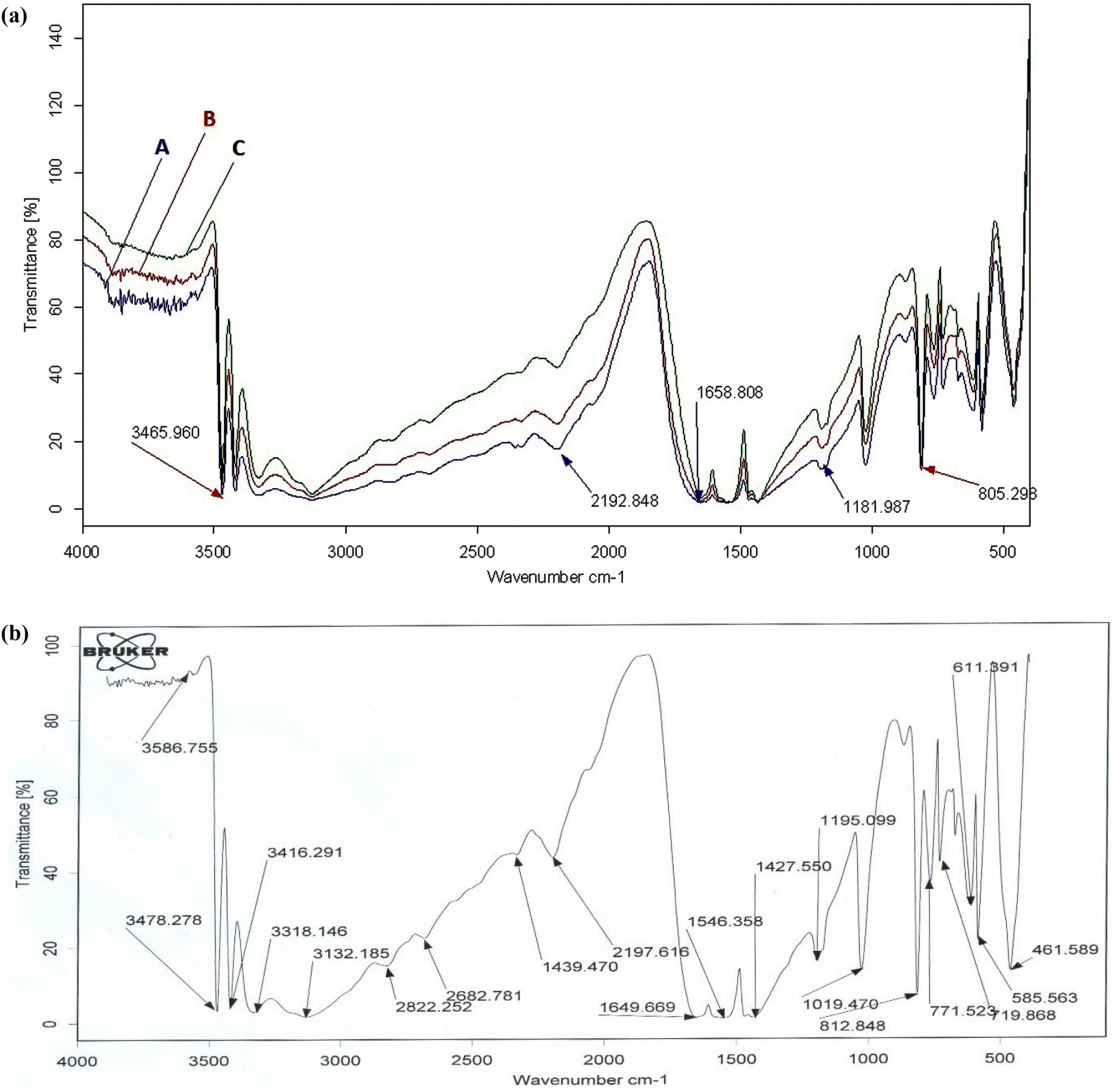
FTIR spectra of melamine (**A**), the Melamine@PrBr (**B**), and the nano-ordered Melamine@TMG (**1**, **C**, part a) and the recycled Melamine@TMG catalyst after five consecutive runs in the model reaction (part b).

In the FTIR spectrum of melamine (Fig. 1a, A), the bands at 3483 and 3411 cm^−1^ are related to the asymmetric and symmetric stretching vibrations of the N–H bonds of melamine^42,80,^. The band at 1673 cm^−1^ is attributed to the bending vibrations of the NH_2_ and the band at 1525 cm^−1^ is related to the stretching vibrations of NCN of melamine^42,81^. For the Melamine@PrBr (Fig. 1a, B) and Melamine@TMG (Fig. 1a, C), new signals appeared at 1644, 1540, and 1014 cm^−1^, which are associated with the stretching and scissoring vibrations of C=N, N–H, and C–N bonds of both TMG and melamine, respectively. Appearance of these characteristic bands confirm the existence of TMG moiety in the structure of the catalyst 1^79^. By considering this point that there are similar bands in the structure of melamine and TMG, it is not possible to determine clearly the signals of these two structures by FTIR analysis. Furthermore, the FTIR spectra of Melamine@TMG after five consecutive runs in the model reaction (Fig. 1b) demonstrated very good similarity with the fresh catalyst **1**. This finding shows that the structure of Melamine@TMG organocatalyst (**1**) does not change during five consecutive runs.

#### XRD patterns of the Melamine@TMG organocatalyst (**1**)

**Figure 2:**
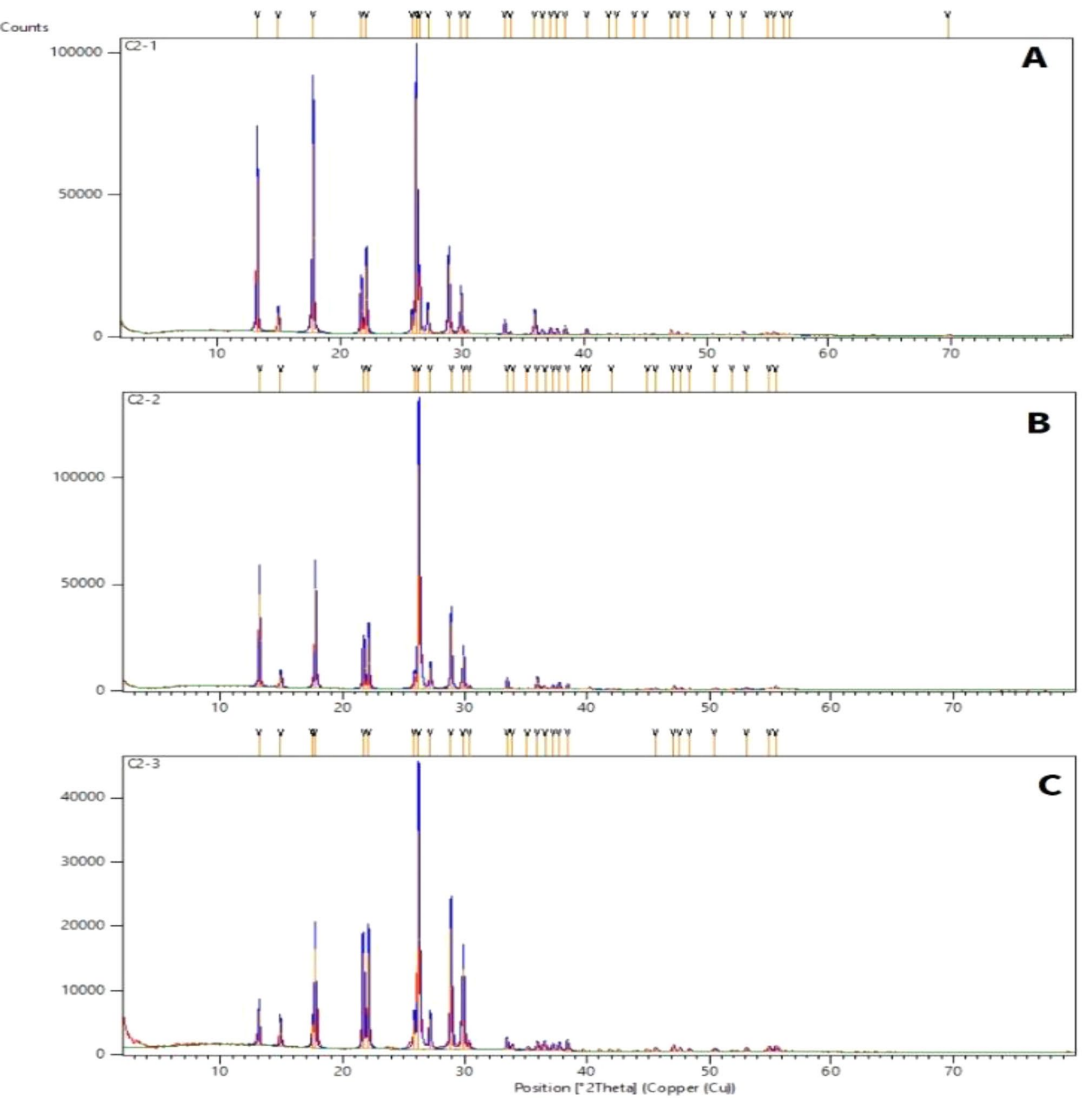
XRD patterns of the melamine (**A**), the Melamine@PrBr (**B**), and the nano-ordered Melamine@TMG (**1**, **C**).

Comparative XRD patterns of the Melamine@TMG organocatalyst (**1**) and its components are presented in Fig. 2. As data in Fig. 2a show, XRD pattern of the melamine has a non-amorphous and crystalline phase (2θ: 13.22, 17.78, 21.74, 22.14, 26.28, 26.51, 28.90, 29.89 and 35.91^o^). Furthermore, the XRD patterns of both Melamine@prBr (Fig. 2b, 2θ: 13.20, 17.77, 21.73, 22.13, 26.25, 28.89 and 29.88^o^) and Melamine@TMG organocatalyst (**1**, Fig. 2c, 2θ: 17.74, 21.70, 22.12, 26.24, 28.88, and 29.86^o^﻿) show almost the same peaks compared to the melamine. Although the non-amorphous structure of the melamine has not been completely changed, but the intensity of different peaks has been altered^80^.

#### Energy dispersive X-ray spectroscopy of the nano-ordered Melamine@TMG (**1**)

**Figure 3:**
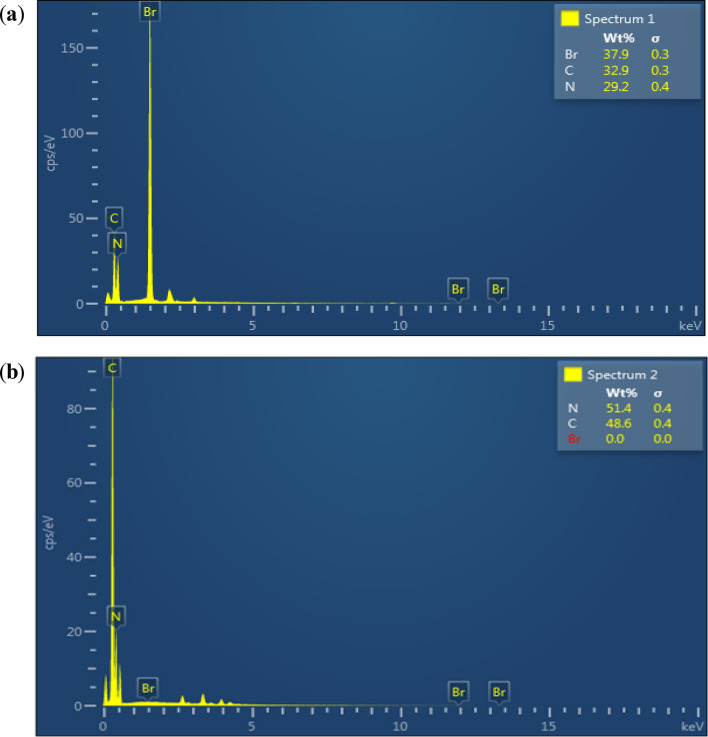
The EDX spectra of the Melamine@PrBr (**a**) and nano-ordered Melamine@TMG (**1**, **b**).

The chemical composition of the Melamine@PrBr (**I**) and Melamine@TMG organocatalyst (**1**) was determined by energy-dispersive X-ray (EDX) spectroscopy (Fig. 3). The EDX spectra of the Melamine@PrBr and nano-ordered Melamine@TMG showed the expected elements including Br, N and C. The obtained results confirm successful anchoring of 1,3-dibromopropane, as the linker, and TMG, as an organic base, to the melamine during depicted steps for preparation of the Melamine@TMG catalyst (**1**) in Scheme 1.

#### Scanning electron microscopy images of the Melamine@TMG organocatalyst (**1**)

**Figure 4:**
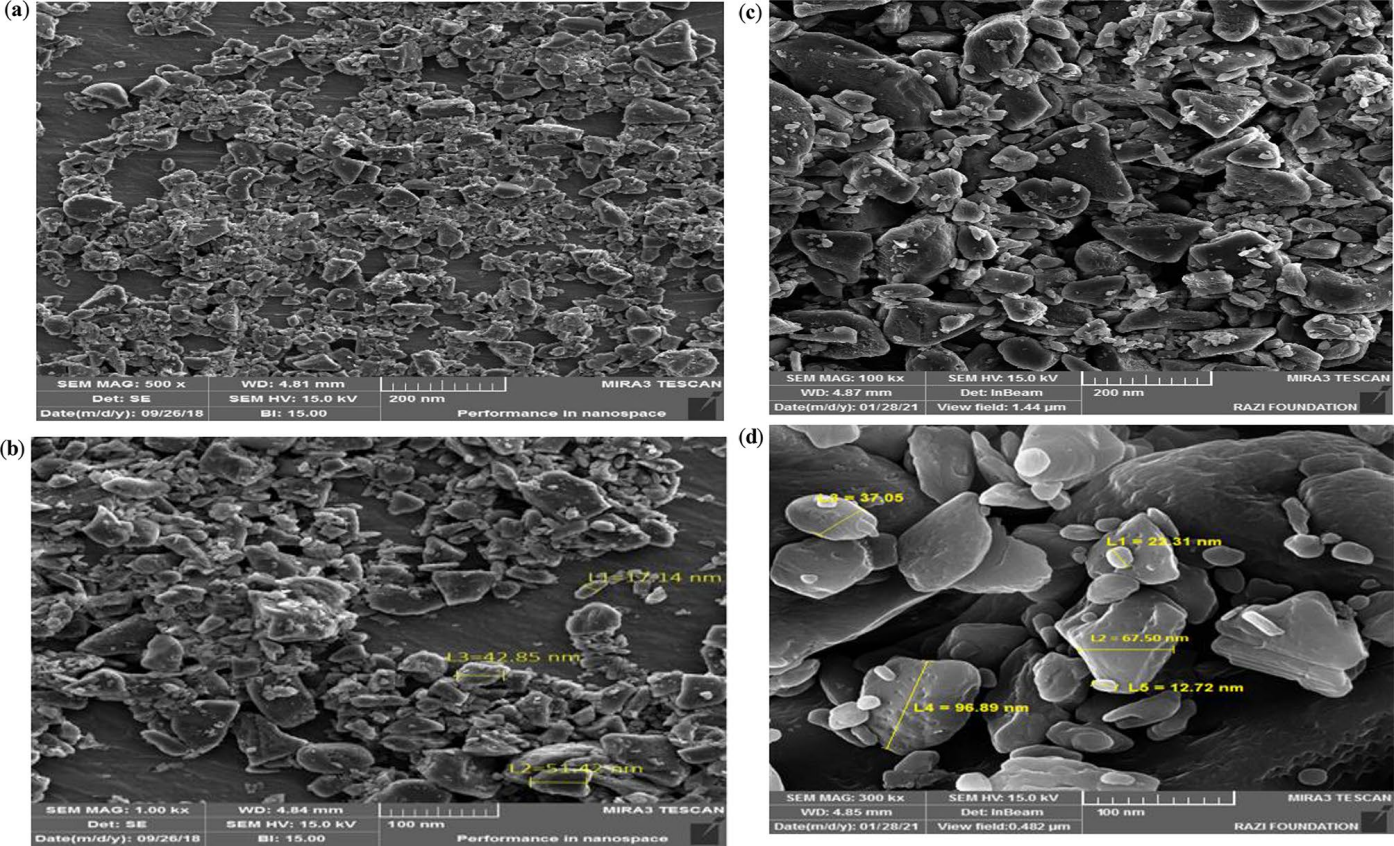
SEM images of the fresh nano-ordered Melamine@TMG (**1**, **a**,**b**) and the recycled Melamine@TMG catalyst after five consecutive runs in the model reaction (**c**,**d**).

The scanning electron microscopy (SEM) images demonstrated that the nano-ordered Melamine@TMG (Fig. 4a,b) has a layered structure and the size of nanoparticles are mainly between 17 and 52 nm. On the other hand, the morphology of Melamine@TMG catalyst after five cycles in the model reaction was also preserved considerably (Fig. 4c,d).

#### Thermal gravimetric analysis and derivative thermogravimetry of the Melamine@TMG (**1**)

**Figure 5:**
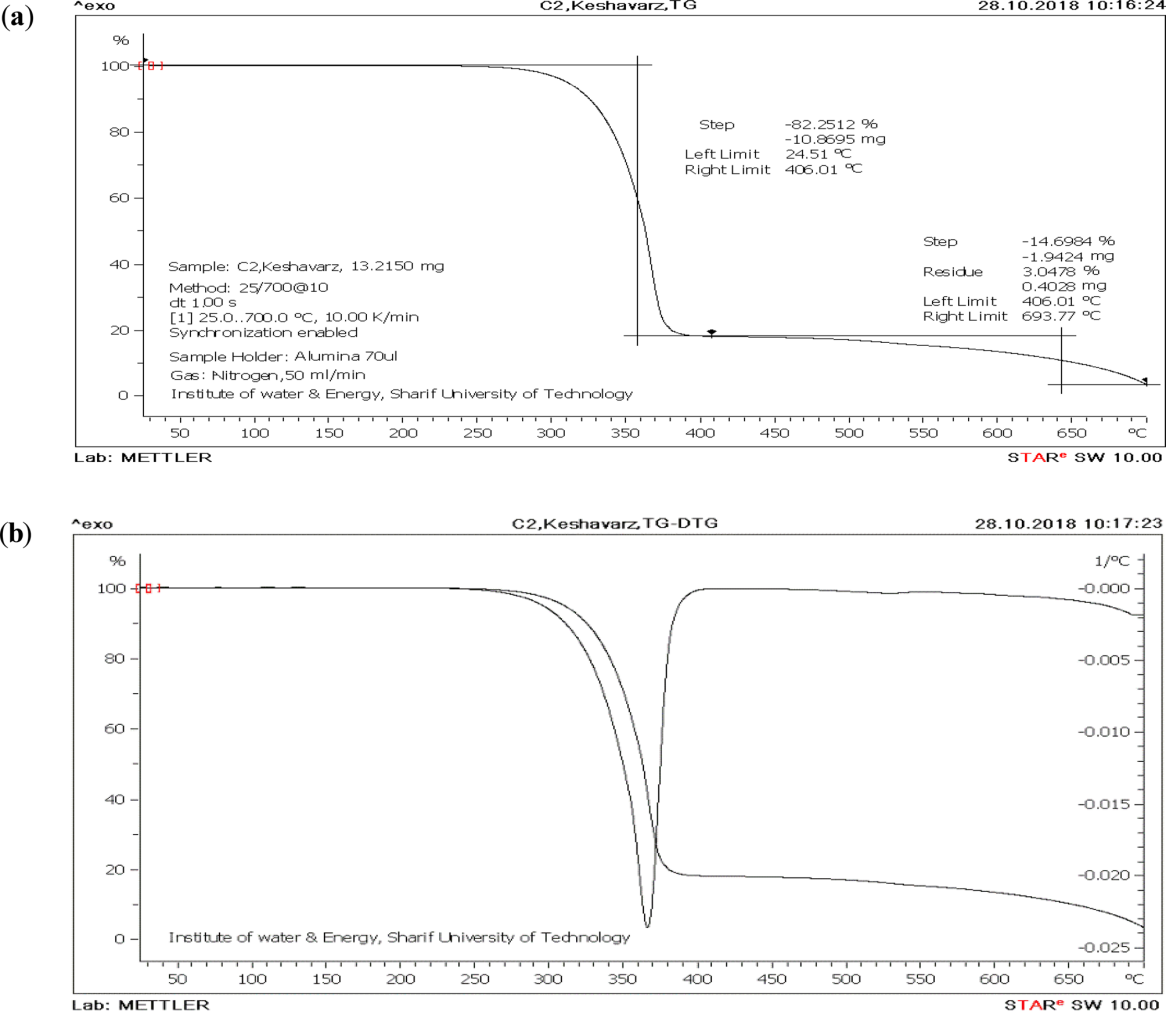
TGA (**a**) and TG-DTG curves of the Melamine@TMG organocatalyst (**1**, **b**).

The thermogravimetric analysis (TGA) of the Melamine@TMG organocatalyst (**1**) shows a two-step mass loss of the organic materials between 250–700 °C (Fig. 5a). The analysis represents that two distinct weight loss stages occur during the pyrolysis of the nanocatalyst. According to data presented in Fig. 5a, the residual mass percent of the Melamine@TMG at 700 °C is about 3.0%. Therefore, the weight loss of 82% from 250 to 406 °C can be attributed mostly to the loss and decomposition of organic TMG, its propylene linkage, and melamine’s condensation on heating with the elimination of ammonia to form "melam", "melem", "melon"^82^, while the second weight loss peaks of 14.69% in the range of 406–694 °C can be assigned to complete decomposition of the melamine residue. These findings are also confirmed by the values reported in the derivative thermogravimetry (DTG) analysis (Fig. 5b). The most weight loss occurs at about 370 °C, which is related to the first stage of thermal decomposition. Although this catalyst decomposes mostly at 370 °C, it can be noted that the Melamine@TMG catalyst (**1**) demonstrated thermal stability up to 250 °C. Hence, it can easily be used in the range of room temperature to 250 °C without any significant change in its structure and catalytic activity.

### Exploration of the catalytic activity of nano-ordered Melamine@TMG organocatalyst (1)

The catalytic activity of the as-prepared nano-ordered Melamine@TMG (**1**) was investigated for the three-component synthesis of 1,2,4-triazoloquinazolinones (Scheme 1). Initially, the three-component reaction of benzaldehyde (**4a**), 3-amino-1,2,4-triazole (**3**), and dimedone (**2**) was carried out in various solvents and the absence/presence of different catalysts to find the optimized conditions for the model reaction. The obtained results are reported in Table 1. The results revealed that the model reaction in the absence of any catalyst or ordinary acidic and basic catalysts including CH_3_CO_2_H and NaOH proceeds to provide the desired product, 6,6-dimethyl-9-phenyl-5,6,7,9-tetrahydro-[1, 2, 4] triazolo[5,1-*b*]quinazolin-8(4*H*)-one (**5a**) in longer reaction times and lower yields (Entries 1–5). Indeed, the best results were obtained by using the novel Melamine@TMG nanocatalyst (**1**) in EtOH, as a green solvent, at 40 °C (Entries 6–9). The amount of the nanocatalyst loading was also screened (Entries 6–12). It is obvious from the obtained results that by employing 2.5 mol% (15 mg) nanocatalyst loading in EtOH at 40 °C, the reaction affords the desired 1,2,4-triazoloquinazolinone **5a** in lower reaction time and excellent yield (> 98%, Entry 8).

**Table 1:** Optimization of the conditions for the model reaction.
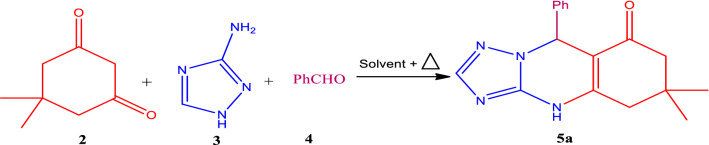

Entry	Catalyst (mol%)	Solvent	Temp. (°C)	Time (min)	Yield (%)
1	–	EtOH	Reflux	120	45
2	–	H_2_O	Reflux	120	34
3	–	CH_3_CN	Reflux	120	42
4	CH_3_CO_2_H	CH_3_CO_2_H	60	30	76
5	NaOH (1 mmol)	EtOH	Reflux	30	71
6	Melamine—TMG (15 mol%)	EtOH	40	20	83
7	Melamine—TMG (5 mol%)	EtOH	40	20	88
8	Melamine—TMG (2.5 mol%)	EtOH	40	10	98
9	Melamine—TMG (2.5 mol%)	EtOH	r.t	25	92
10	Melamine—TMG (2.5 mol%)	EtOH	Reflux	5	99
11	Melamine—TMG (2.5 mol%)	H_2_O	40	25	87
12	Melamine—TMG (2.5 mol%)	H_2_O	Reflux	15	90

Reaction conditions: dimedone (**2**, 1 mmol), 3-amino-1,2,4-triazole (**3**, 1 mmol), benzaldehyde (**4a**, 1 mmol), catalyst (if not otherwise specified) and solvent (2 mL).

In the next step, other carbocyclic aromatic aldehydes **5b–j** with different substituents were used to develop the scope of the studied three-component reaction catalyzed by the multifunctional Melamine@TMG organocatalyst (**1**). The obtained results have been summarized in Table 2. As data in Table 2 show, all the studied aldehydes with both electron-withdrawing and electron-donating substituents were involved in the optimized conditions smoothly to produce the corresponding products in high to excellent yields. In general, the kind and position of functional groups on the aromatic ring of aldehyde exhibits an obvious impact on the required time for completion of the reaction. Indeed, aromatic aldehydes bearing electron-donating substituents (Entries 2–4 and 8) required longer reaction times compared to those ones containing electron-withdrawing groups (Entries 5-7 and 9,10).

**Table 2:** Synthesis of various derivatives of 1,2,4-triazoloquinazolinones **5a–j** catalyzed by the nano-ordered Melamine@TMG organocatalyst (**1**).
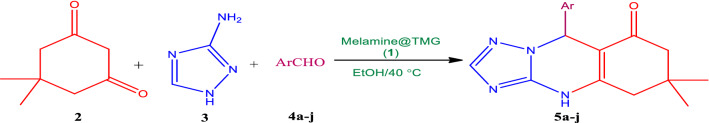

Entry	Aldehyde 4	Product 5	Time (min)	Yield^a^ (%)	Mp (°C)		
					Found	Reported	References
1	PhCHO	**5a**	10	98	250–252	248–250	^74,77,78^
2	2-MeOC_6_H_4_CHO	**5b**	20	90	242–244	240–243	^76,83^
3	3,4-(MeO)_2_C_6_H_3_CHO	**5c**	25	89	225–227	–	This work
4	4-MeC_6_H_4_CHO	**5d**	20	86	262–264	264–269	^74,78^
5	4-ClC_6_H_4_CHO	**5e**	8	98	303–305	303–305	^74,78^
6	3-O_2_NC_6_H_4_CHO	**5f.**	5	97	267–269	266–269	^74,78^
7	4-O_2_NC_6_H_4_CHO	**5 g**	5	98	294–297	290–294	^84^
8	4-HOC_6_H_4_CHO	**5 h**	20	91	305–308	> 300	^74,78^
9	3,4-(Cl)_2_C_6_H_3_CHO	**5i**	5	99	324–326	–	This work
10	4-FC_6_H_4_CHO	**5j**	5	99	257–259	258–260	^85^

Reaction conditions: dimedone (**2**, 1 mmol), 3-amino-1,2,4-triazole (**3**, 1 mmol), aldehyde (**4a–j**, 1 mmol), Melamine@TMG nanocatalyst (**1**, 2.5 mol%, 15 mg) in EtOH (2 mL) at 40 °C.

^b^Isolated yield.

**Scheme 2:**
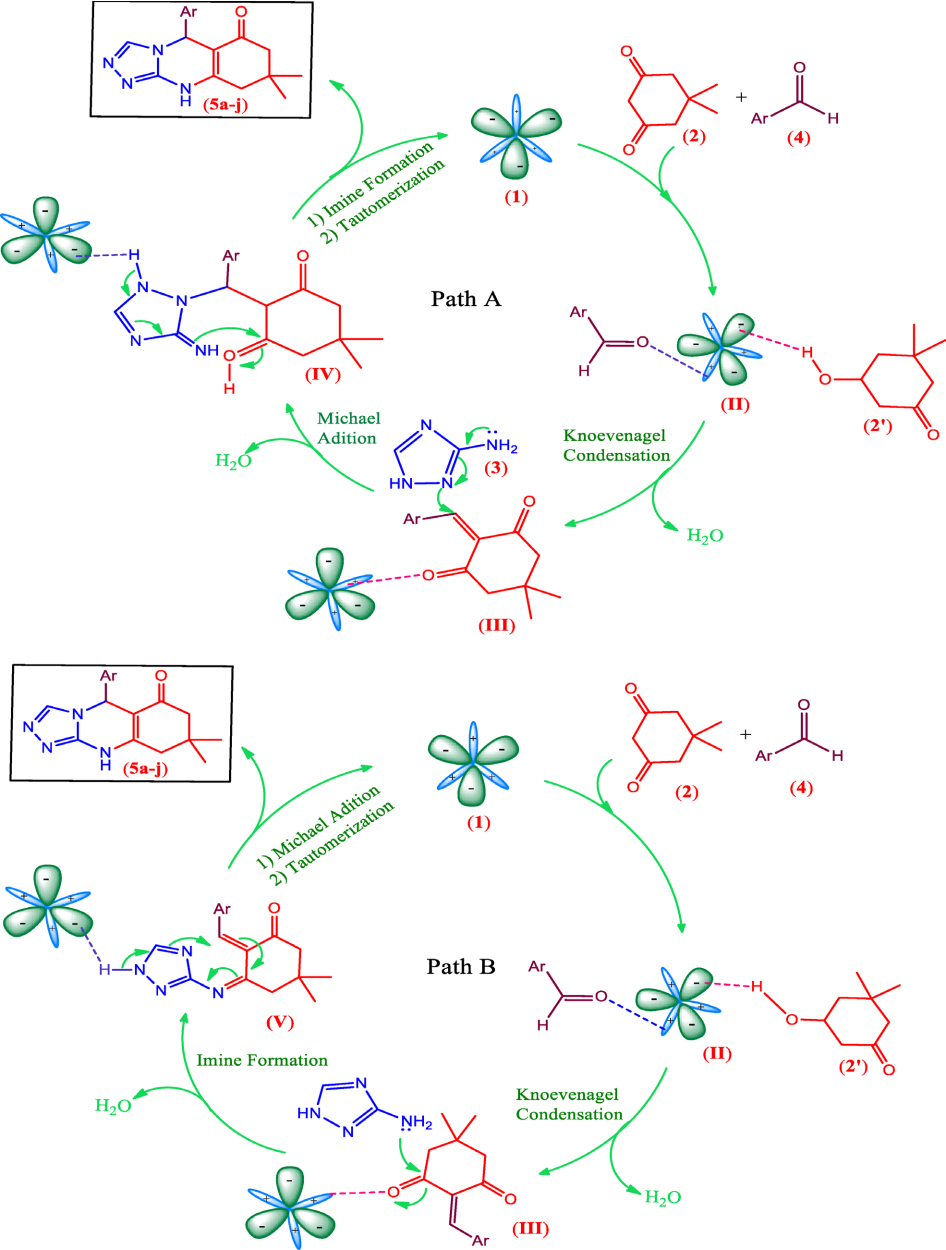
Proposed mechanism for the synthesis of 1,2,4-triazoloquinazolinones **5a–j** catalyzed by the nano-ordered Melamine@TMG organocatalyst (**1**).

According to the functional groups existing in the structure of catalyst **1** and reactivity of different aldehydes **4a–j**, a reasonable mechanism for the synthesis of 1,2,4-tria-zoloquinazolinones **5a–j** catalyzed by the multifunctional Melamine@TMG organocatalyst (**1**) is presented in Scheme 2. In the presence of the melamine-TMG nanocatalyst, the dimedone component **2** equilibrates with its corresponding enol form **2′** and reacts with the activated aldehydes **4** to form the intermediate **III** through the Knoevenagel condensation. After formation of the intermediate **III**, there are two possible paths for the reaction: Intermediate **III** at first takes part in the Michael addition with 3-amino-1,2,4-triazole **3** to form the intermediate **IV** and subsequent cyclization by imine formation. Then, the desired products **5a–j** are formed after tautomerization of the obtained cyclic imine to its corresponding enamine as the last step (**Path A**). Simultaneously, one of the carbonyl groups in the intermediate **III** can be activated by the catalyst **1** to form the corresponding imine (intermediate **V**). Then, hetero-annaulation occurs by the intramolecular Michael addition to afford the desired 1,2,4-triazoloquinazolinones **5a–j** (**Path B**)^11,13,33,86–90^. It should be noted that the only byproducts of the reaction are water molecules with no environmental impact and can be dissolved in EtOH, as a green solvent, to promote the reaction efficiently by the nano-ordered Melamine@TMG organocatalyst (**1**).

**Figure 6:**
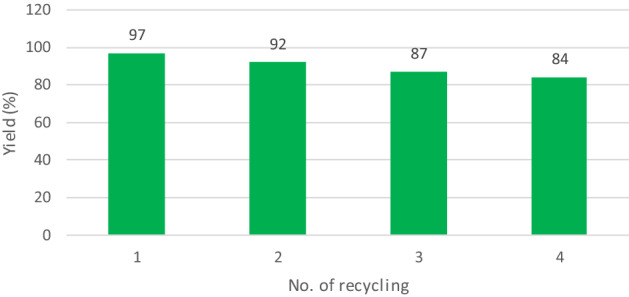
Recycling chart for the Melamine@TMG nanocatalyst **1** in the synthesis of **5a**.

As a part of our study, the recyclability of Melamine@TMG organocatalyst (**1**) was also examined in the synthesis of model compound **5a**. The results are reported in Fig. 6. Indeed, it was observed that the catalytic activity of catalyst **1** changed a little after four consecutive runs using the recycled samples .

To show the merits of the nano-ordered Melamine@TMG organocatalyst (**1**) for the multicomponent synthesis of 1,2,4-triazoloquinazolinones in comparison to the previously reported catalytic systems, Table 3 compares the obtained results for the synthesis of model compound **5a**. It is obvious that the excellent yield of 1,2,4-triazoloquinazolinone **5a** was achieved using a low loading of the present catalyst in a green solvent or at a lower temperature compared to the other reported systems.

**Table 3:** Comparison of the catalytic activity of nano-ordered Melamine@TMG organocatalyst (**1**) versus some introduced catalysts for the synthesis of **5a**.

Entry	Catalyst (loading/solvent)	Temperature (°C)	Time (min)	Yield (%)	References
1	Melamine@TMG (2.5 mol%/EtOH)	40	10	98	This work
2	Nano-SiO_2_ (15 mol%/CH_3_CN)	r.t	30	96	^71^
3	*p*-Toluenesulfonic acid (15 mol%/CH_3_CN)	50	25	95	^73^
4	[Bmim]BF_4_ (15 mol%/Solvent-Free)	r.t	11	92	^77^
5	[C_4_(H-DABCO)_2_] [HSO_4_]_4_ (16 mg/Solvent-Free)	90	12	91	^67^
6	Molecular iodine (10 mol%/CH_3_CN)	Reflux	10	81	^76^
7	Anthranilic acid (30 mol%/EtOH)	80	10	95	^75^

## Conclusion

In general, the novel nano-ordered 1,1,3,3-tetramethylguanidine-functionalized melamine (Melamine@TMG) organocatalyst was prepared and adequately characterized by various spectroscopic or microscopic methods as well as analytical techniques. The basic property of the catalyst was significantly increased by the incorporation of 1,1,3,3-tetramethylguanidine moiety while melamine itself acts as a bifunctional organocatalyst. The nano-ordered multifunctional Melamine@TMG organocatalyst was successfully used, as an efficient recyclable catalyst, for the three-component synthesis of 1,2,4-triazoloquinazolinone scaffold from 3-amino-1,2,4-triazole, dimedone, and different aryl aldehydes under green and environmentally-benign conditions. This practical method afforded the corresponding products in high to excellent yields within short reaction times. Using a heterogeneous multifunctional nanocatalyst, simple work-up procedure with no need for chromatographic purification, affording highly selective conversion, and recyclability of the catalyst with only a small decrease in its activity are other main advantages of this new practical protocol for the synthesis of 1,2,4-triazoloquinazolinones.

## Supplementary Information

Supplementary Information.
