# Supplementary material for: Tetramethylguanidine-functionalized melamine as a multifunctional organocatalyst for the expeditious synthesis of 1,2,4-triazoloquinazolinones
Source: Sci Rep. 2021 Jul 14;11:14457. doi: 10.1038/s41598-021-91463-1 (PMC8280119; doi:10.1038/s41598-021-91463-1)

**Supporting Information**

**Tetramethylguanidine-Functionalized melamine as a multifunctional organocatalyst for the expeditious synthesis of 1,2,4-triazoloquinazolinones**

Mahnoush Keshavarz^a^, Mohammad G. Dekamin^b^*, Manouchehr Mamaghani^c^, Mohammad Nikpassand^a^

^a^Departement of Chemistry, Faculty of Basic Sciences, Rasht Branch, Islamic Azad University, P.O. Box 41335-3516, Rasht, Iran.

^b^Department of Chemistry, Iran University of Science and Technology, Tehran 16846-13114, Iran.

^c^Department of Chemistry, Faculty of Sciences, University of Guilan, P.O. Box 41335-1914, Rasht, Iran.

**Spectral data of the products 5a-j:**

6,6-Dimethyl-9-phenyl-5,6,7,9-tetrahydro-[1,2,4]triazolo[5,1-*b*]quinazolin-8(4*H*)-one (**5a**):

White solid; Mp 250–252 ^o^C; FTIR (KBr): 3246, 3427, 3041, 2969, 2931, 1641, 1574, 1450, 1364-1254, 729 cm^-1^; ^1^H NMR (500 MHz, DMSO-*d_6_*): δ_H_ 0.96 (s, 3H, CH_3_), 1.03 (s, 3H, CH_3_), 2.04 (d, *J* = 16.2 Hz, 1H, CH), 2.18 (d, *J* = 16.2 Hz, 1H, CH), 2.50-2.59 (m, 2H, CH), 6.20 (s, 1H, CH_benzylic_), 7.16-7.28 (m, 5H, ArH), 7.61 (s, 1H, CH) related to the tri-azole ring, 11.15 (s, 1H, NH); ^13^C NMR (125 MHz, DMSO-*d*_6_): δ_C_ 191.3, 149.3, 141.9, 139.7, 127.5, 126.9, 126.4, 104.1, 57.5, 49.4, 40.3, 31.6, 28.1, 26.5.

9-(2-Methoxyphenyl)-6,6-dimethyl-5,6,7,9-tetrahydro-[1,2,4]triazolo[5,1-*b*]quinazolin-8(4*H*)-one (**5b**):

White solid; Mp 242–244 ºC; FTIR (KBr): 3432, 3093, 3026, 2974, 2835, 1651, 1584, 1369, 1369-1245, 753 cm^-1^; ^1^H NMR (500 MHz, DMSO-*d_6_*): δ_H_ 0.92 (s, 3H, CH_3_), 1.03 (s, 3H, CH_3_), 1.99 (d, *J* = 14.2 Hz, 1H, CH), 2.19 (d, *J* = 14.2 Hz, 1H, CH), 2.41-2.57 (m, 2H, CH), 3.63 (s, 3H, CH_3_) Related to the Methoxy, 6.35 (s, 1H, CH) Related to the middle ring of the aldehyde junction, 6.86-6.90 (m, *2*H, ArH), 7.19-7.25 (m, 2H, ArH), 7.60 (s, 1H, CH) Related to the tri-azole ring, 11.02 (s, 1H, NH); ^13^C NMR (125 MHz, DMSO-*d_6_*): δ_C_ 192.1, 156.6, 150.2, 149.0, 146.9, 129.3, 128.6, 128.3, 119.3, 111.0, 110.9, 104.2, 54.8, 54.7, 49.4, 31.5, 28.3, 25.6.

9-(3,4-Dimethoxyphenyl)-6,6-dimethyl-5,6,7,9-tetrahydro- [1,2,4]triazolo-[5,1-*b*]quinazolin-8(4*H*)-one (**5c**):

White solid; Mp 225-227 ^o^C ; FTIR (KBr): 3255, 3461, 3088, 2940, 2840, 1660, 1574, 1412, 1364-1259, 753 cm^-1^; 1H NMR (500 MHz, DMSO-*d_6_*): δ_H_ 1.00 (s, 3H, CH_3_), 1.05 (s, 3H, CH_3_), 2.09 (d, *J =* 15.0 Hz, 1H, CH), 2.23 (d, *J =* 15.0 Hz, 1H, CH), 2.50-2.57 (m, 2H, CH), 3.69 (s, 6H, OCH_3_), 6.16 (s, 1H, CH_benzylic_), 6.68 (s, 1H, ArH), 6.82 (d, 2H, ArH), 7.68 (s, 1H, CH) related to the tri-azole ring, 11.08 (s, 1H, NH); ^13^C NMR (125 MHz, DMSO-*d*_6_): δ_C_ 192.4, 149.4, 147.8, 146.2, 133.6, 118.5, 111.01, 110.4, 105.1, 57.0, 55.0, 54.9, 49.3, 31.6, 28.1, 26.2; CHN analysis: C (calculated: 63.94%; found: 63.94%), H (calculated: 6.24%; found: 6.24%) N (calculated: 15.14%; found: 15.14%).

6,6-Dimethyl-9-(4-methylphenyl)-5,6,7,9-tetrahydro-[1,2,4]triazolo[5,1-*b*]quinazolin-8(4*H*)-one (**5d**):

White solid; Mp 262–264 ºC; FTIR (KBr): 3141, 3260, 3041, 2964, 2916, 1646, 1574, 1417, 1360-1250, 753 cm^-1^; ^1^H NMR (500 MHz, DMSO-*d_6_*): δ_H_ 0.96 (s, 3H, CH_3_), 1.03 (s, 3H, CH_3_), 2.07 (d, *J* = 15.5 Hz, 1H, CH), 2.20 (d, *J* = 15.5 Hz, 1H, CH), 2.32 (s, 3H, CH_3_), 2.50-2.59 (m, 2H, CH), 6.19 (s, 1H, CH) Related to the middle ring of the aldehyde junction, 7.08 (s, 4H, ArH), 7.69 (s, 1H, CH) Related to the tri-azole ring, 11.12 (s, 1H, NH); ^13^C NMR (125 MHz, DMSO-*d_6_*): δ_C_ 192.4, 149.7, 149.4, 146.3, 138.2, 136.4, 128.2, 126.3, 105.2, 57.2, 49.3, 31.6, 28.0, 26.3, 20.1.

9-(4-Chlorophenyl)-6,6-dimethyl-5,6,7,9-tetrahydro-[1,2,4]triazolo[5,1-*b*]quinazolin-8(4*H*)-one (**5e**):

White solid; Mp 303–305ºC; FTIR (KBr): 3098, 3241, 3036, 2959, 2916, 1641, 1579, 1417, 1364-1254, 839, 753cm^-1^; ^1^H NMR (500 MHz, DMSO-*d_6_*): δ_H_ 0.97 (s, 3H, CH_3_), 1.05 (s, 3H, CH_3_), 2.10 (d, *J* = 15.5 Hz, 1H, CH), 2.22 (d, *J* = 15.5 Hz, 1H, CH), 2.32 (s, 3H, CH_3_), 2.51-2.55 (m, 2H, CH), 6.23 (s, 1H, CH) Related to the middle ring of the aldehyde junction, 7.22 (d, 2H, ArH), 7.34 (d, 2H, ArH), 7.69 (s, 1H, CH) Related to the tri-azole ring, 11.11 (s, 1H, NH); ^13^C NMR (125 MHz, DMSO-*d_6_*): δ_C_ 192.4, 150.0, 149.6, 146.3, 140.0, 131.8, 128.3, 127.7, 104.7, 56.9, 49.3, 31.6, 27.8, 26.4.

6,6-Dimethyl-9-(3-nitrophenyl)-5,6,7,9-tetrahydro-[1,2,4]triazolo[5,1-*b*]quinazolin-8(4*H*)-one (**5f**):

White solid; Mp 267-269 ºC; FTIR (KBr): 3427, 3241, 3121, 3084, 2959, 1651, 1584, 1517, 1422, 1364-1259, 724 cm^-1^; ^1^H NMR (500 MHz, DMSO-*d_6_*): δ_H_ 0.98 (s, 3H, CH_3_), 1.05 (s, 3H, CH_3_), 2.11 (d, *J* = 15.2 Hz, 1H, CH), 2.23 (d, *J* = 15.2 Hz, 1H, CH), 2.50-2.59 (m, 2H, CH), 6.43 (s, 1H, CH) Related to the middle ring of the aldehyde junction, 7.60-7.72 (m, 3H, ArH), 8.07 (s, 1H, CH) Related to the tri-azole ring, 8.24 (s, 1H, ArH) ,11.25 (s, 1H, NH); ^13^C NMR (125 MHz, DMSO-*d_6_*): δ_C_ 192.5, 150.6, 149.9, 147.1, 146.3, 142.9, 133.2, 129.5, 122.2, 121.2, 104.1, 56.9, 49.2, 31.7, 27.8, 26.3.

6,6-Dimethyl-9-(4-nitrophenyl)-5,6,7,9-tetrahydro-[1,2,4]triazolo[5,1-*b*]quinazolin-8(4*H*)-one (**5g**):

White solid; Mp 294–297 ºC; FTIR (KBr): 3432, 3275, 3131, 2969, 2931, 1651, 1574, 1527, 1417, 1345-1250, 729 cm^-1^; ^1^H NMR (500 MHz, DMSO-*d_6_*): δ_H_ 0.96 (s, 3H, CH_3_), 1.05 (s, 3H, CH_3_), 2.09 (d, *J* = 16.2 Hz, 1H, CH), 2.22 (d, *J* = 16.2 Hz, 1H, CH), 2.51-2.61 (m, 2H, CH), 6.38 (s, 1H, CH) Related to the middle ring of the aldehyde junction, 7.49 (d, *J* = 8.5 ,2H, ArH), 7.74 (s, 1H, CH) Related to the tri-azole ring, 8.16 (d, *J* = 8, 2H, ArH) ,11.31 (s, 1H, NH); ^13^C NMR (125 MHz, DMSO-*d_6_*): δ_C_ 192.5, 150.5, 149.9, 147.9, 146.4, 146.3, 127.9, 123.0, 104.2, 57.0, 49.2, 31.7, 27.8, 26.4.

9-(4-Hydroxyphenyl)-6,6-dimethyl-5,6,7,9-tetrahydro-[1,2,4]triazolo[5,1-*b*]quinazolin-8(4*H*)-one (**5h**):

White solid; Mp 305–308 ºC; FTIR (KBr): 3212, 3012, 3093, 2931, 2869, 1656, 1574, 1331, 1417, 1331-1254, 720cm^-1^; ^1^H NMR (500 MHz, DMSO-*d_6_*): δ_H_ 0.98 (s, 3H, CH_3_), 1.04 (s, 3H, CH_3_), 2.08 (d, *J* = 16.2 Hz, 1H, CH), 2.21 (d, *J* = 16.2 Hz, 1H, CH), 2.49-2.58 (m, 2H, CH), 6.13 (s, 1H, CH) Related to the middle ring of the aldehyde junction, 6.67 (d, *J* = 7.5 ,2H, ArH), 7.01(d, *J* = 8 ,2H, ArH), 7.68 (s, 1H, CH) Related to the tri-azole ring, 9.42 (s, 1H, OH) ,11.06 (s, 1H, NH); ^13^C NMR (125 MHz, DMSO-*d_6_*): δ_C_ 192.4, 156.4, 149.5, 149.3, 146.2, 131.7, 127.6, 114.4, 105.4, 56.8, 49.3, 31.6, 28.0, 26.3.

9-(3,4-Dichlorophenyl)-6,6-dimethyl-5,6,7,9-tetrahydro-[1,2,4]triazolo[5,1-*b*]quinazolin-8(4*H*)-one (**5i**):

White solid; Mp 324–326 ^o^C; FTIR (KBr): 3246, 2959, 3098, 2888, 2840, 1651, 1412, 1336-1269, 772, 562 cm^-1^; ^1^H NMR (500 MHz, DMSO-*d_6_*): δ_H_ 1.00 (s, 3H, CH_3_), 1.05 (s, 3H, CH_3_), 2.06 (d, *J =* 16.0 Hz, 1H, CH), 2.20 (d, *J =* 16.0 Hz, 1H, CH), 2.51-2.59 (m, 2H, CH), 6. 57 (s, 1H, CH_benzylic_), 7.36 (d, *J =* 7.0 Hz , 2H, ArH), 7.50 (s,1H, ArH), 7.68 (s, 1H, CH) related to the tri-azole ring, 11.21 (s, 1H, NH); ^13^C NMR (125 MHz, DMSO-*d*_6_): δ_C_ 192.3, 150.7, 149.7, 146.4, 136.8, 132.7, 132.6, 131.3, 128.4, 126.8, 103.6, 49.3, 31.6, 27.9, 26.4; CHN analysis : N (calculated: 15.42%; found: 14.79%), C (calculated: 56.21%; found: 56.59%), H (calculated: 4.44%; found: 4.59%).

9-(4-Fluorophenyl)-6,6-dimethyl-5,6,7,9-tetrahydro-[1,2,4]triazolo[5,1-*b*]quinazolin-8(4*H*)-one (**5j**):

White solid; Mp 257–259 ^o^C; FTIR (KBr): 3131, 3084, 3031, 2964, 2883, 1656, 1584, 1407, 1360-1216, 1145, 758, cm^-1^; ^1^H NMR (500 MHz, DMSO-*d*_6_): δ_H_ 0.98 (s, 3H, CH_3_), 1.05 (s, 3H, CH_3_), 2.10 (d, *J =* 15.5 Hz, 1H, CH), 2.22 (d, *J =* 15.5 Hz, 1H, CH), 2.51-2.58 (m, 2H, CH), 6. 25 (s, 1H, CH_benzylic_), 7.10 (d, *J =* 8.0, 2H, ArH), 7.25 (d, *J =* 8.0, 2H, ArH), 7.69 (s, 1H, CH) related to the tri-azole ring, 11.09 (s, 1H, NH); ^13^C NMR (125 MHz, DMSO-*d*_6_): δ_C_ 192.4, 160.0, 149.9, 149.6, 146.3, 137.3, 128.4, 114.4, 104.9, 56.8, 49.3, 31.6, 27.9, 26.4.

**FTIR and NMR spectrum of all compounds**

**(5a)**

- FTIR spectrum of (**5a**)


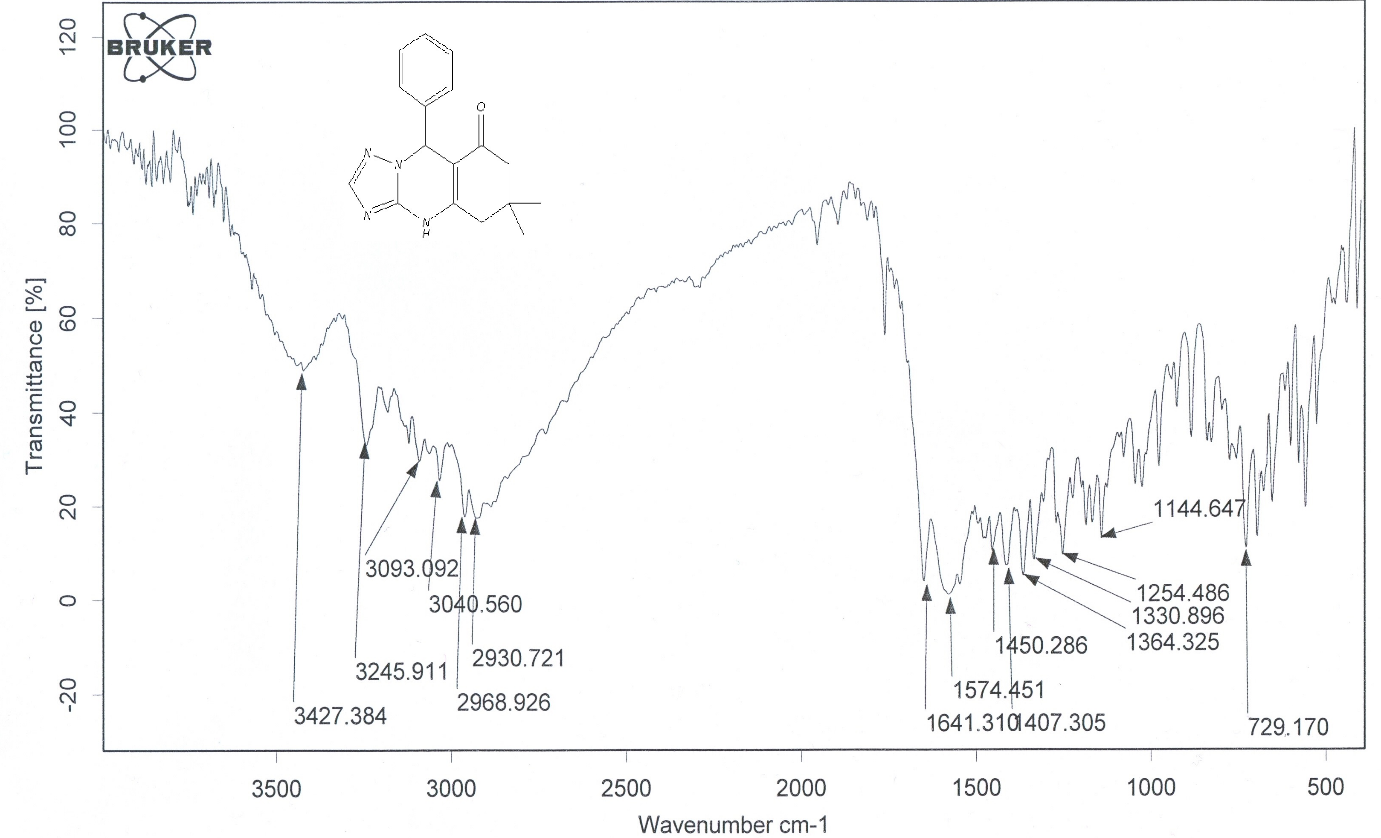


- ^1^H NMR spectrum of (**5a**, 500 MHz, DMSO-*d*_6_)
-
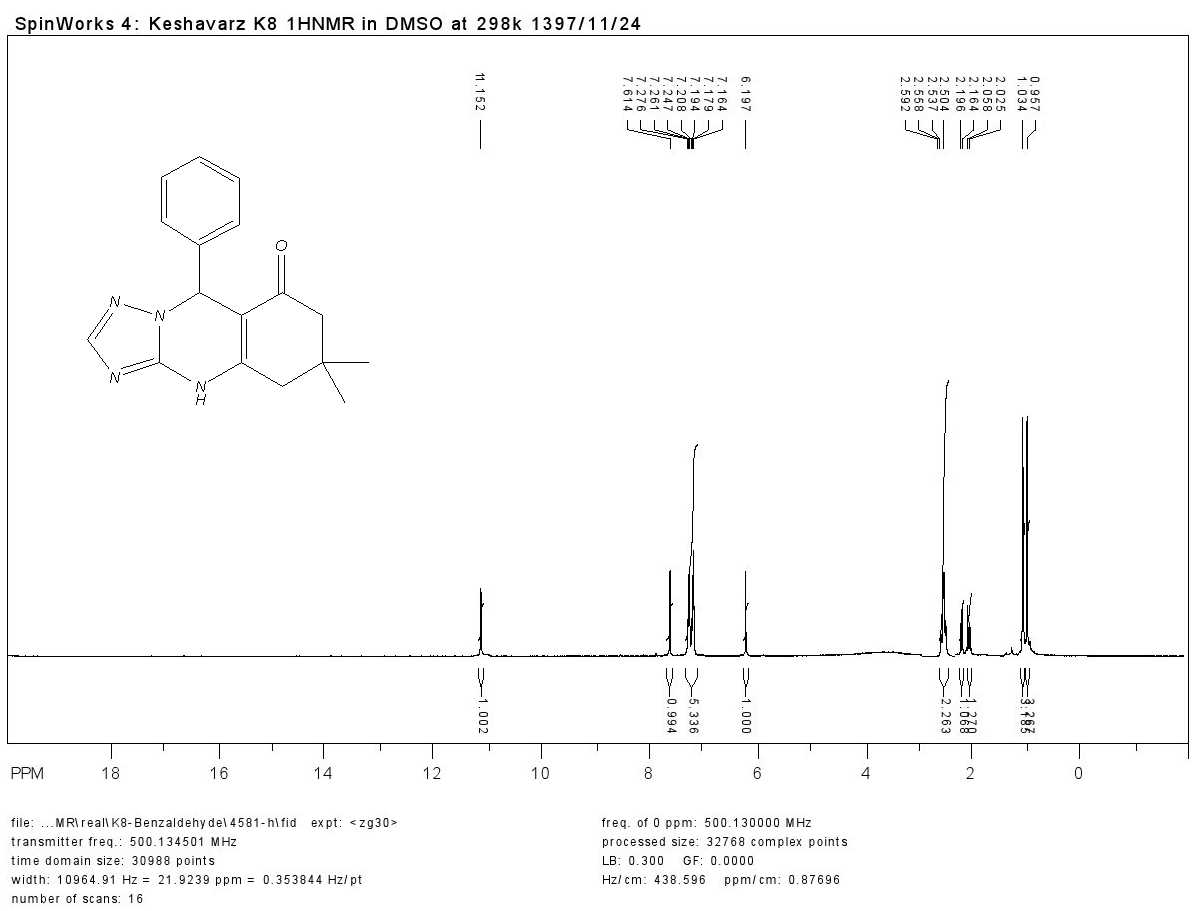


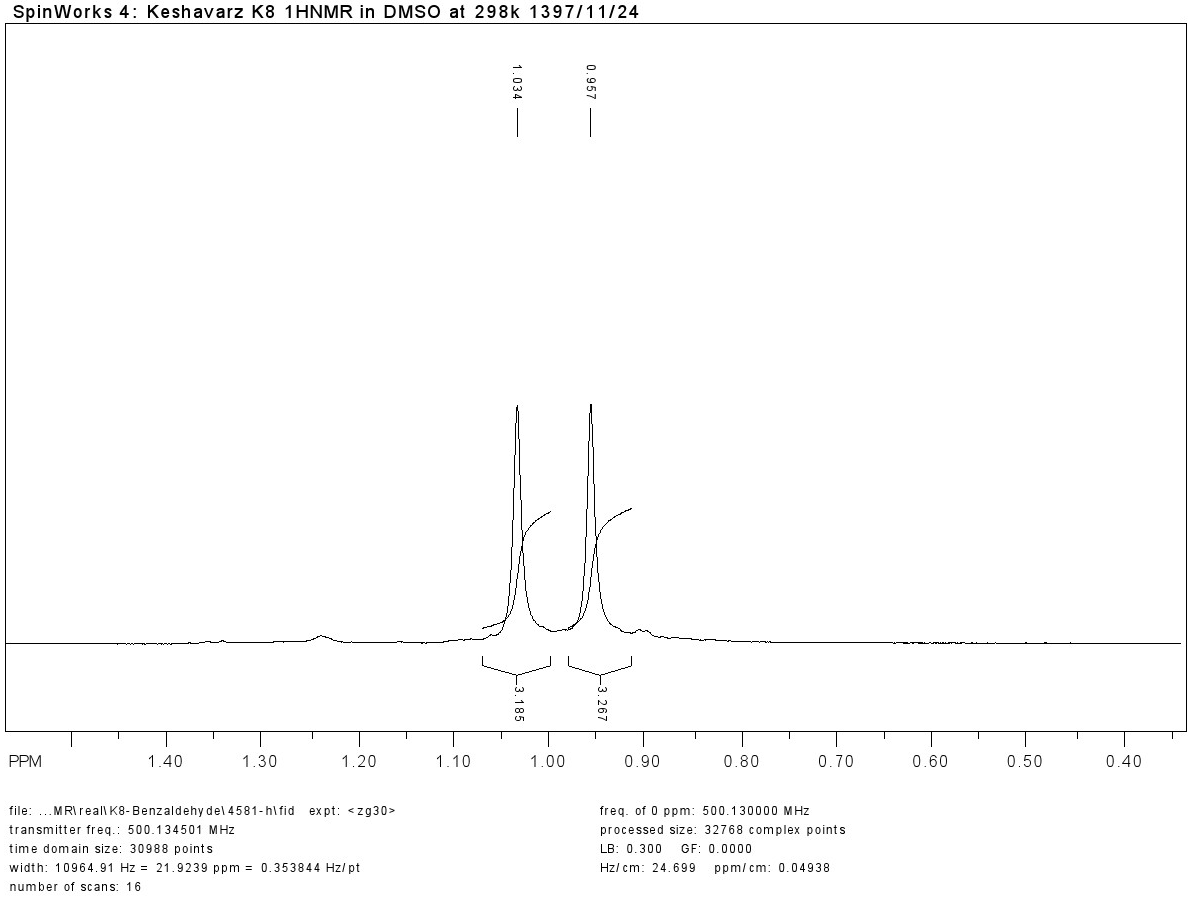


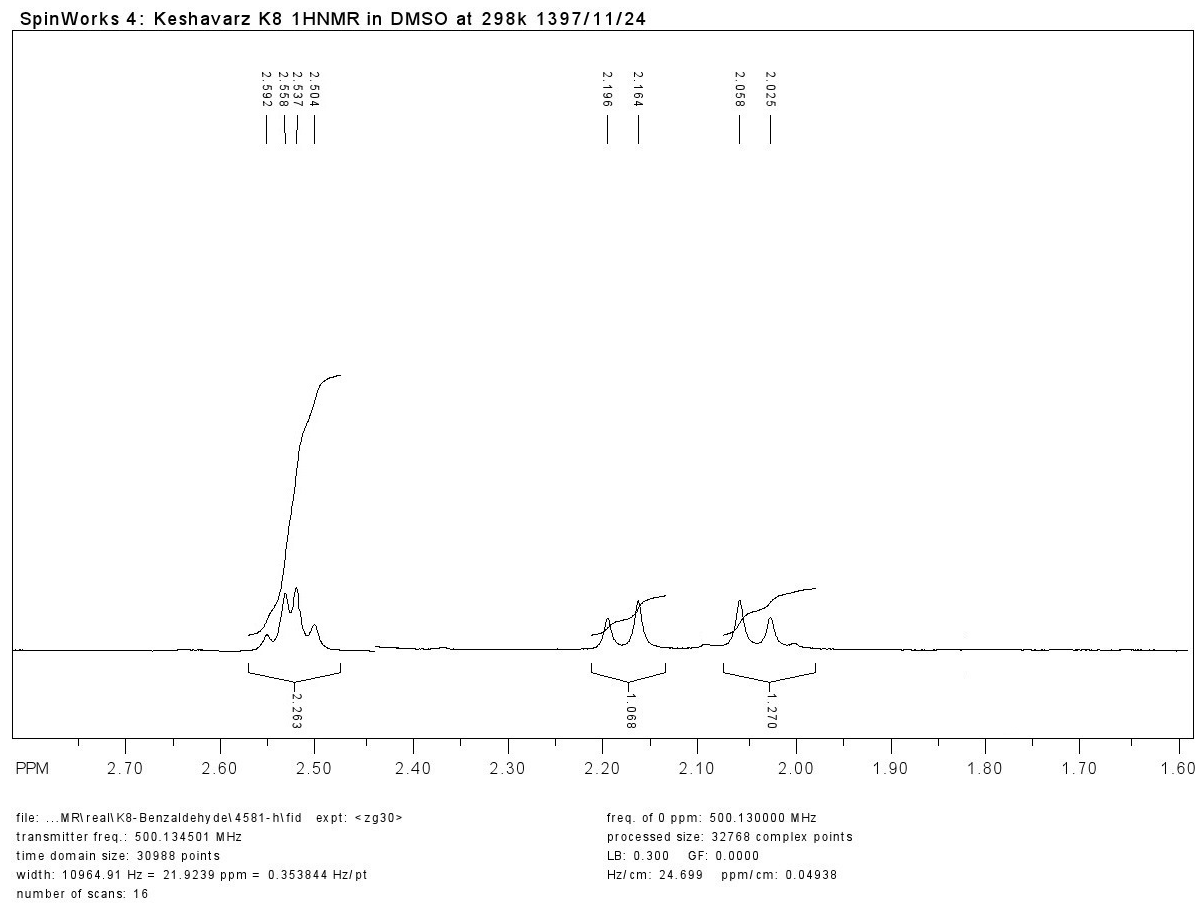


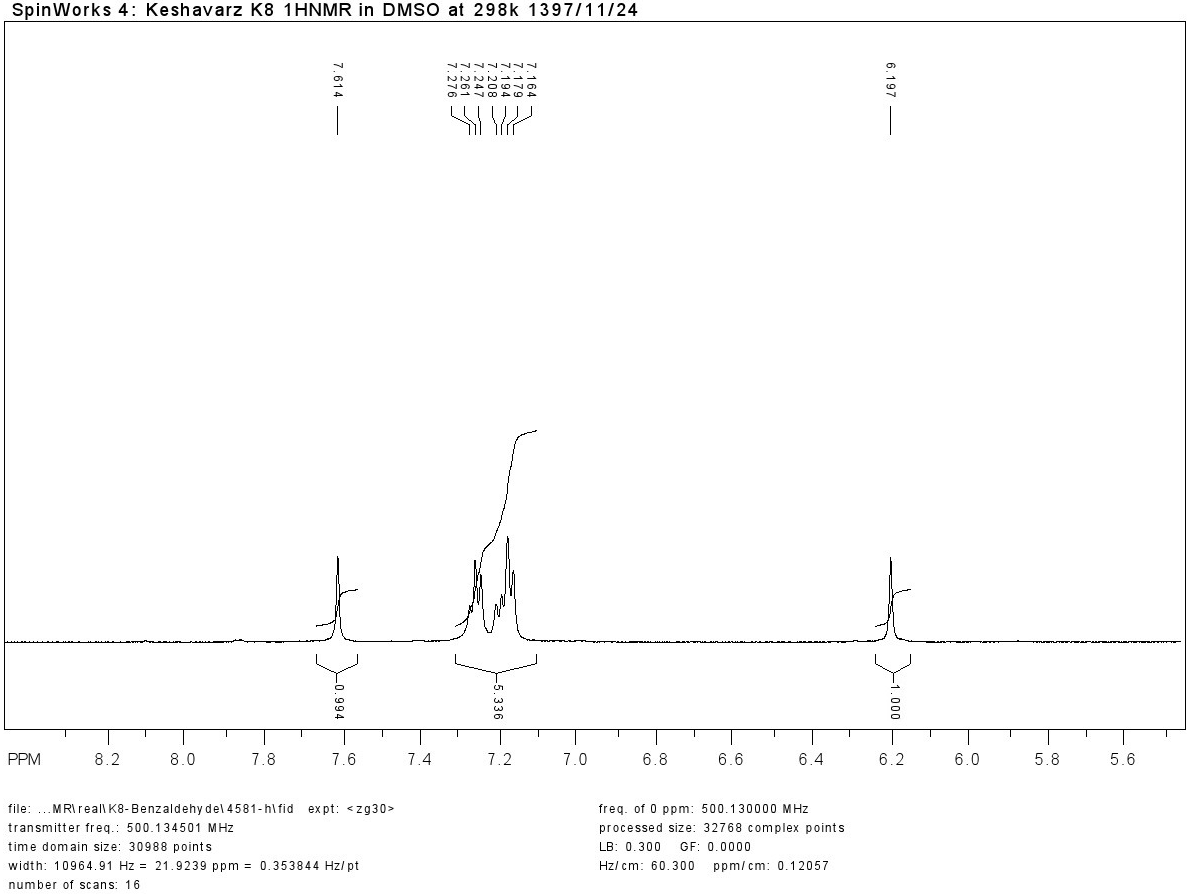


- ^13^C NMR spectrum of (**5a**, 125 MHz, DMSO-*d*_6_)


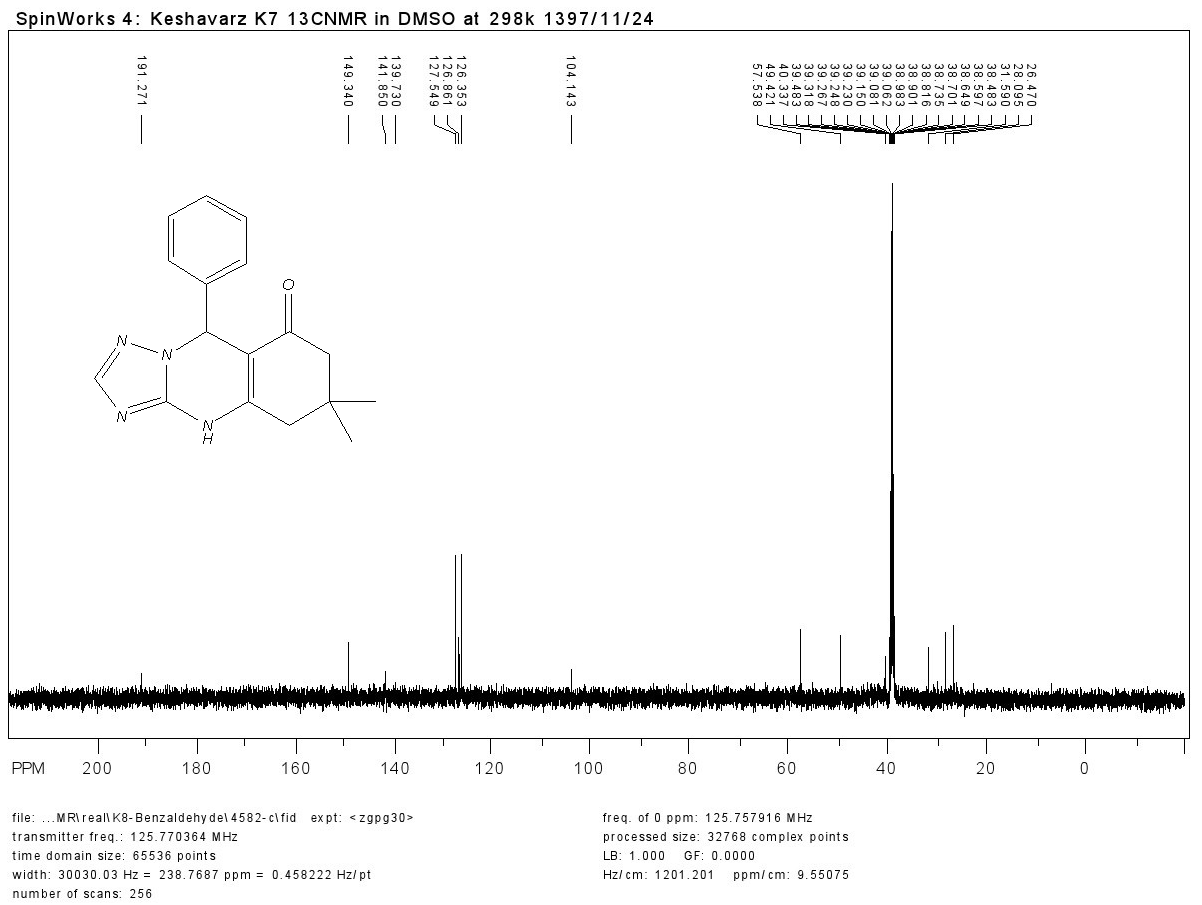


**(5b)**

- FTIR spectrum of (**5b**)


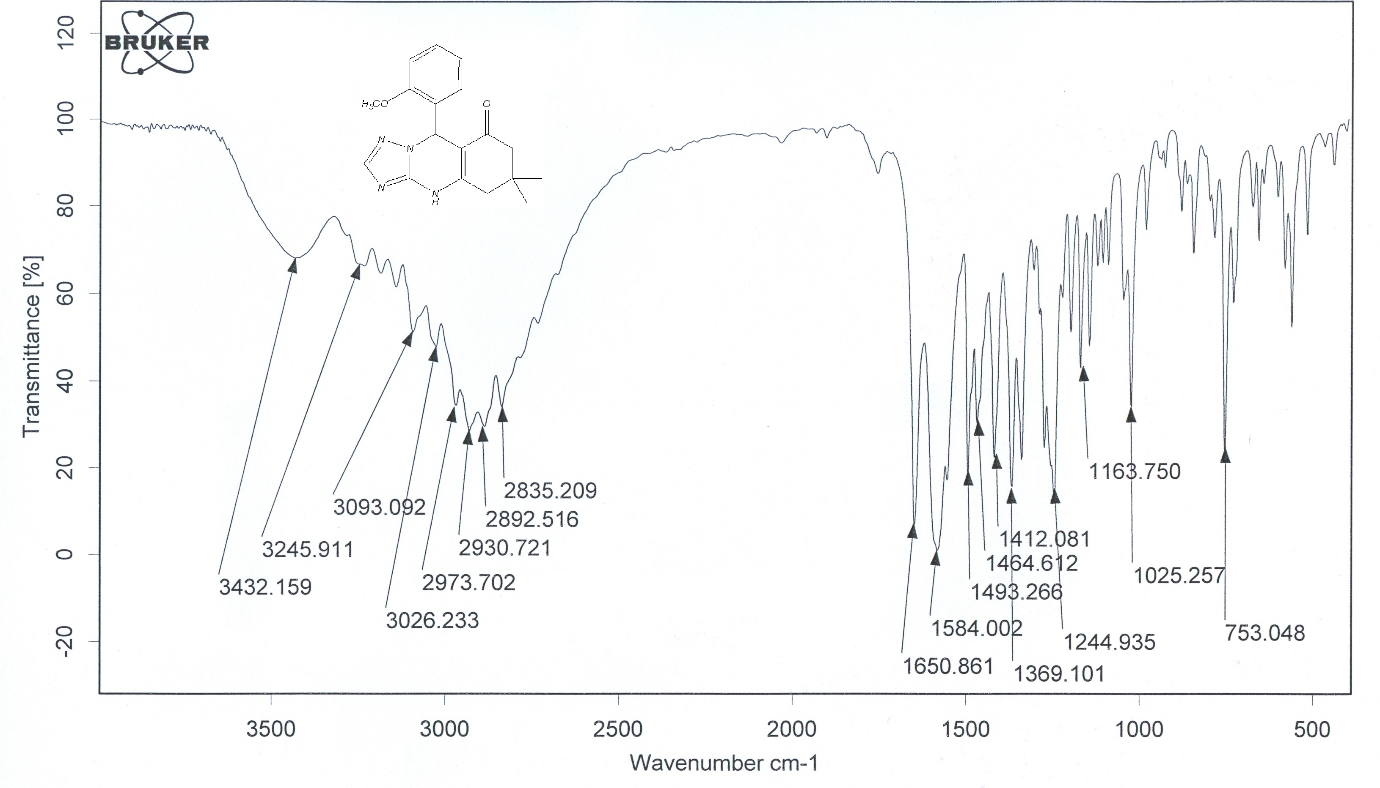


- ^1^H NMR spectrum of (**5b**, 500 MHz, DMSO-*d*_6_)


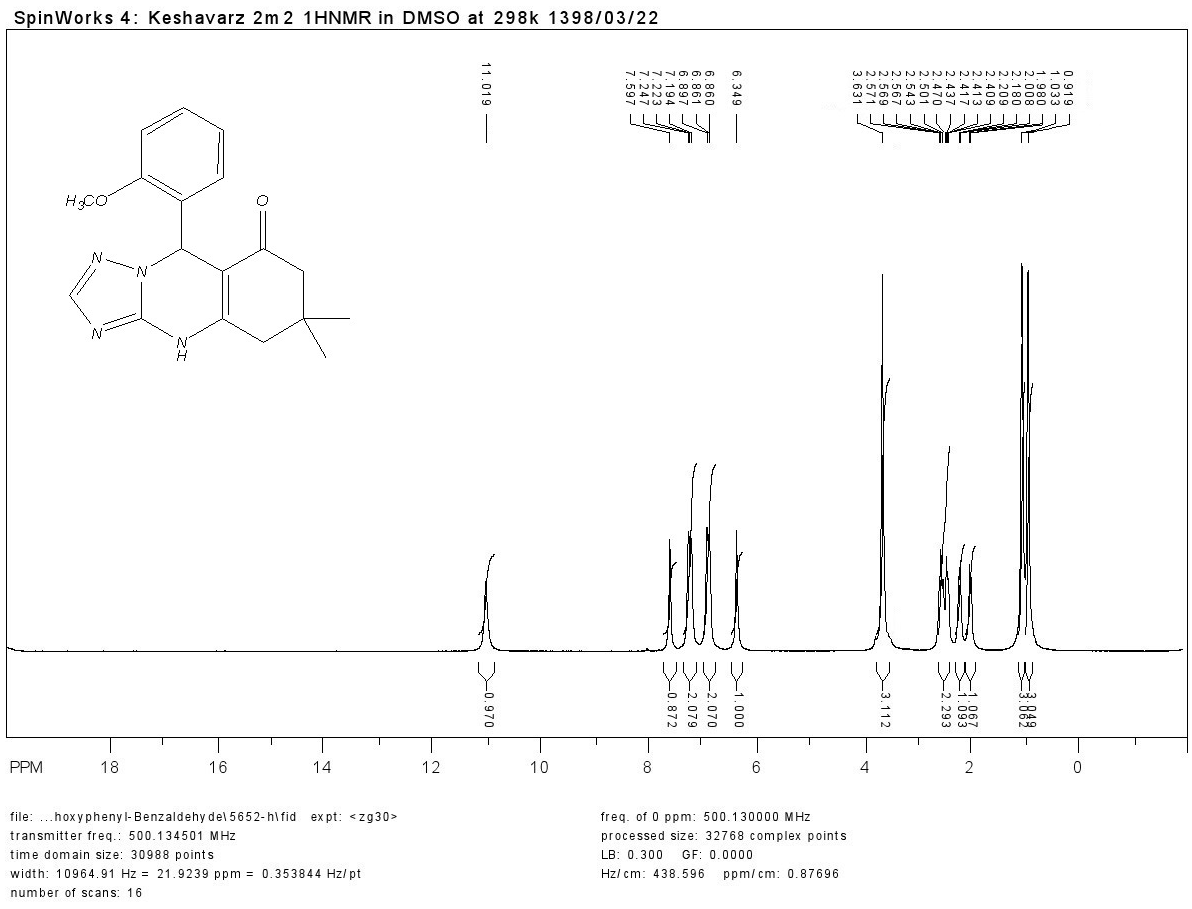


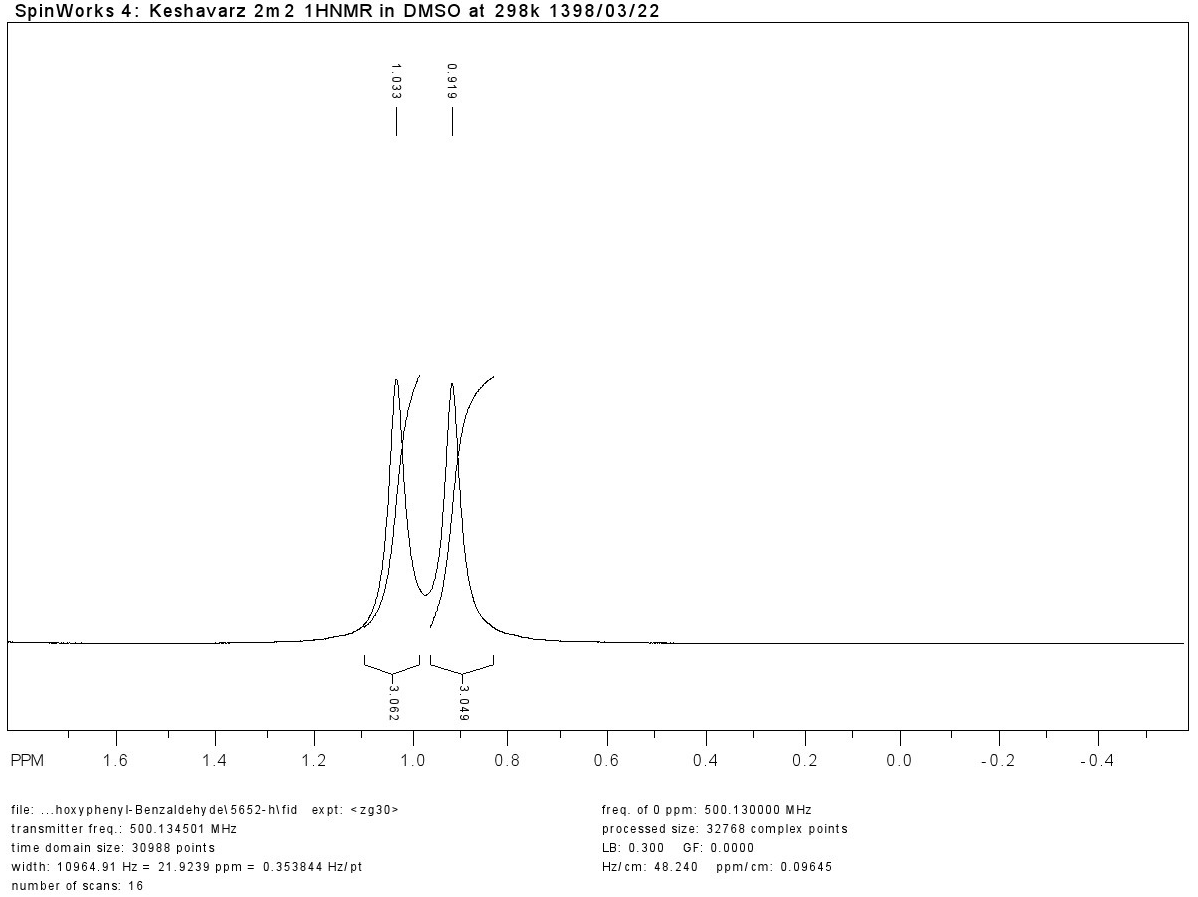


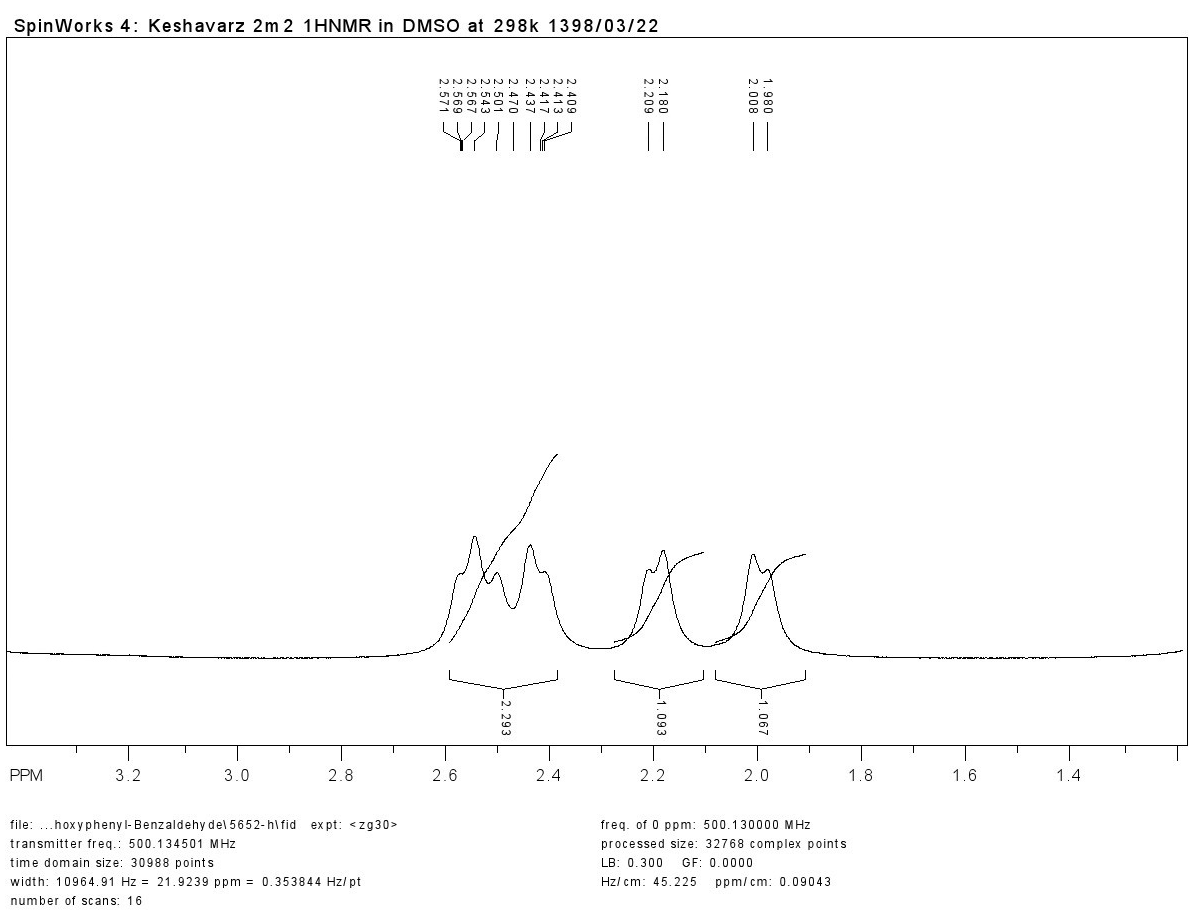


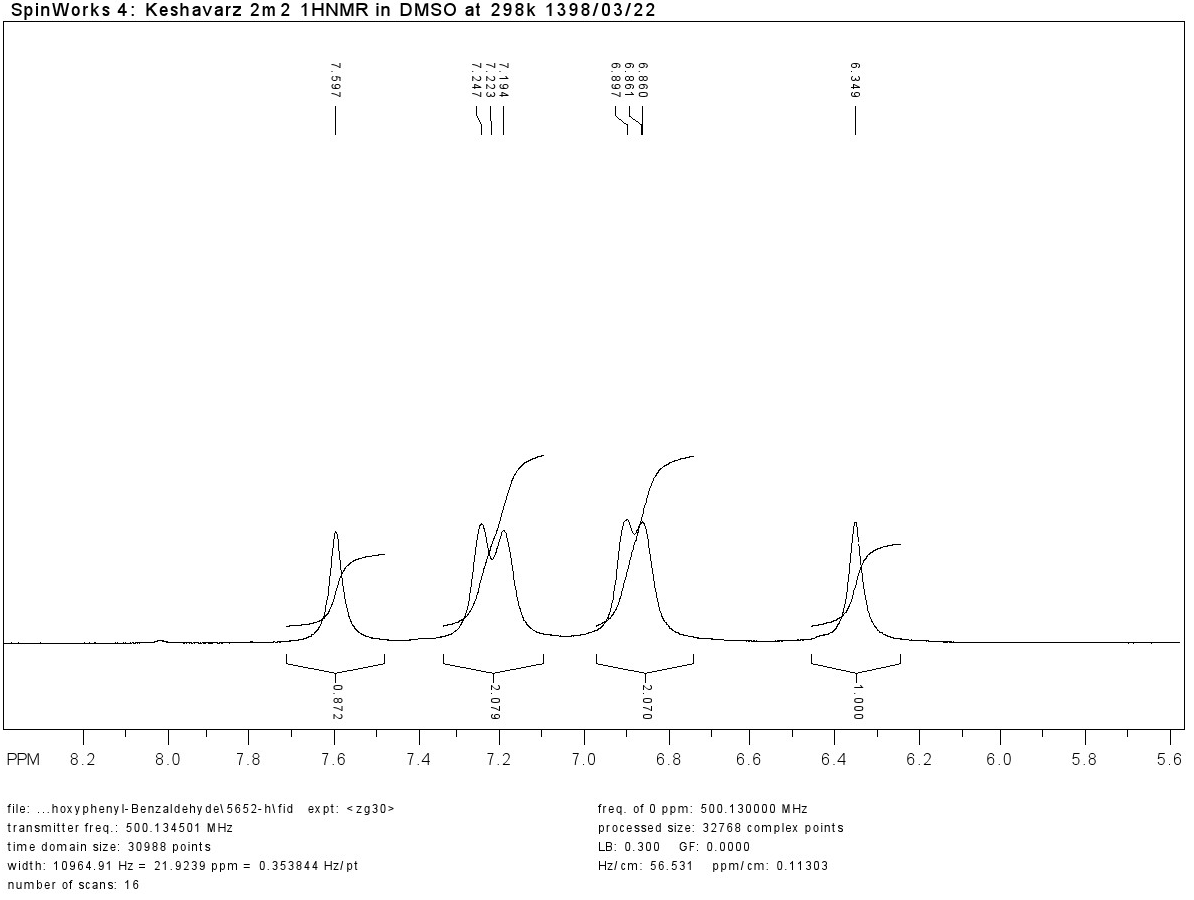


- ^13^C NMR spectrum of (**5b**, 125 MHz, DMSO-*d*_6_)


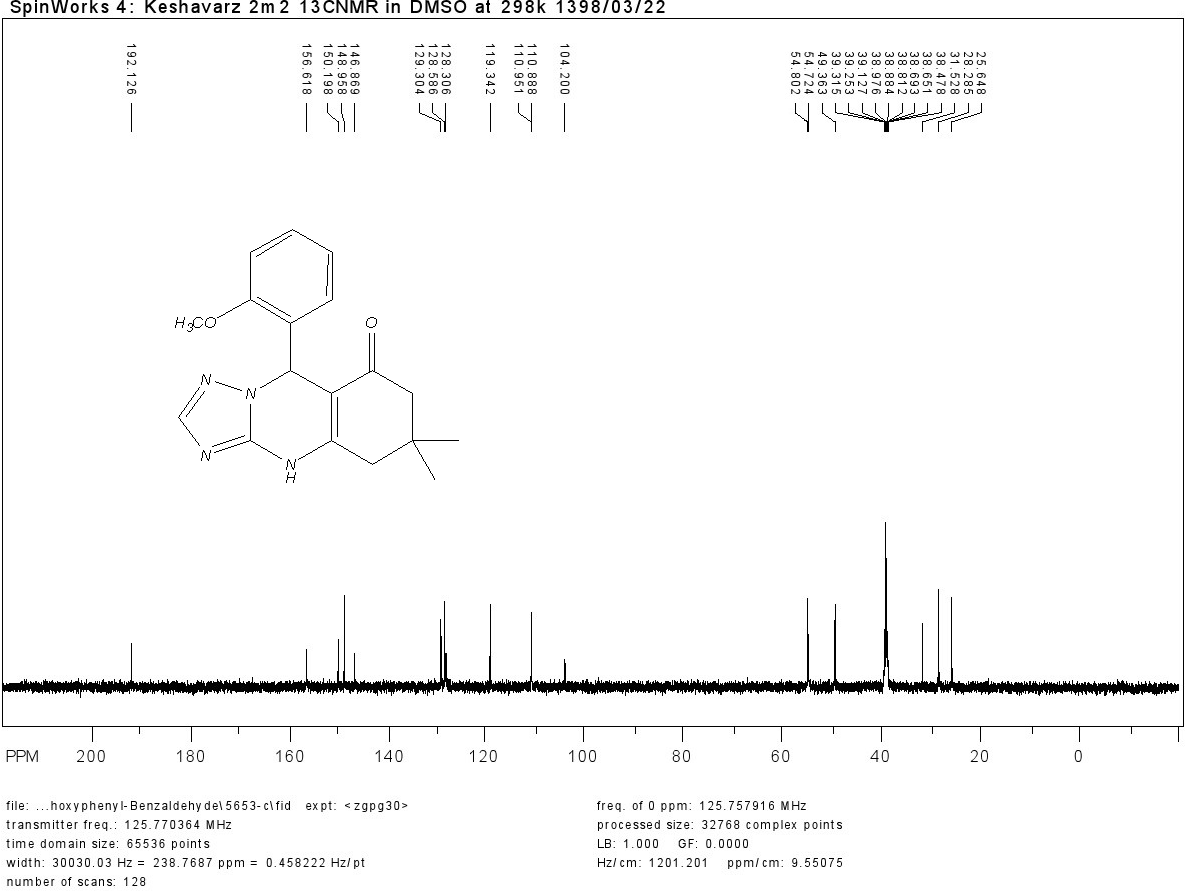


**(5c)**

- FTIR spectrum of (**5c**)


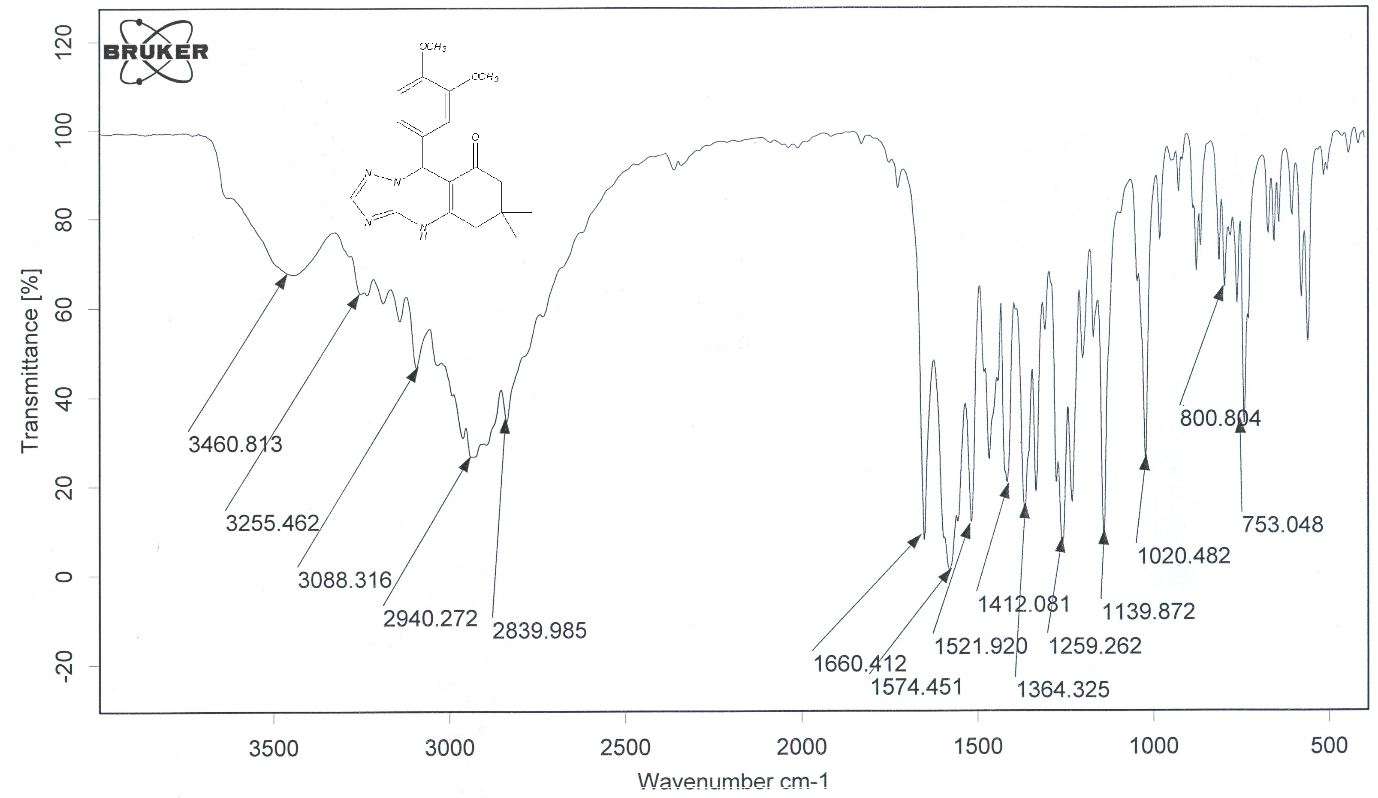


- ^1^H NMR spectrum of (**5c**, 500 MHz,DMSO-*d_6_*)


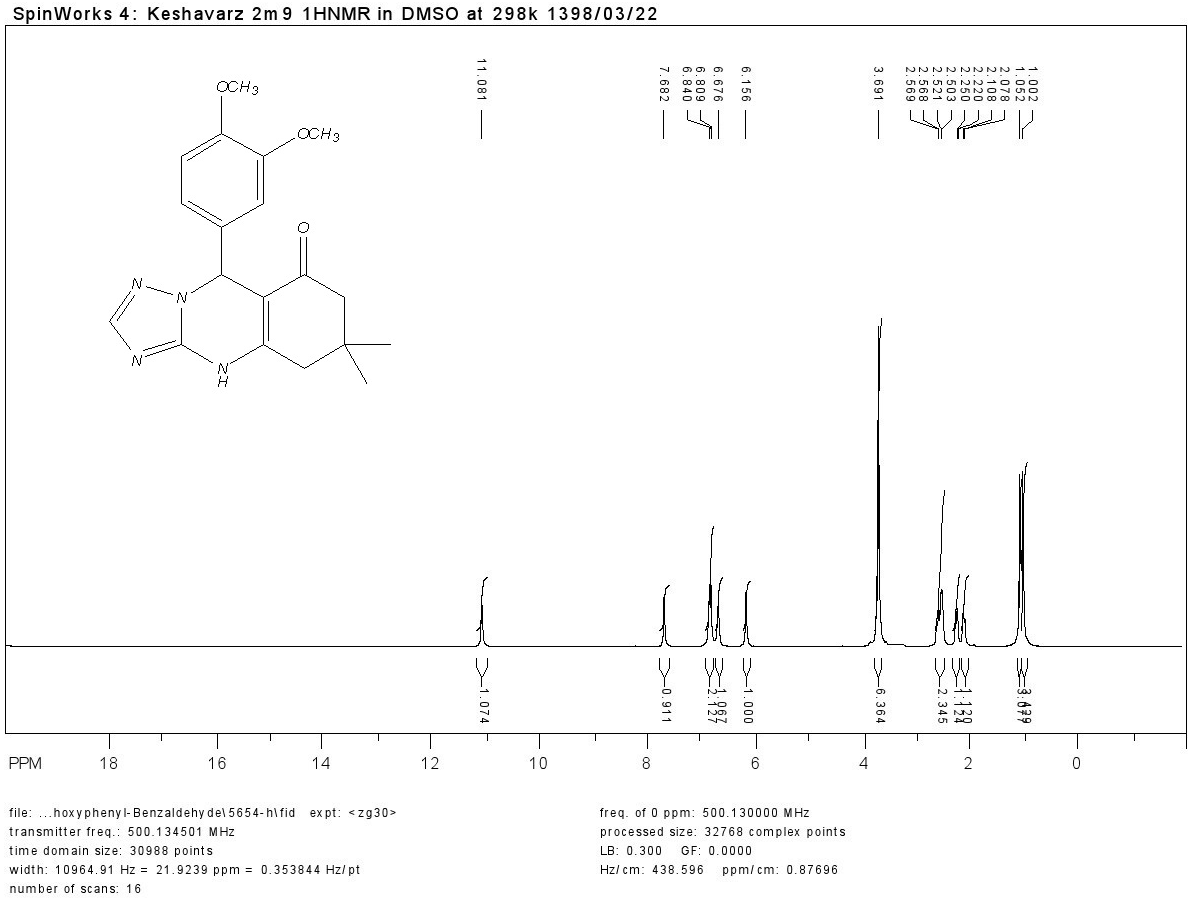


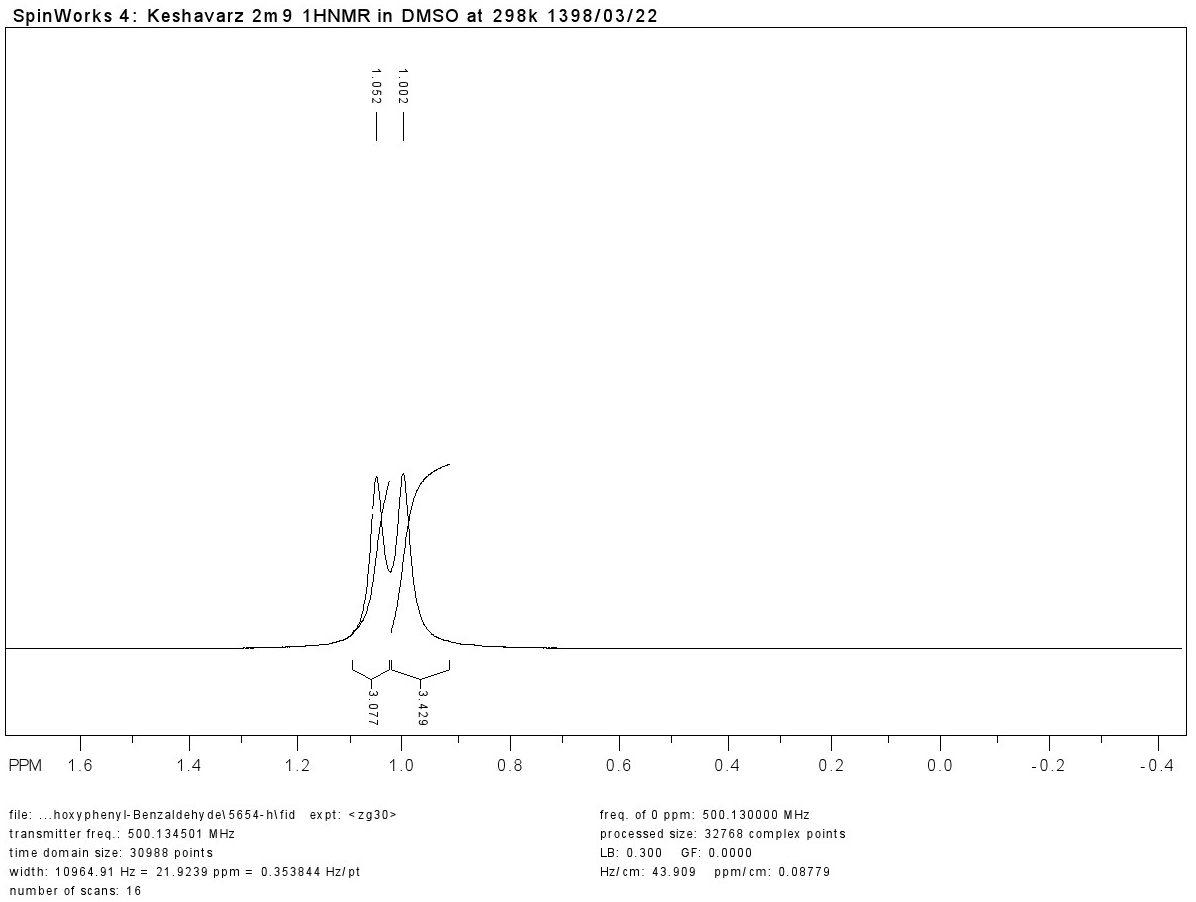


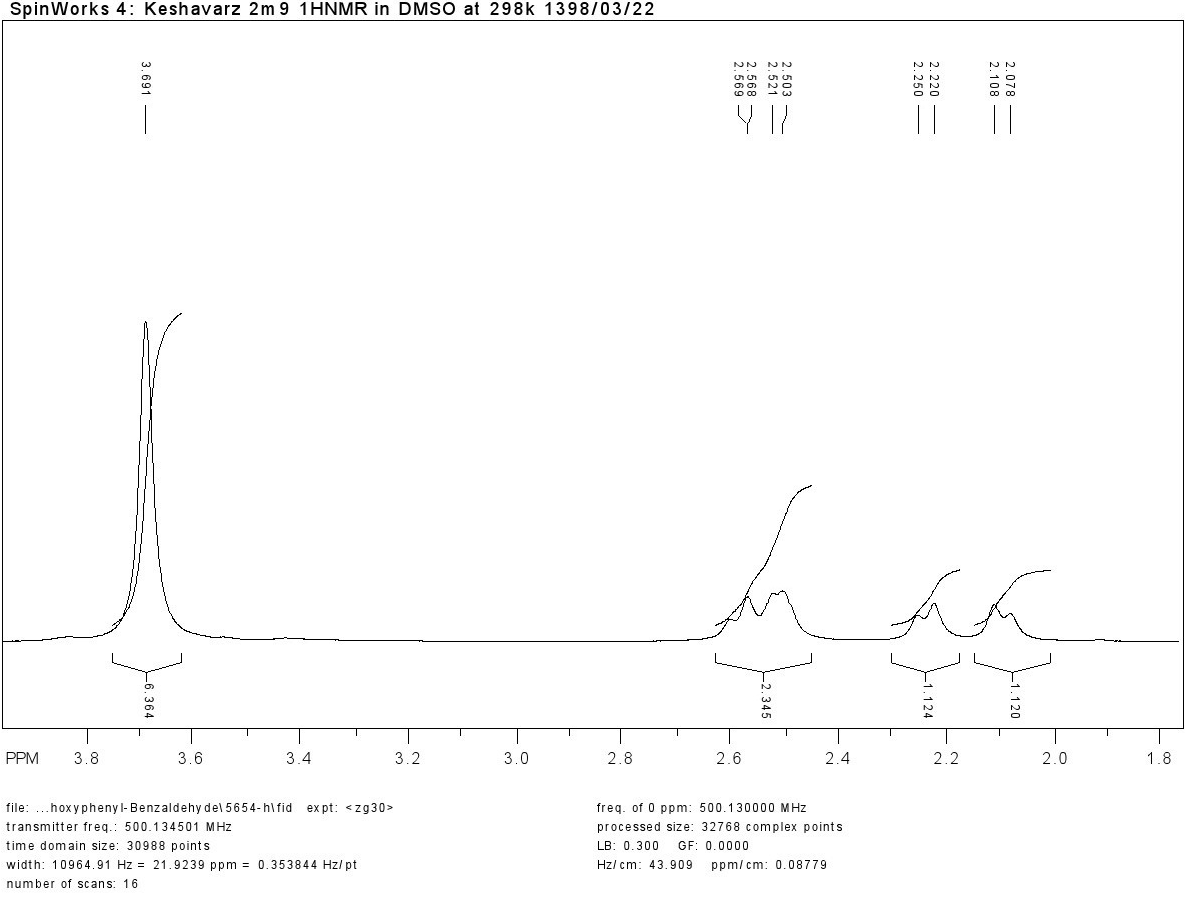


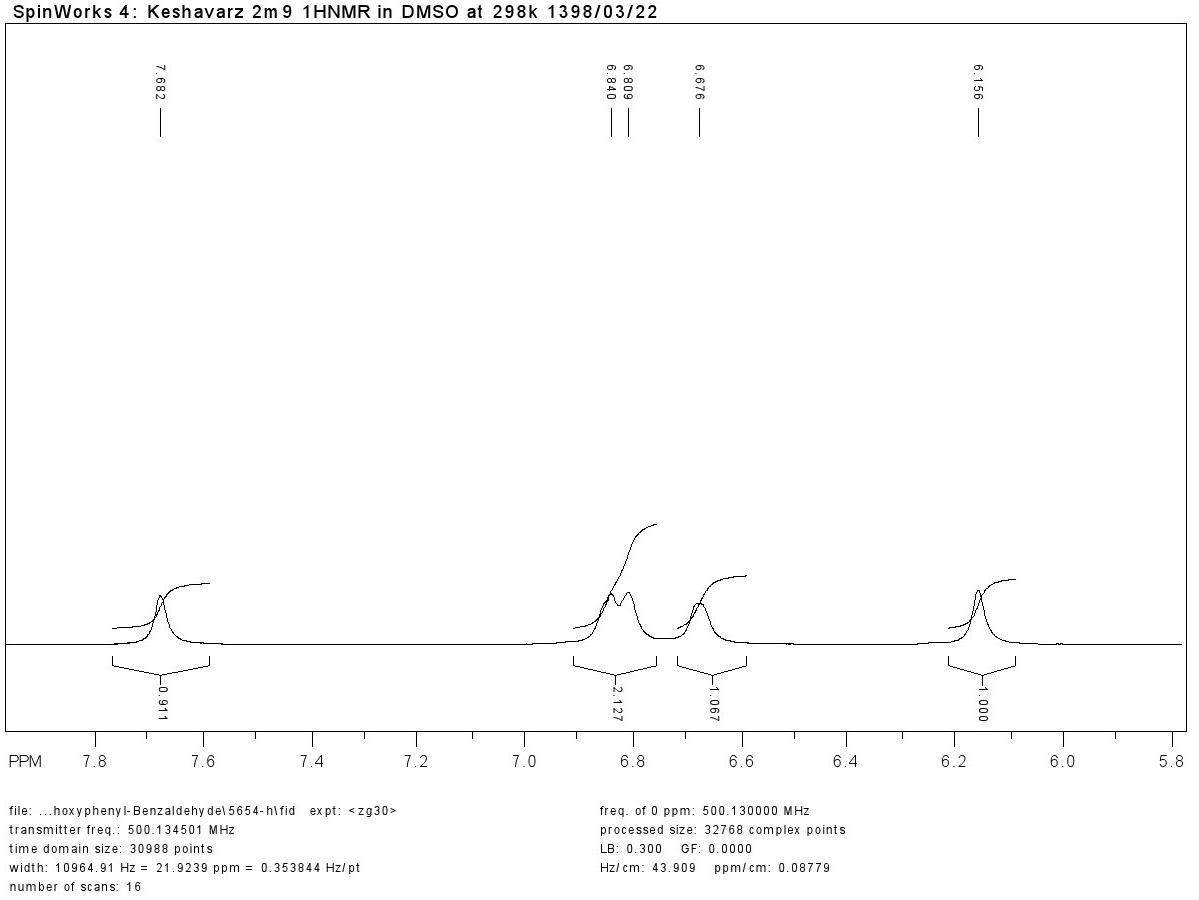


- ^13^C NMR spectrum of (**5c**, 125 MHz, DMSO-*d*_6_)


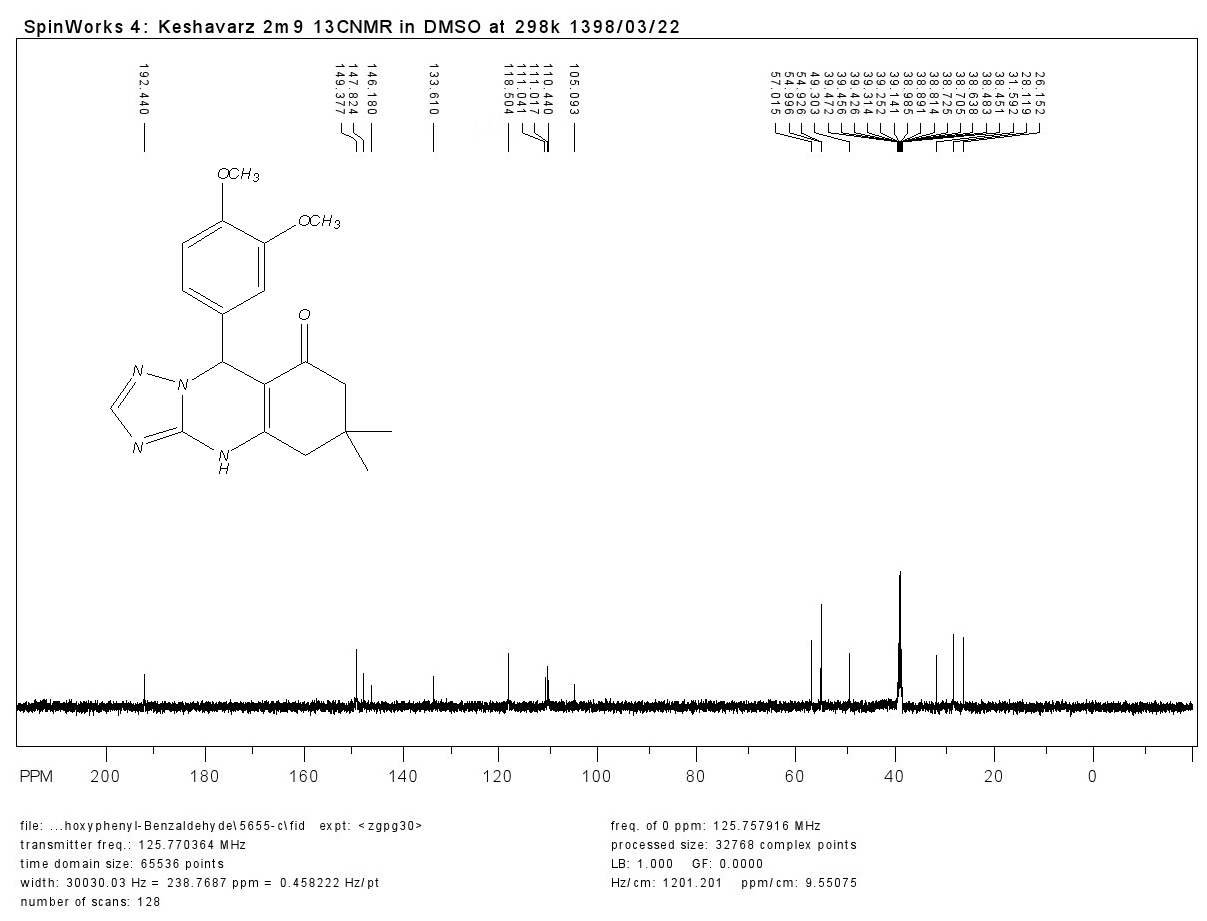


- CHN analysis of **5c**

**(5d)**

- FTIR spectrum of (**5d**)


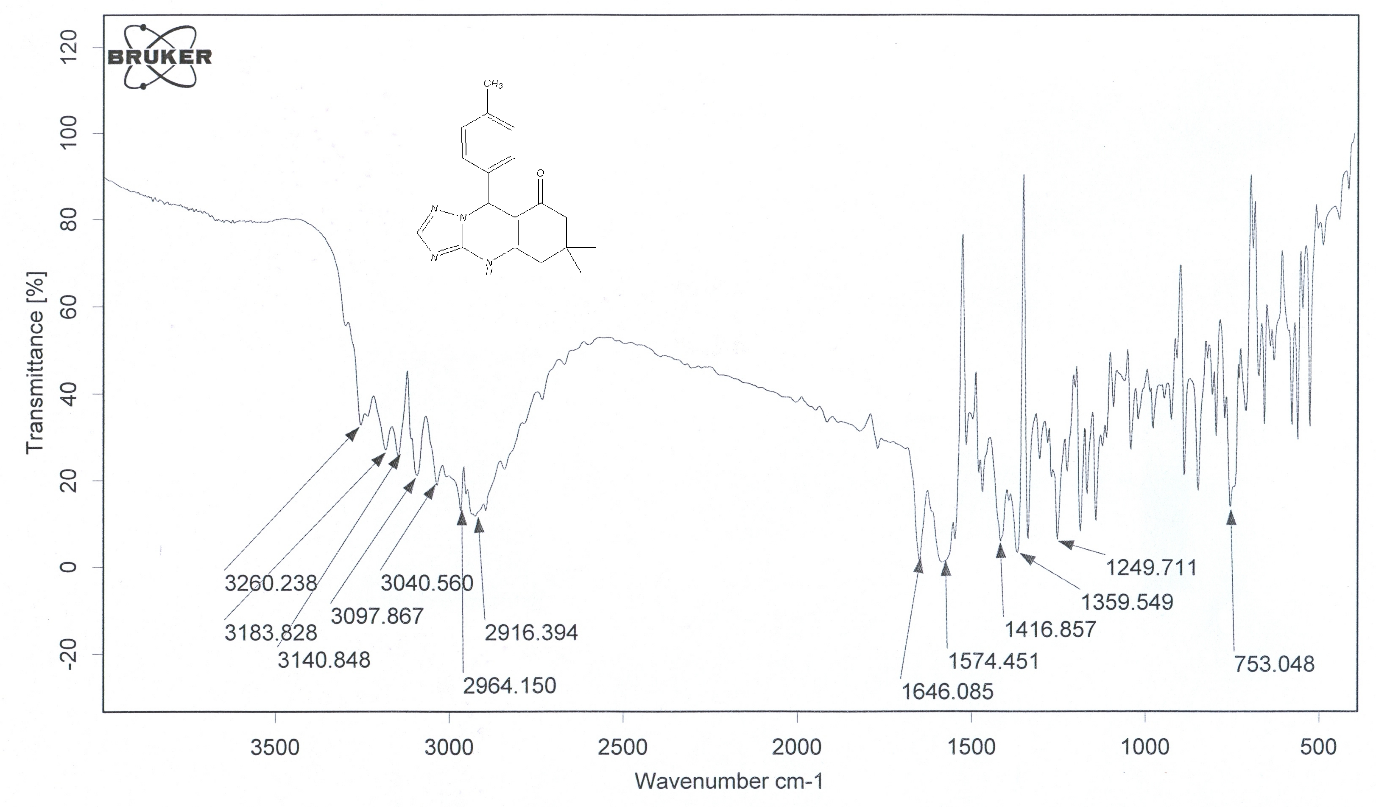


- ^1^H NMR spectrum of (**5d**, 500 MHz, DMSO-*d_6_*)


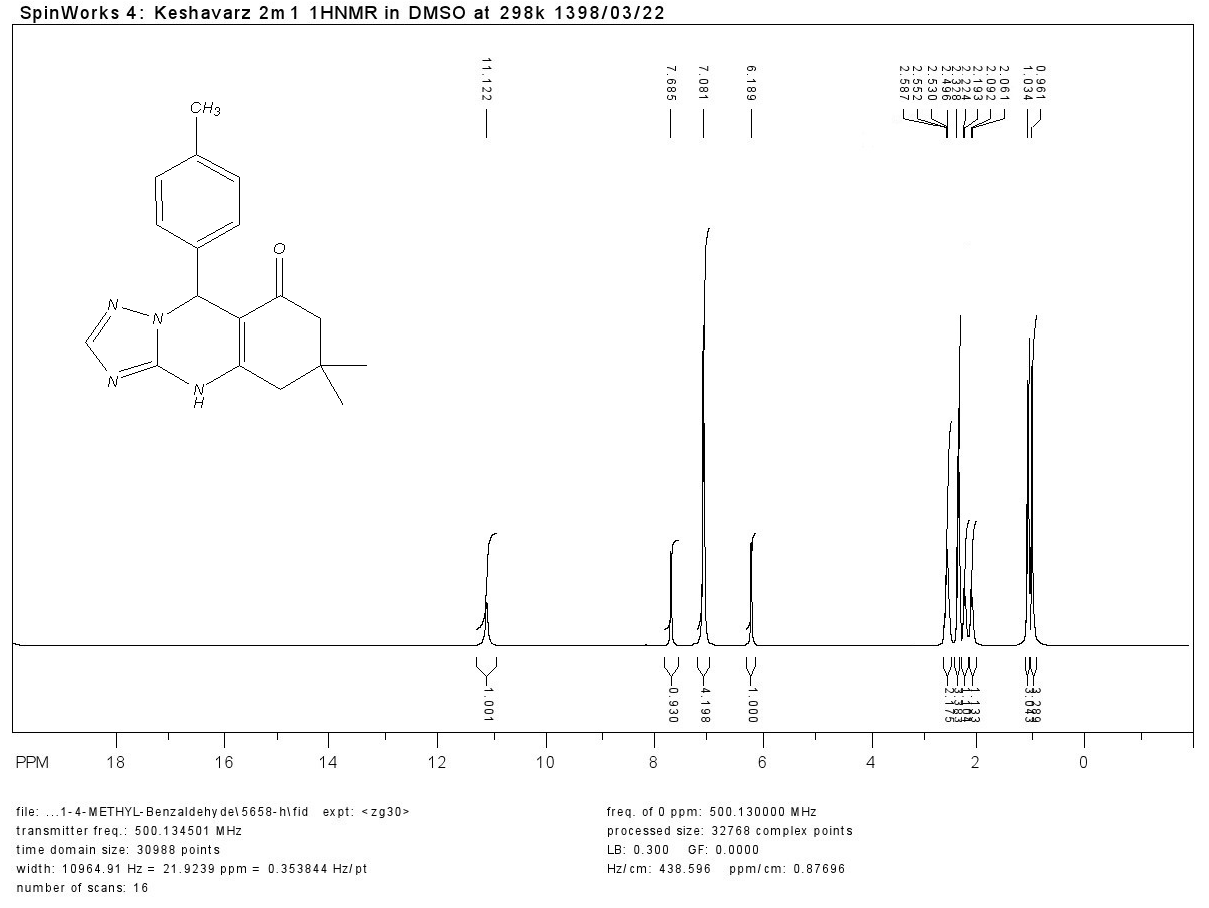


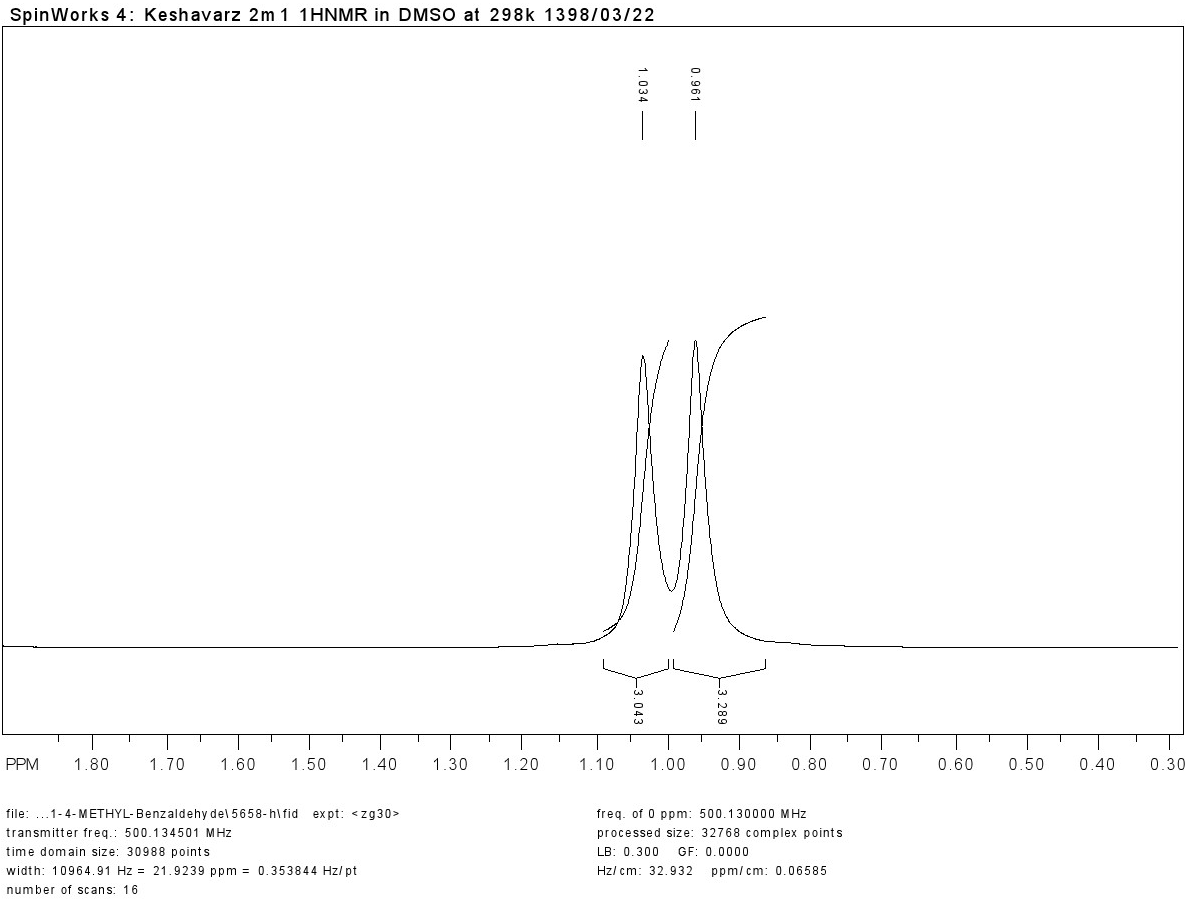


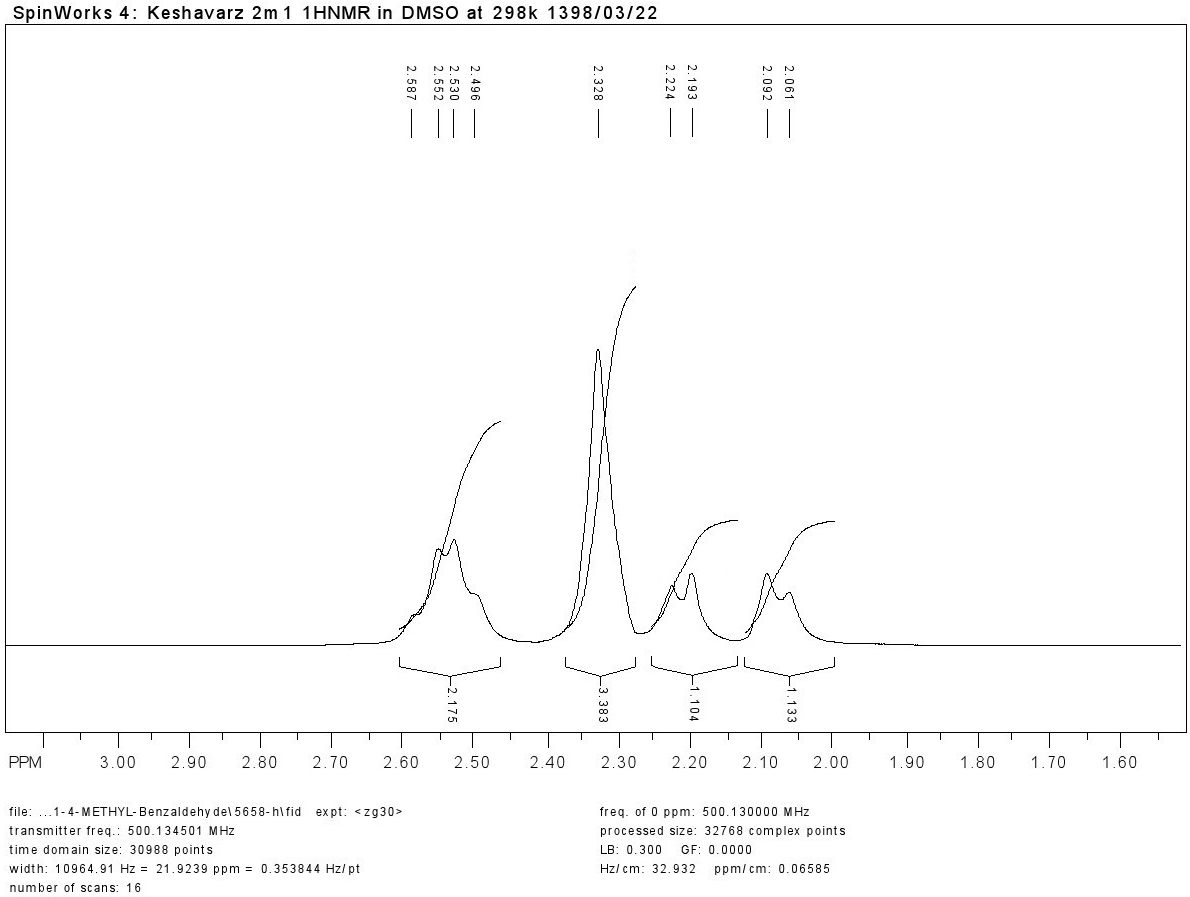


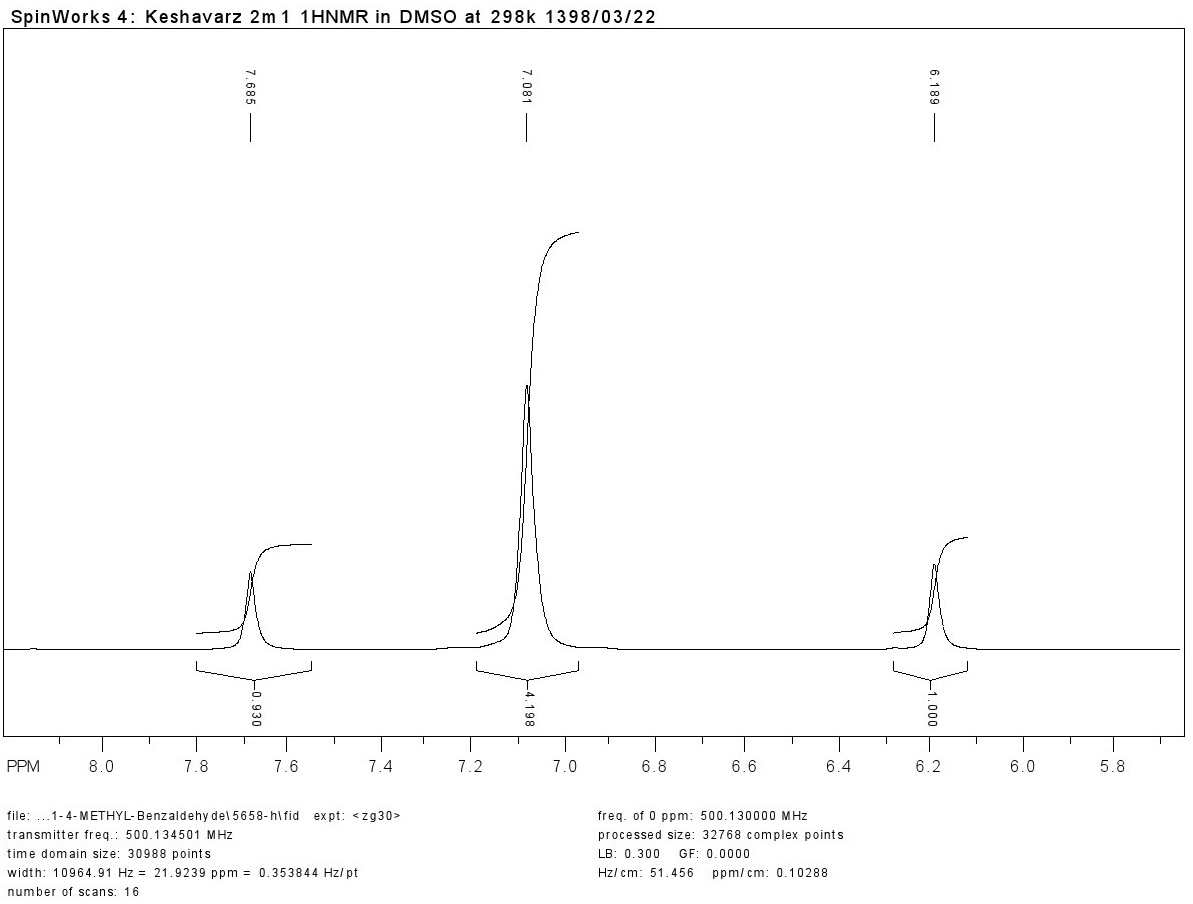


- ^13^C NMR spectrum of (**5d**, 125 MHz, DMSO-*d*_6_)


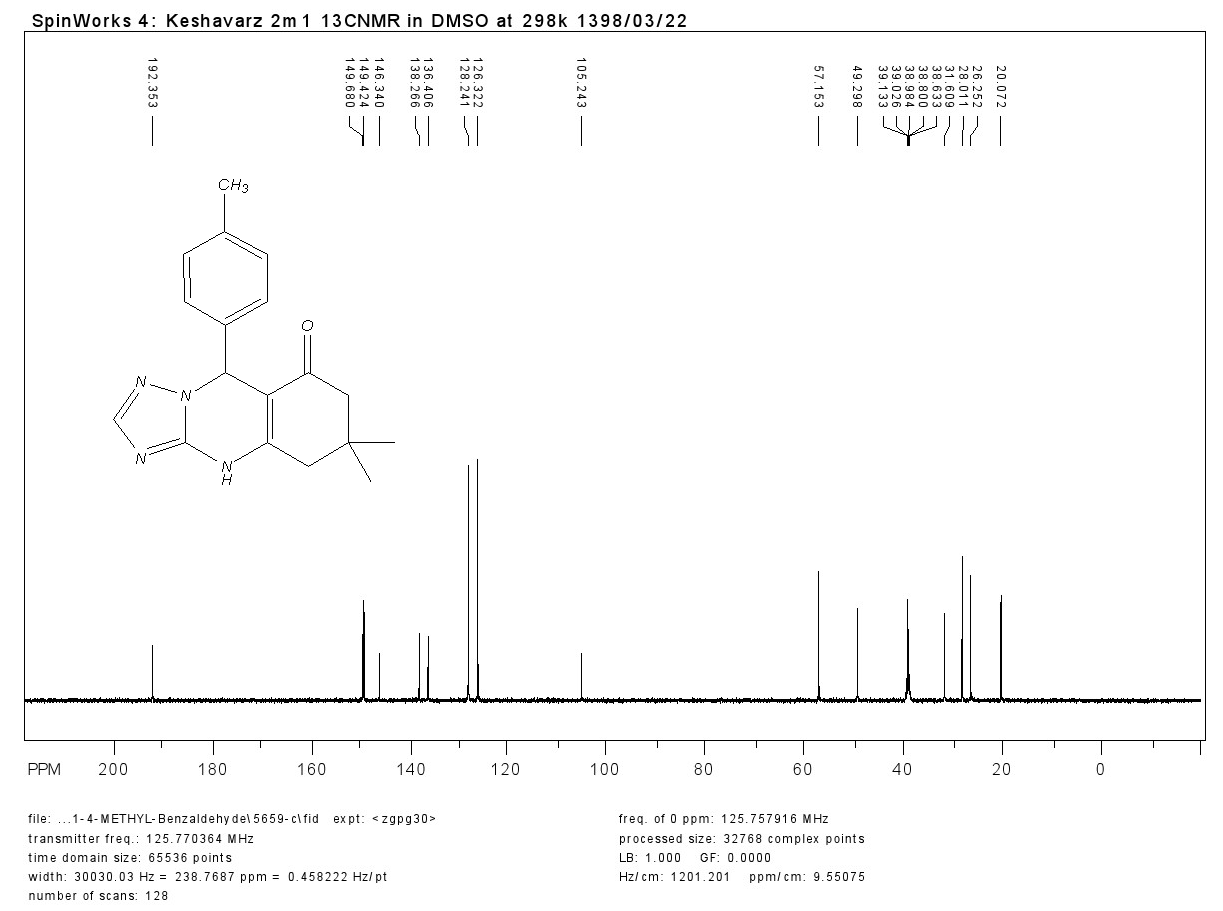


**(5e)**

- FTIR spectrum of (**5e**)


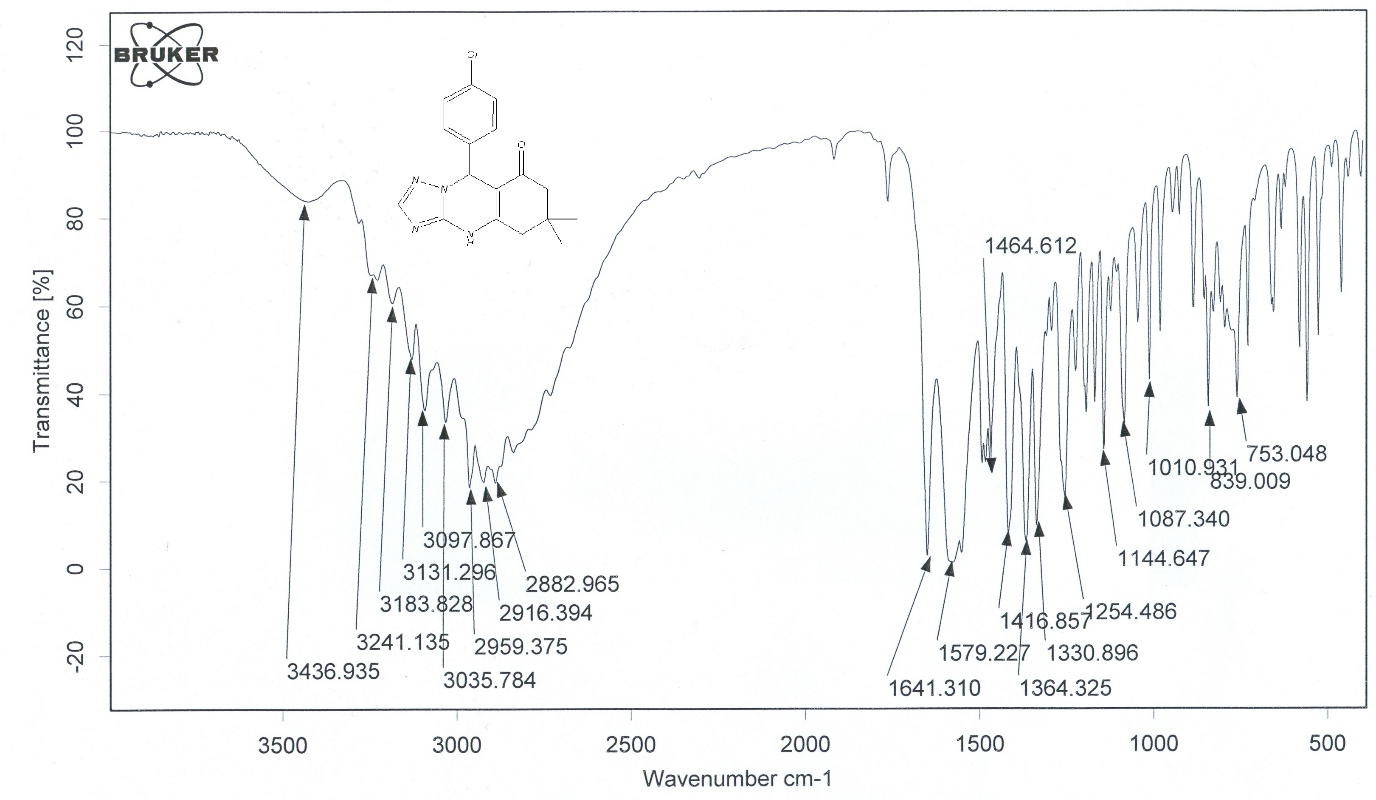


- ^1^H NMR spectrum of (**5e**, 500 MHz, DMSO-*d_6_*)


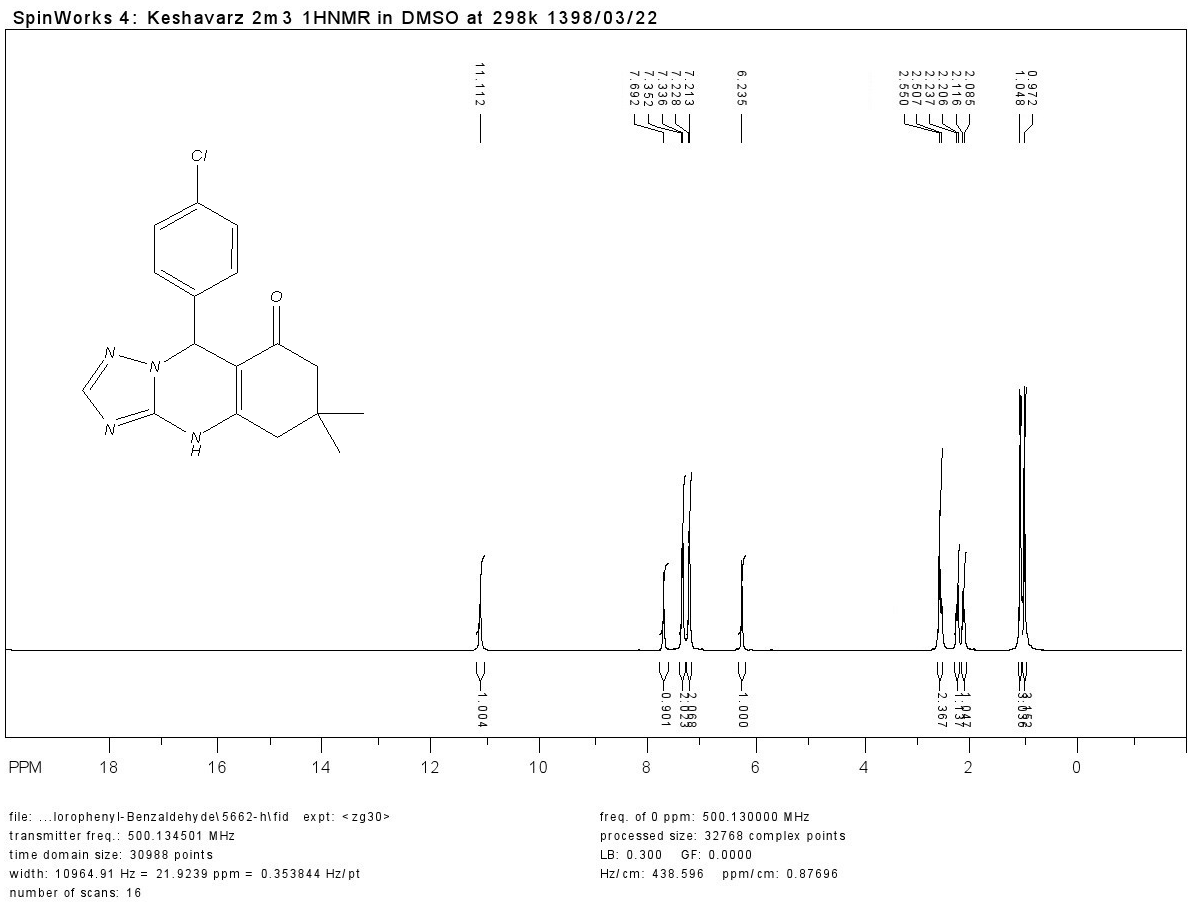


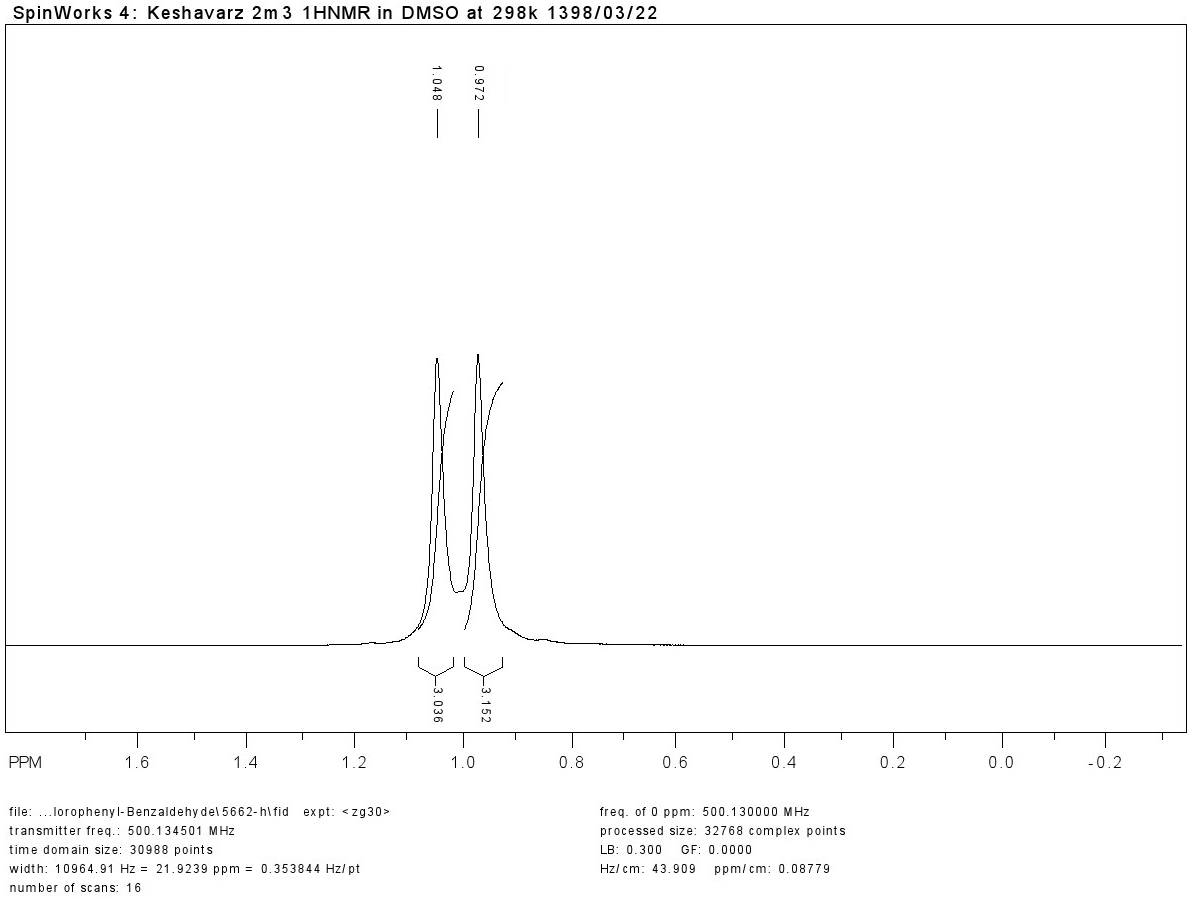


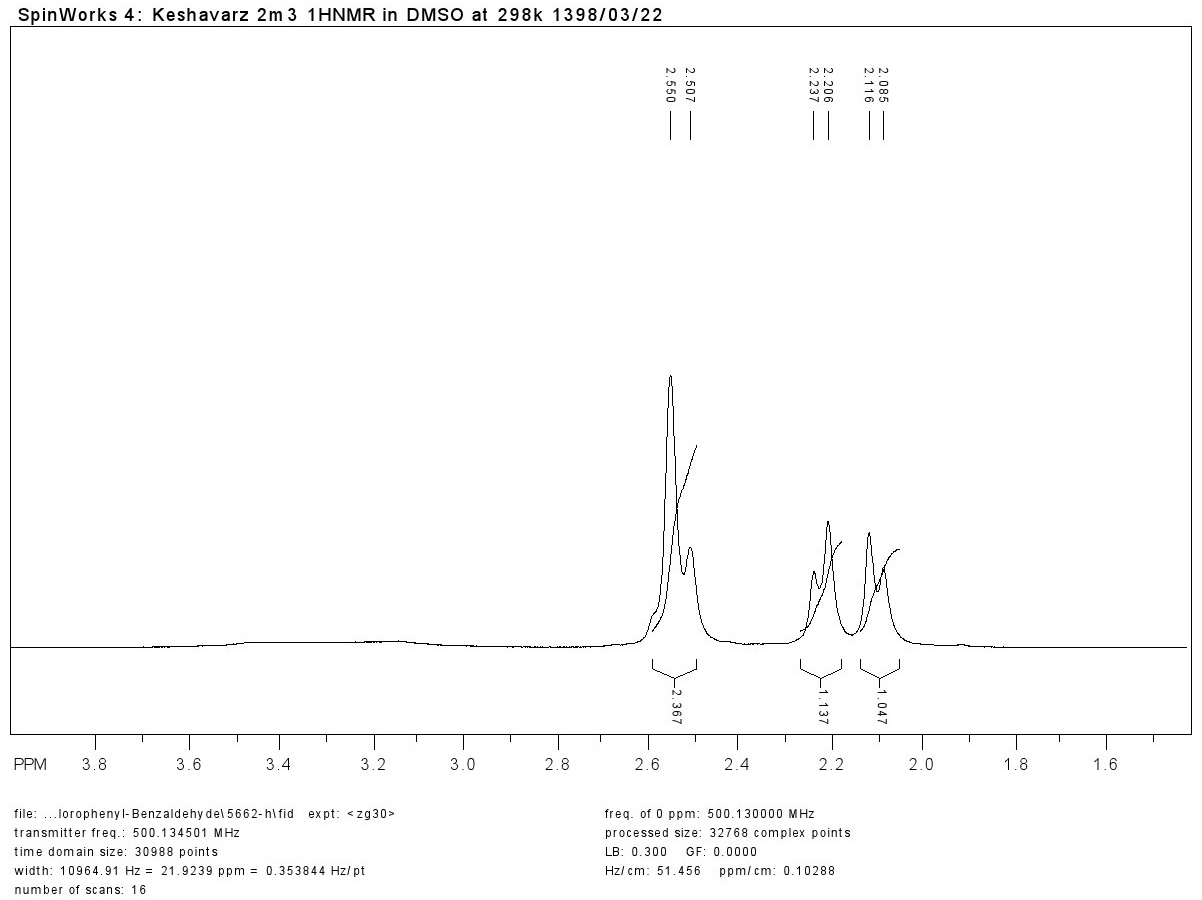


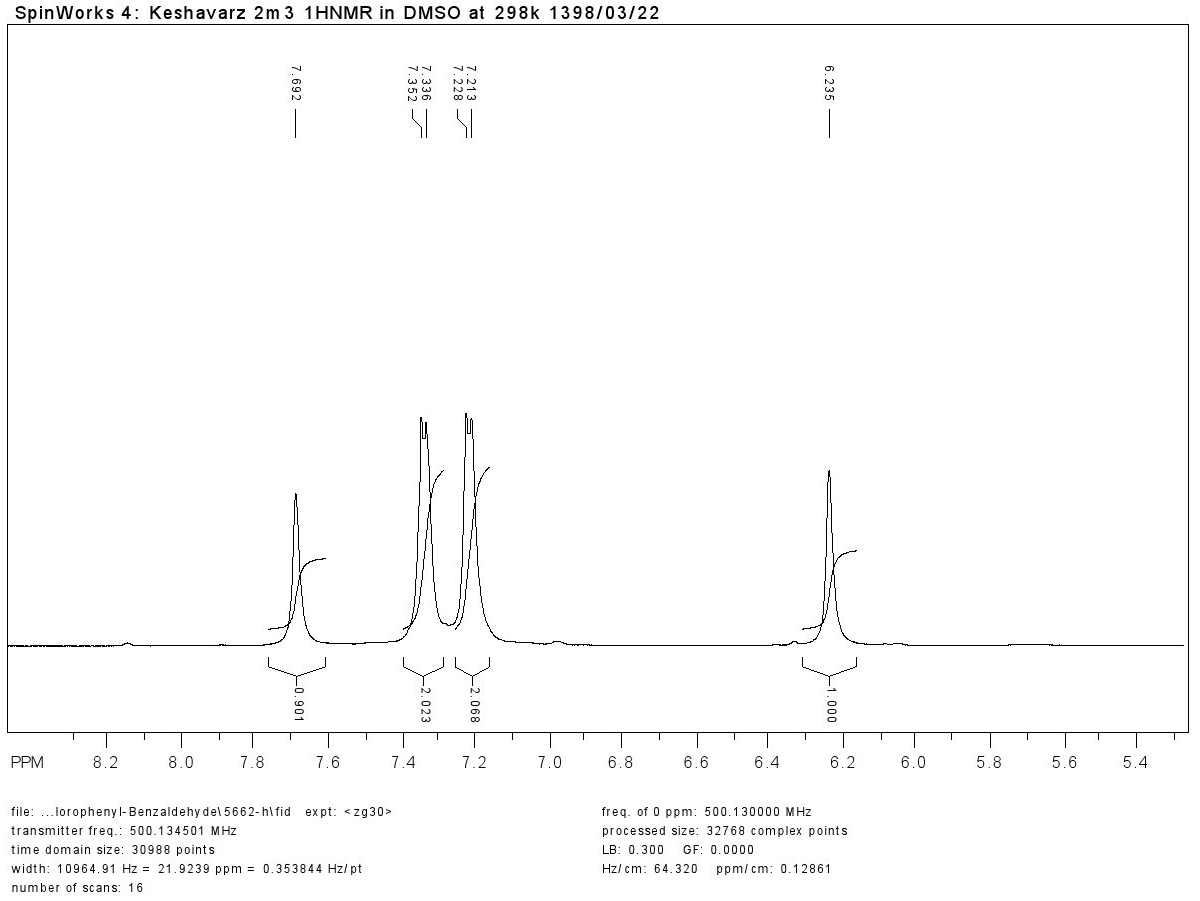


- ^13^C NMR spectrum of (**5e**, 125 MHz, DMSO-*d*_6_)


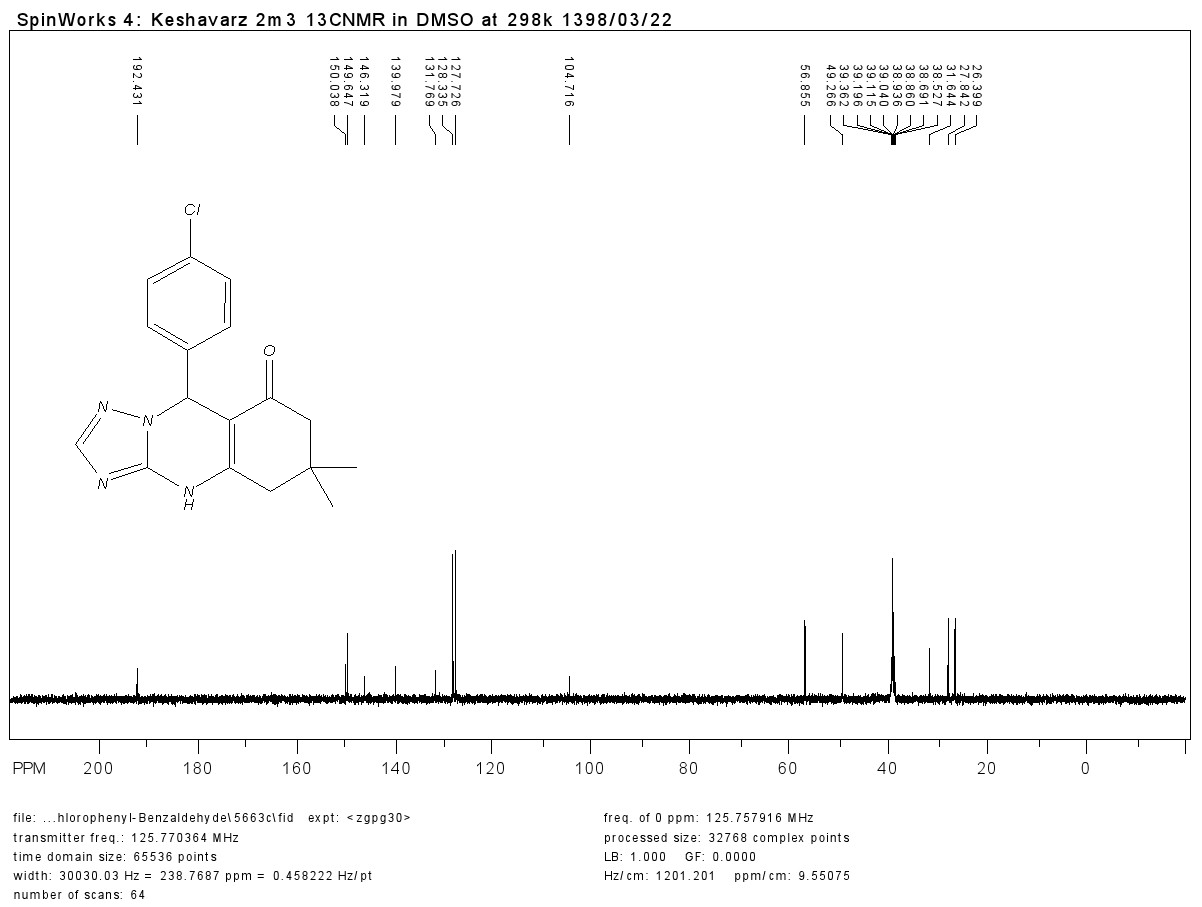


**(5f)**

- FTIR spectrum of (**5f**)


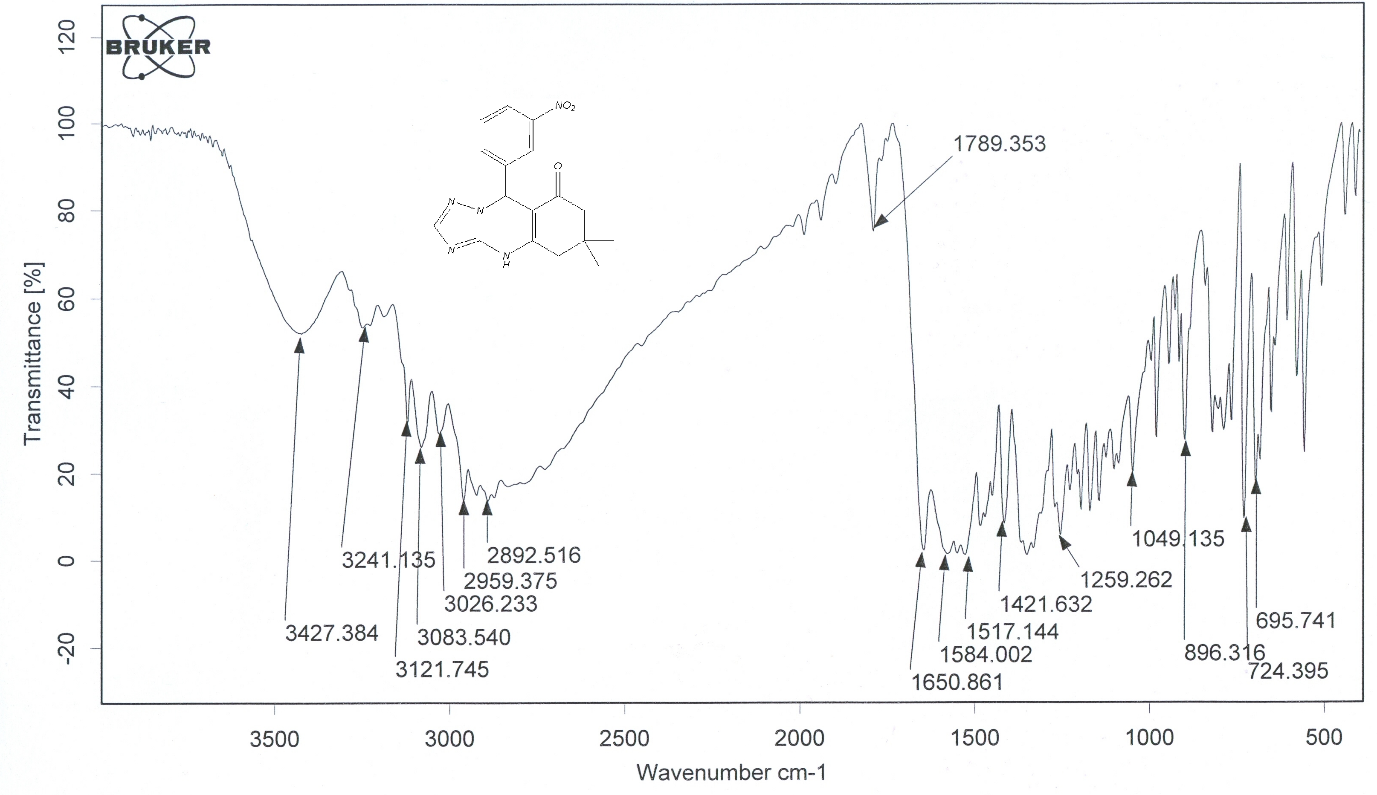


- ^1^H NMR spectrum of (**5f**, 500 MHz, DMSO-*d_6_*)


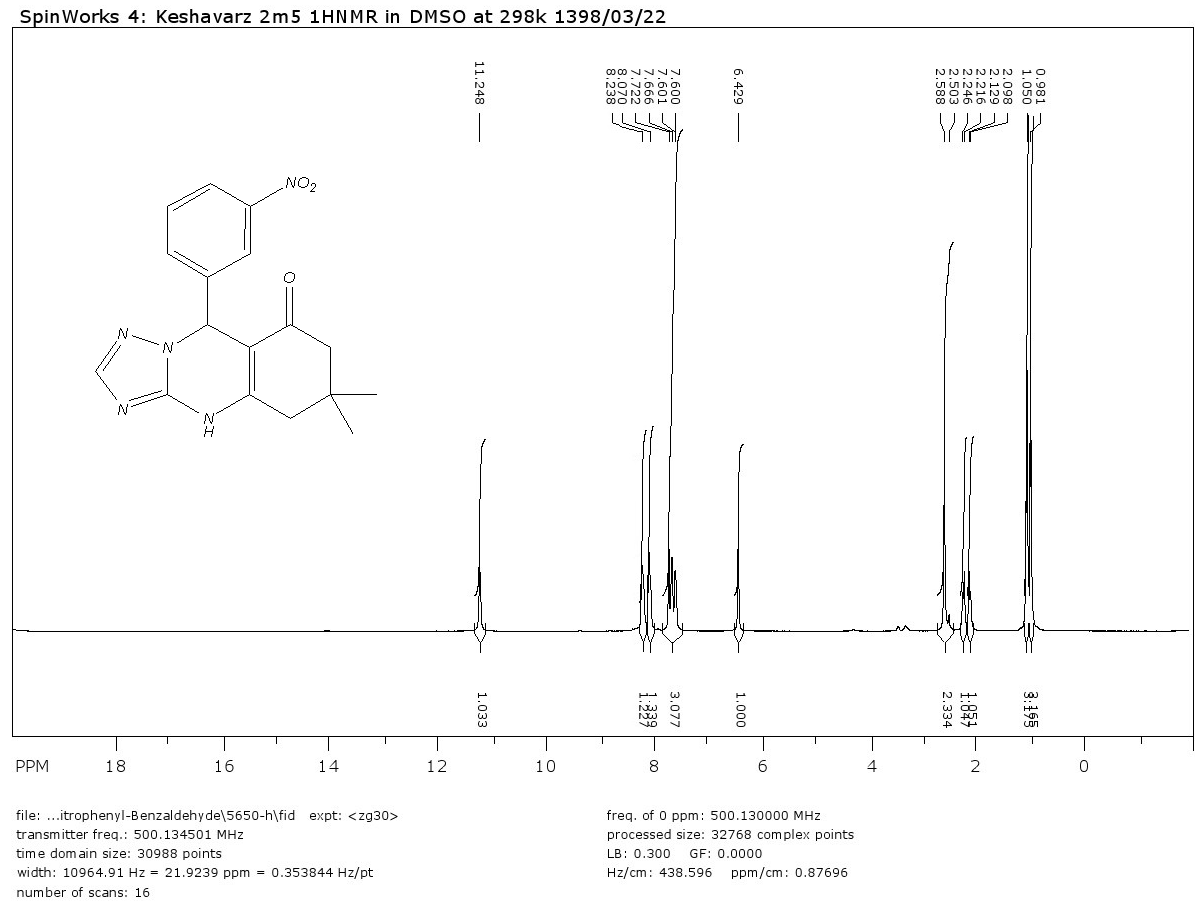


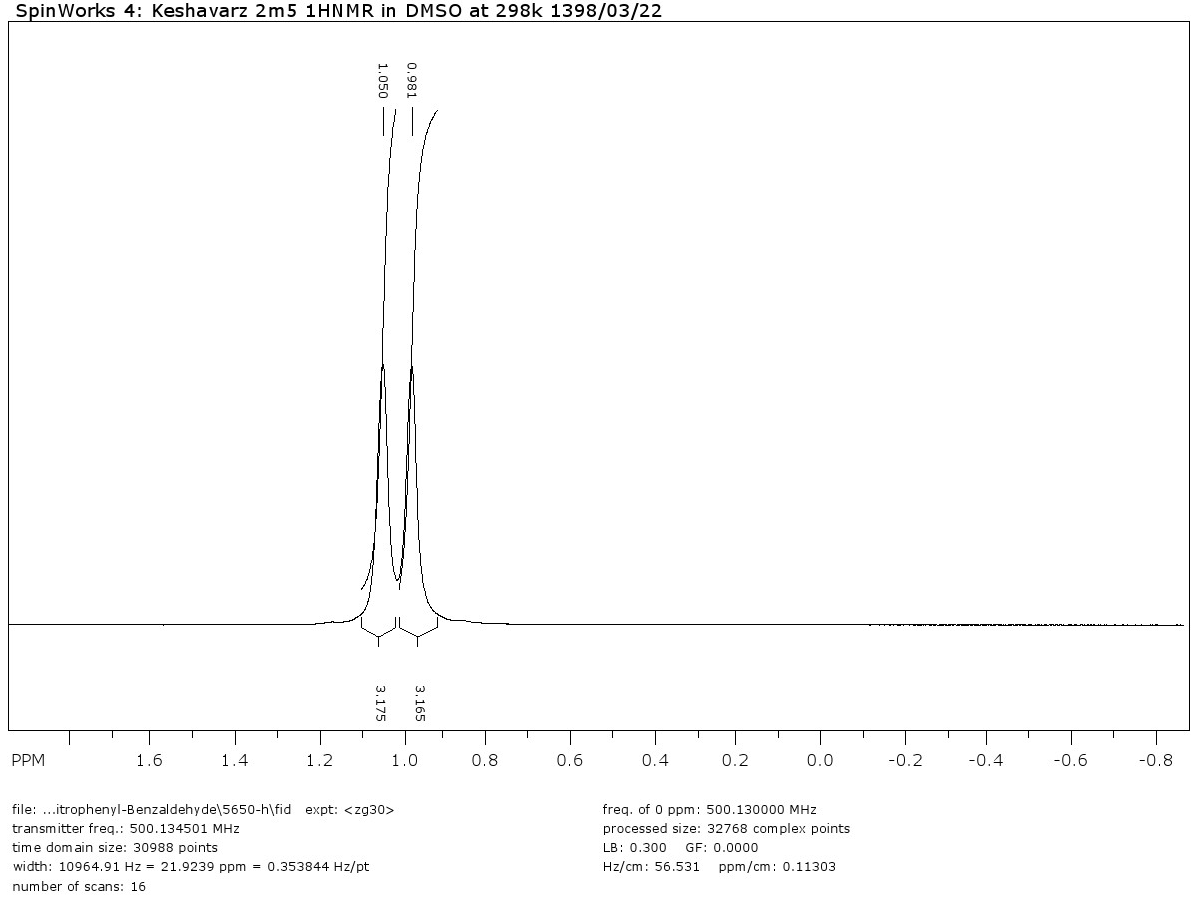


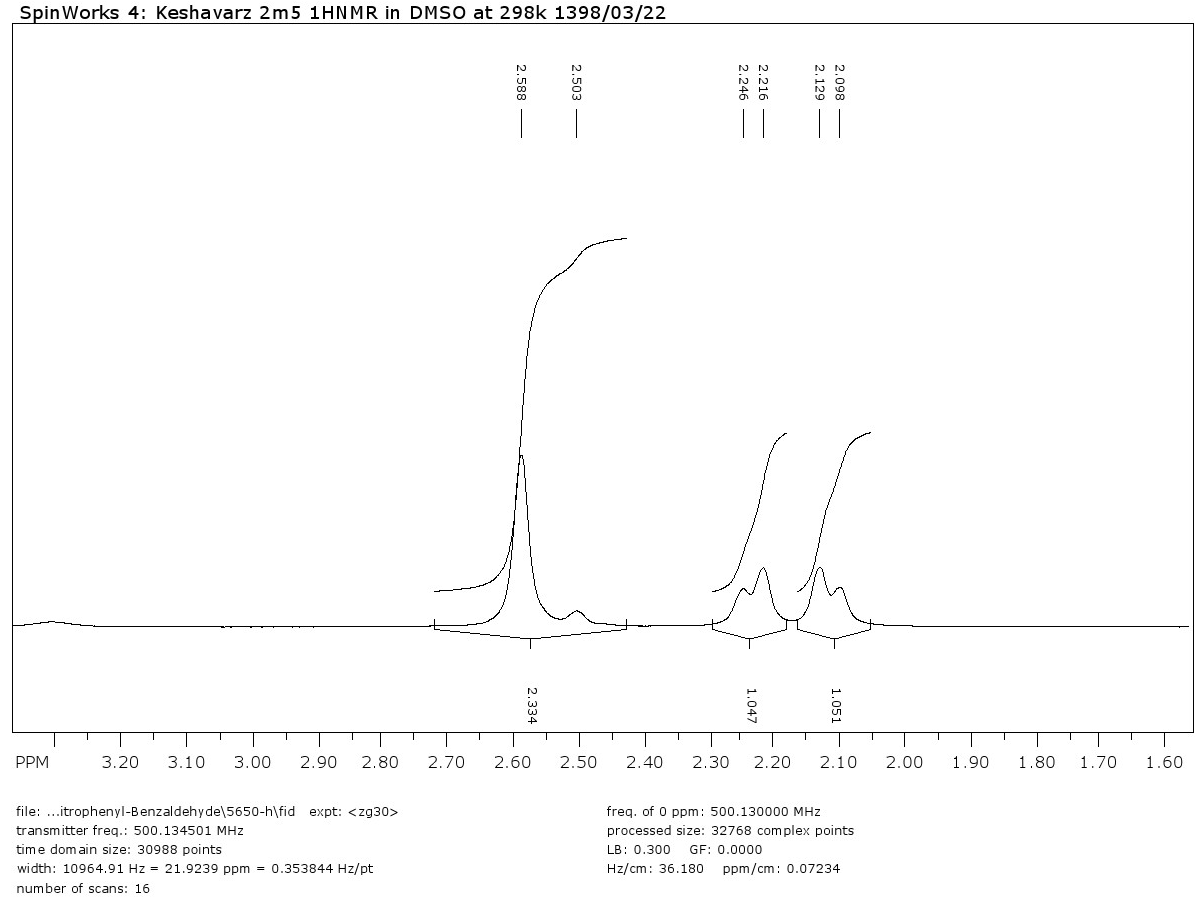


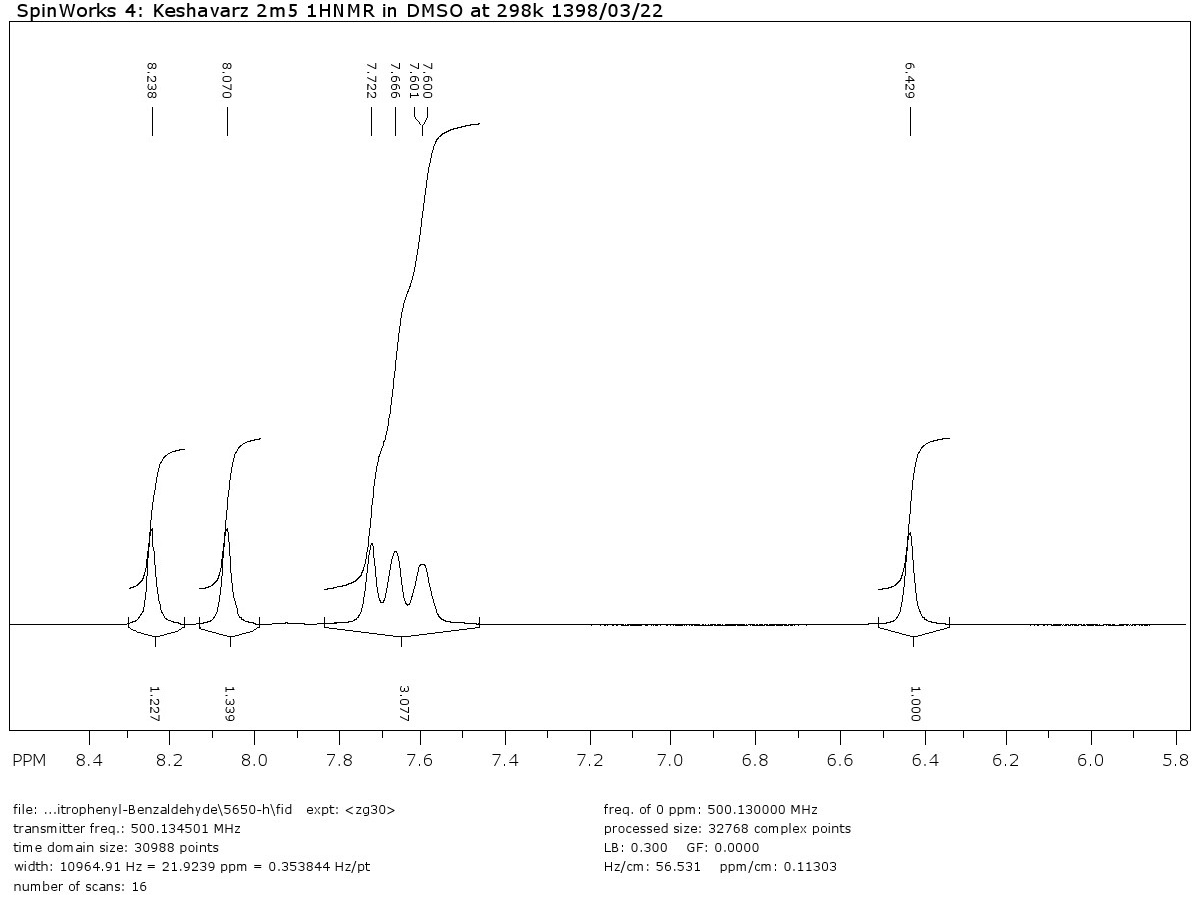


- ^13^C NMR spectrum of (**5f**, 125 MHz, DMSO-*d*_6_)


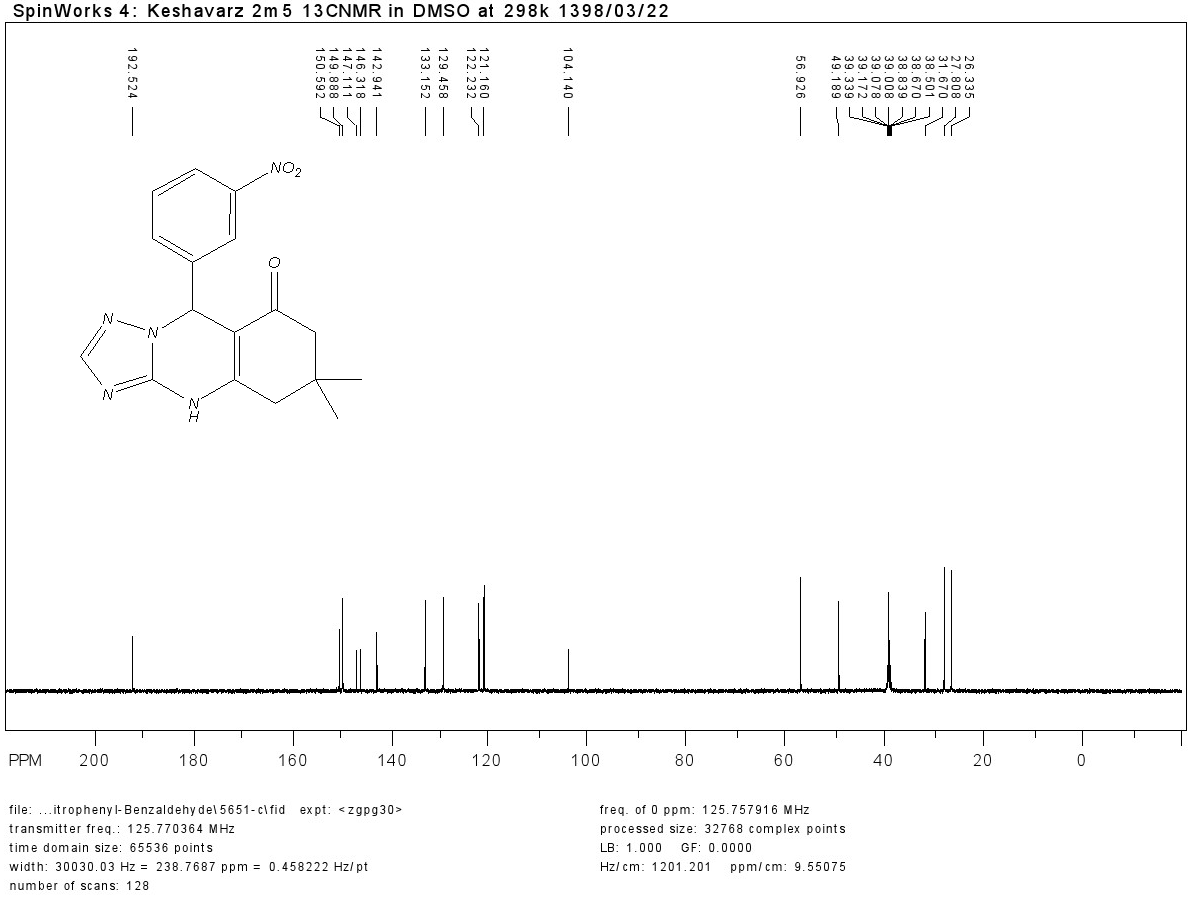


**(5g)**

- FTIR spectrum of (**5g**)


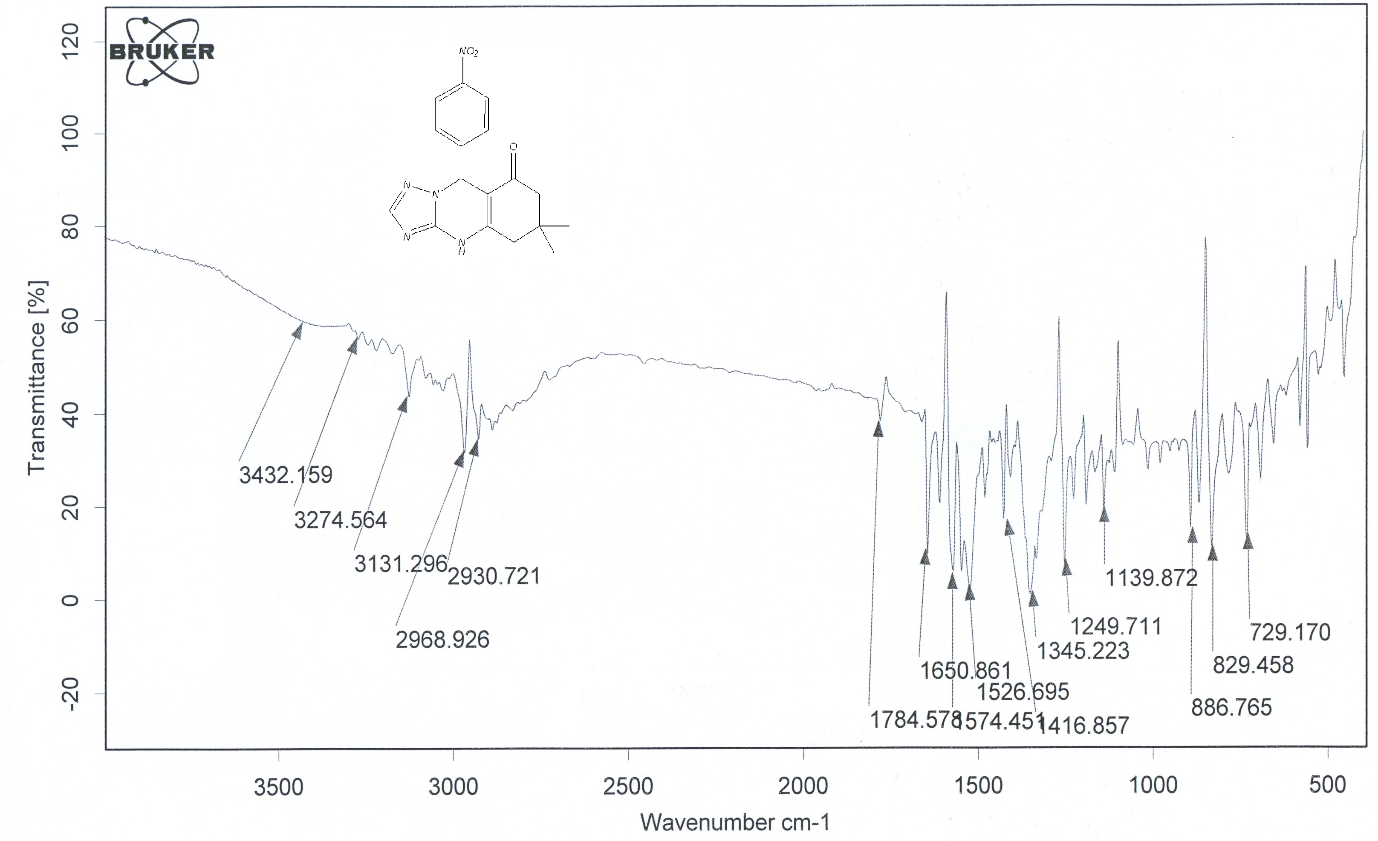


- ^1^H NMR spectrum of (**5g**, 500 MHz, DMSO-*d_6_*)


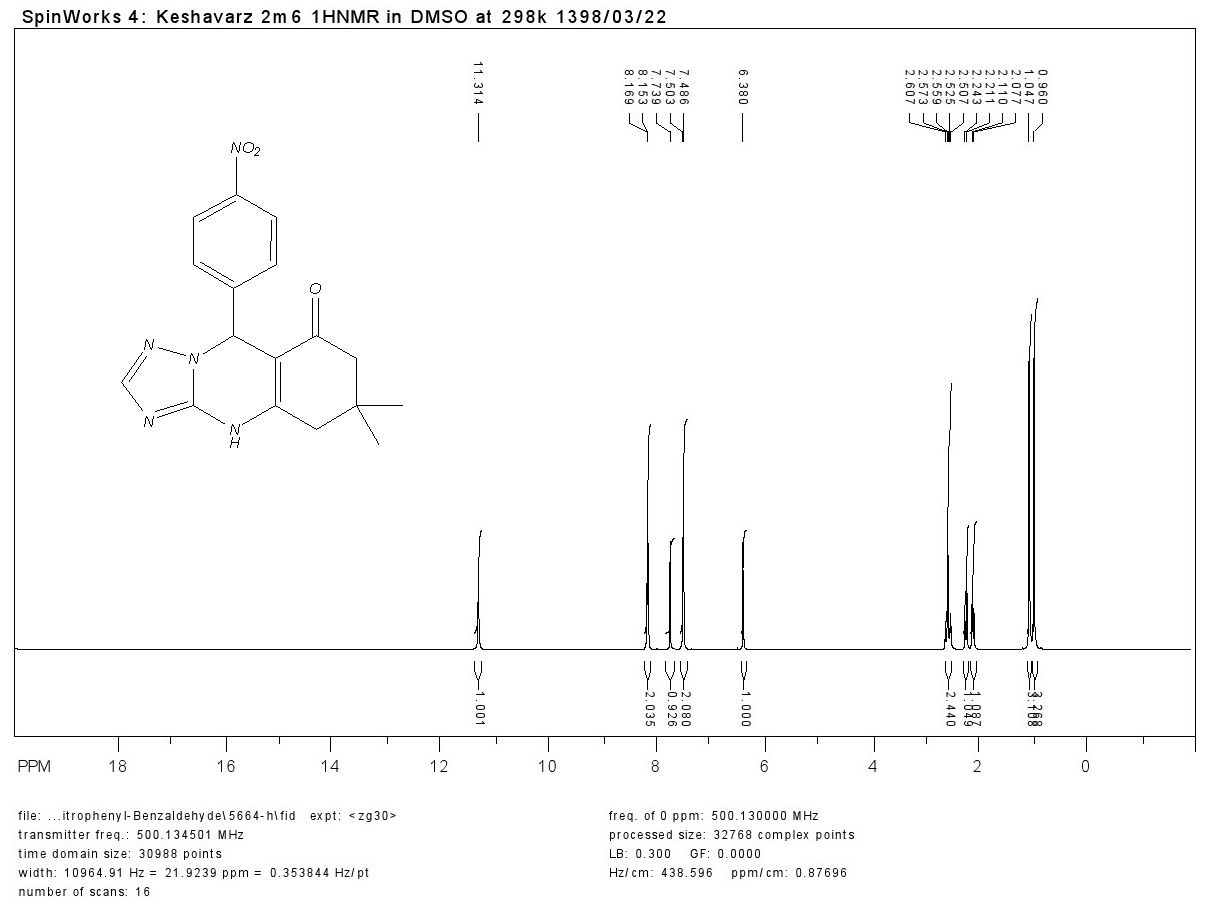
ا


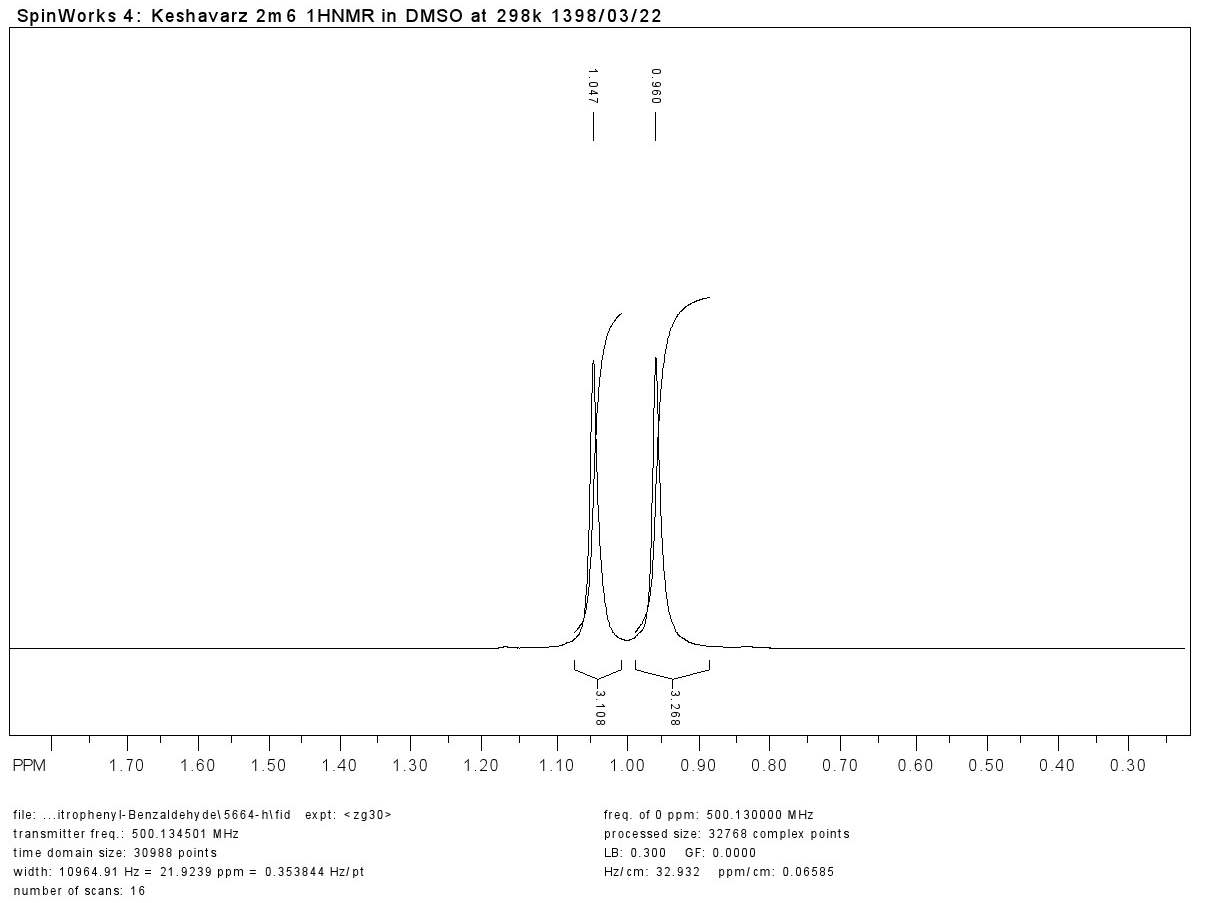


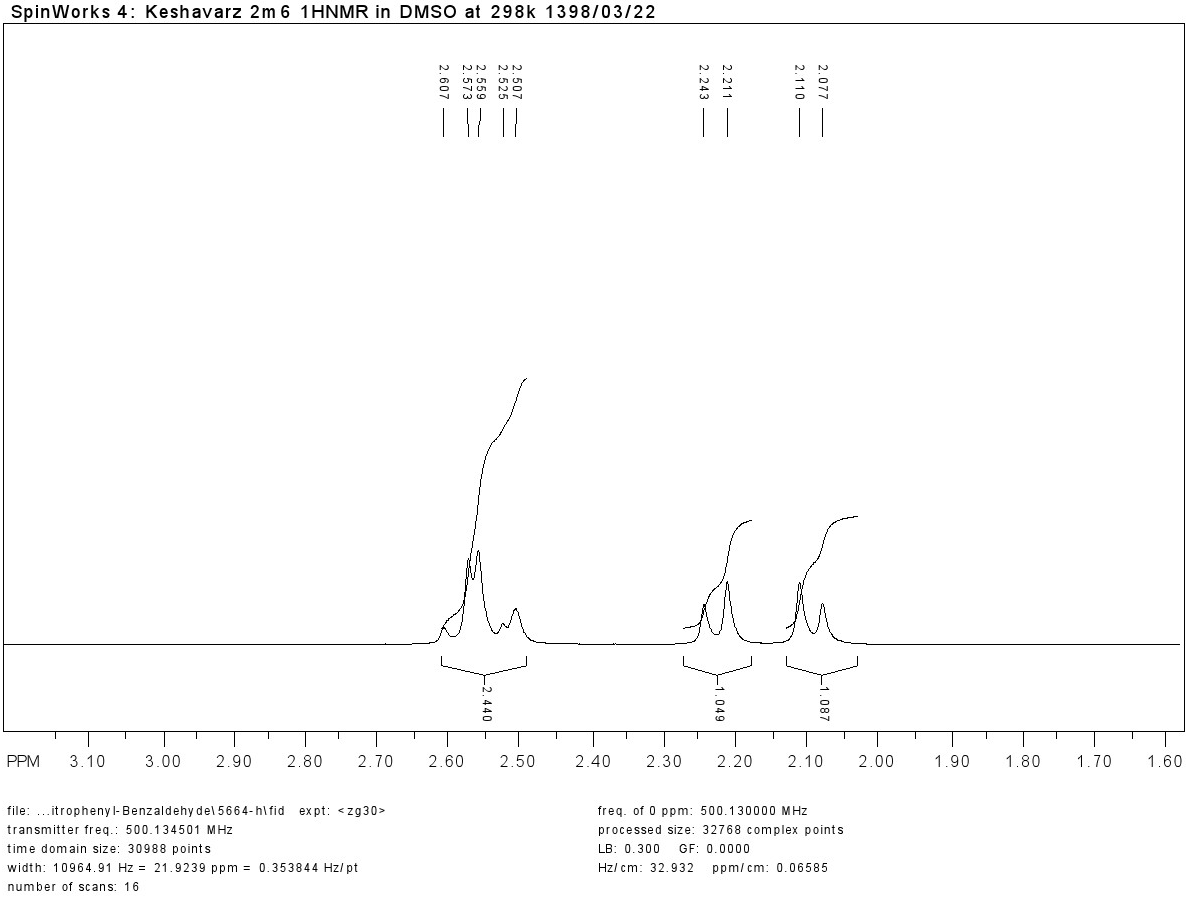


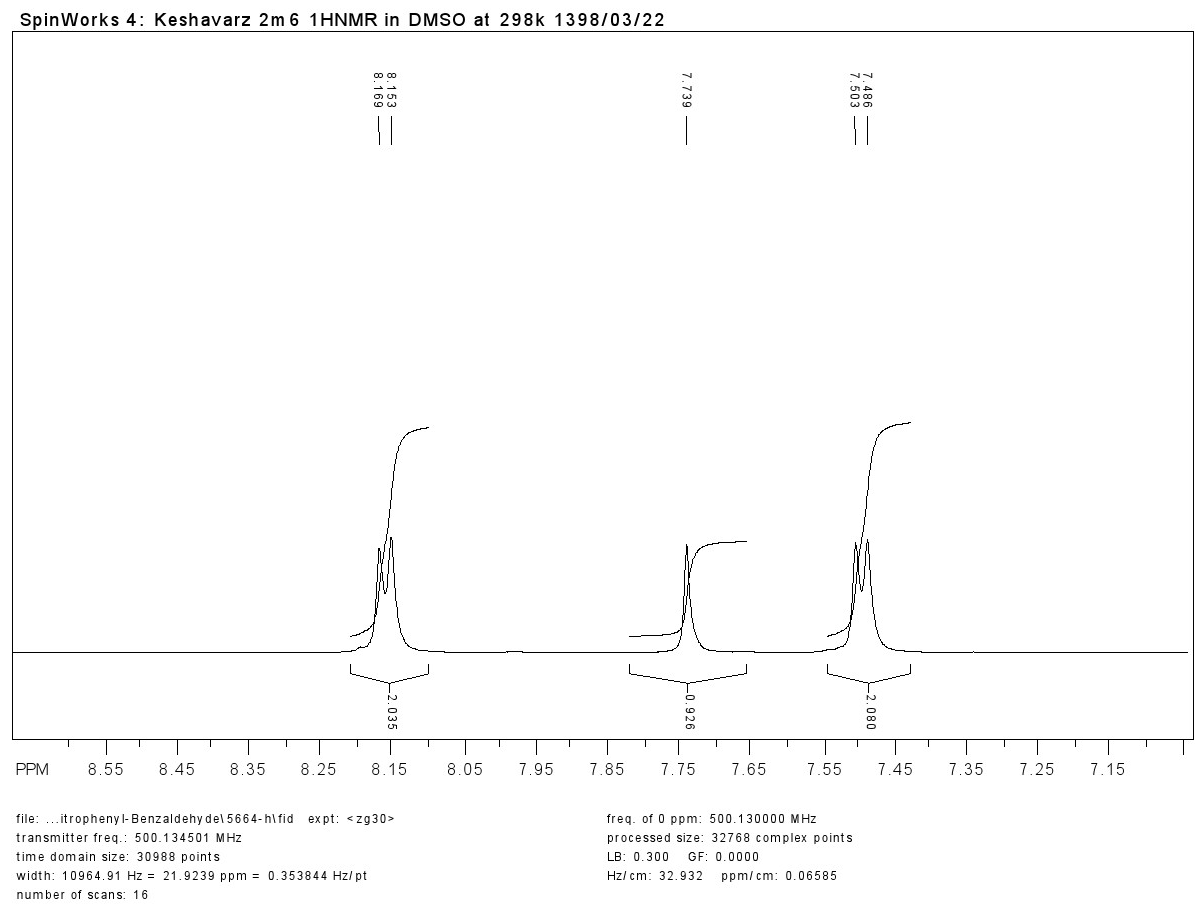


- ^13^C NMR spectrum of (**5g**, 125 MHz, DMSO-*d*_6_)


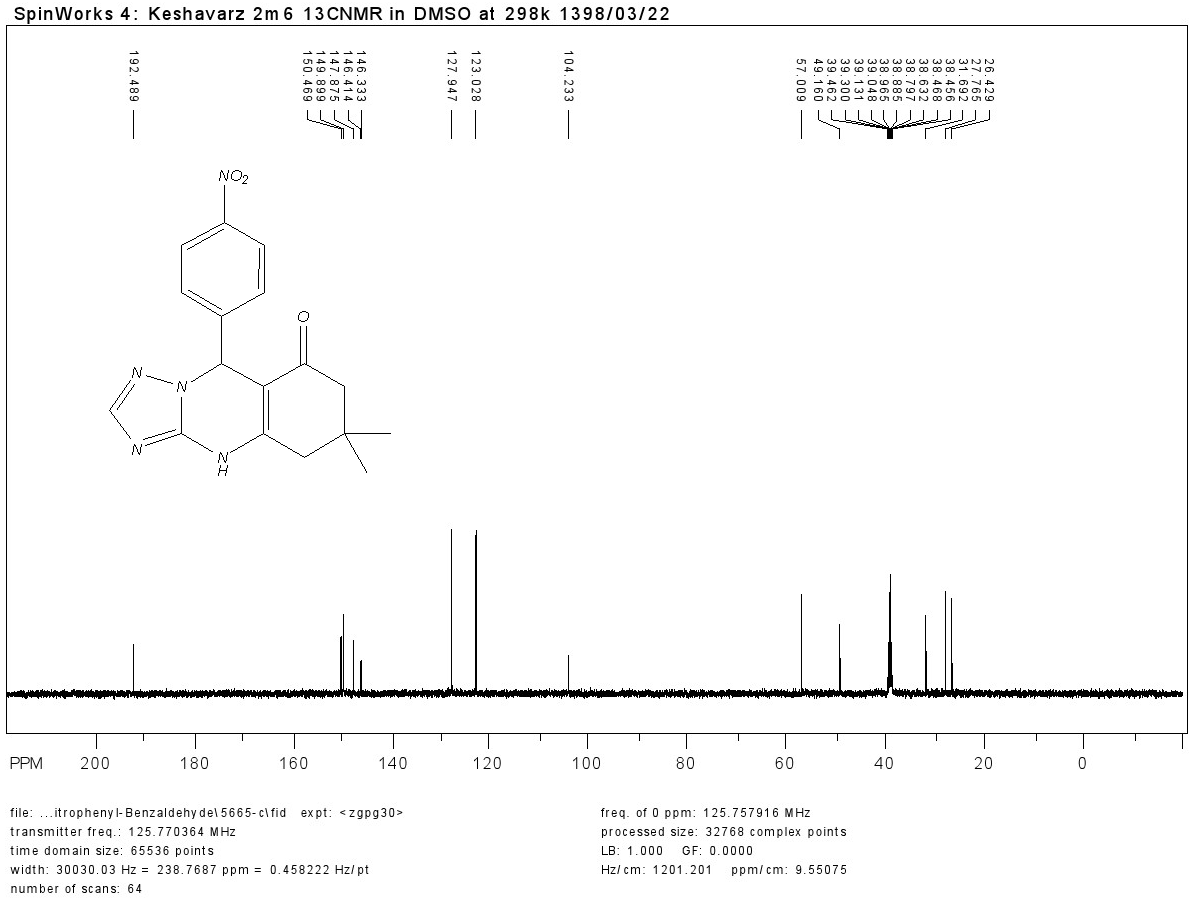


**(5h)**

- FTIR spectrum of (**5h**)


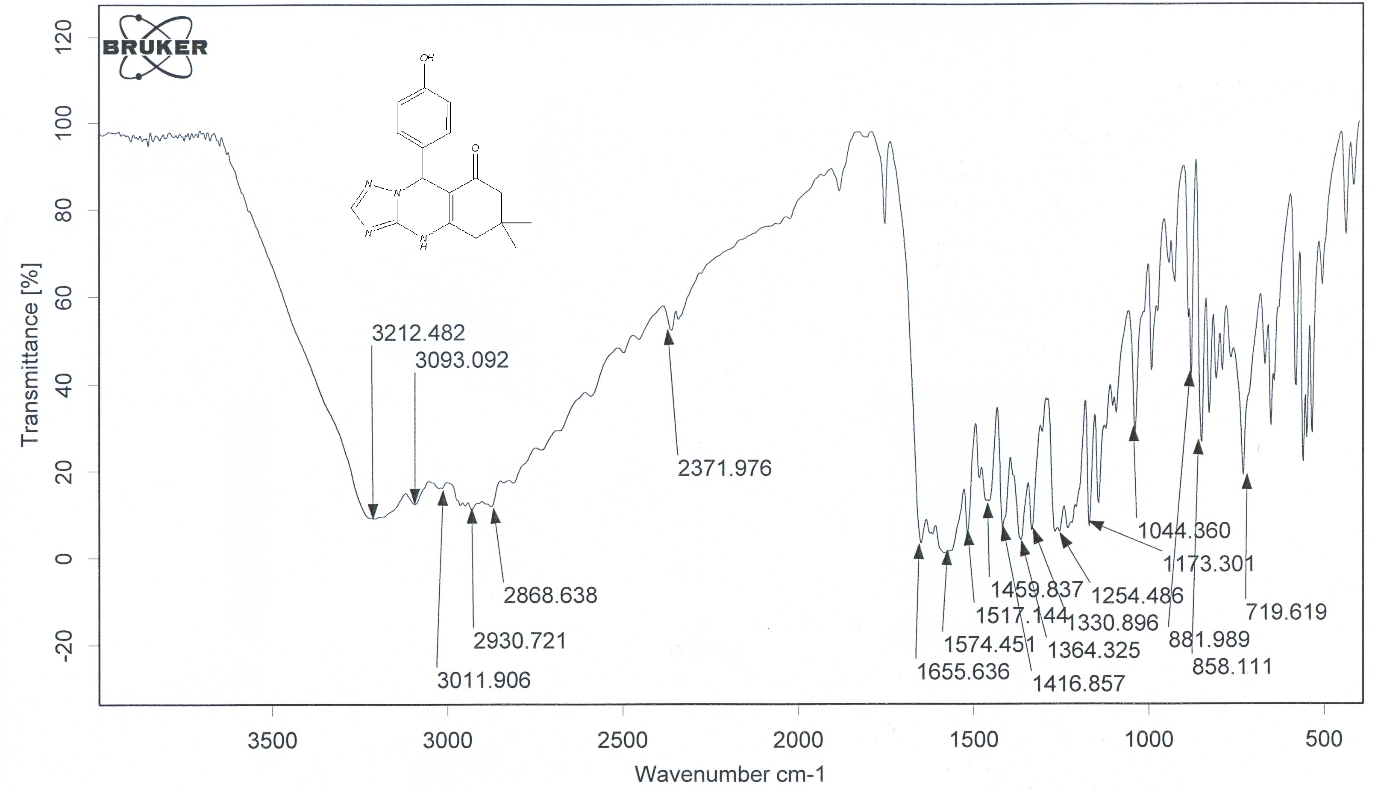


- ^1^H NMR spectrum of (**5h**, 500 MHz, DMSO-*d_6_*)


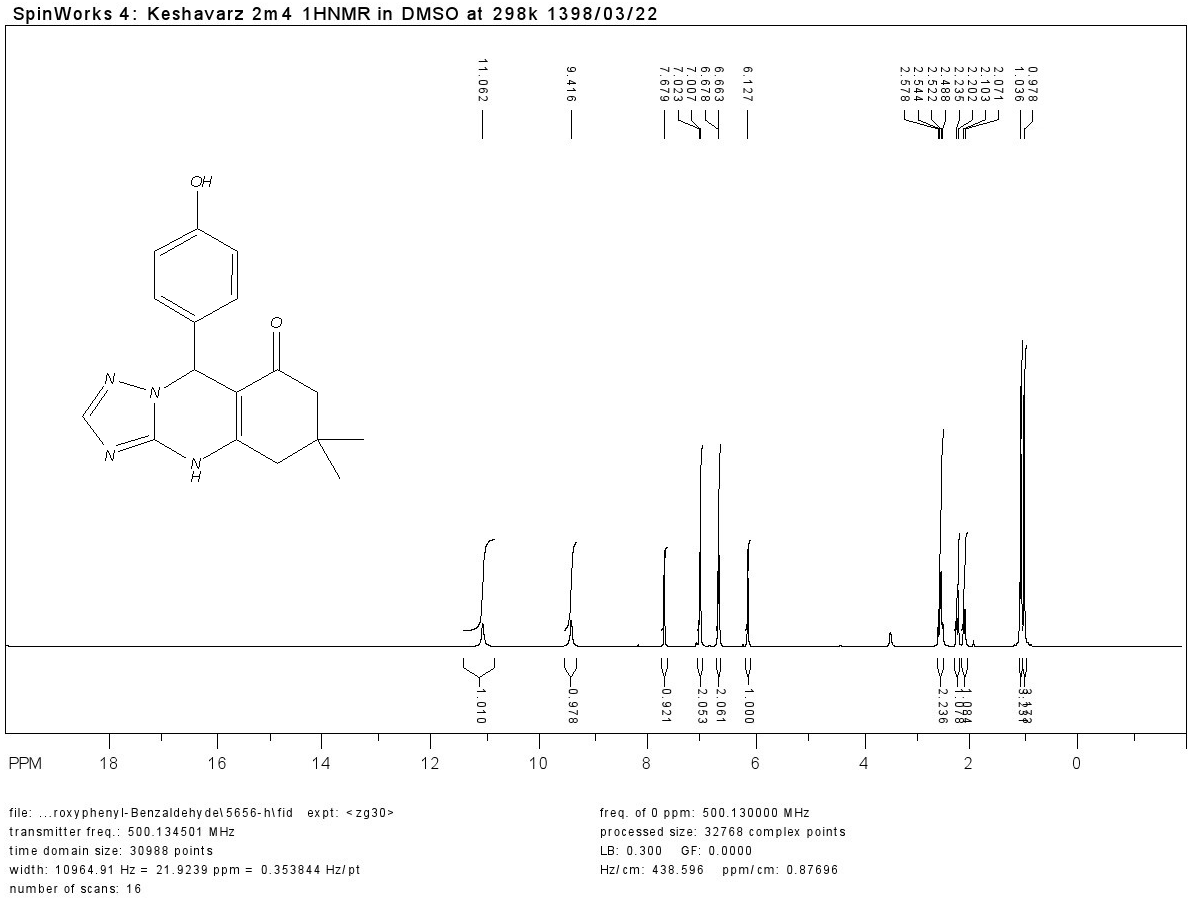


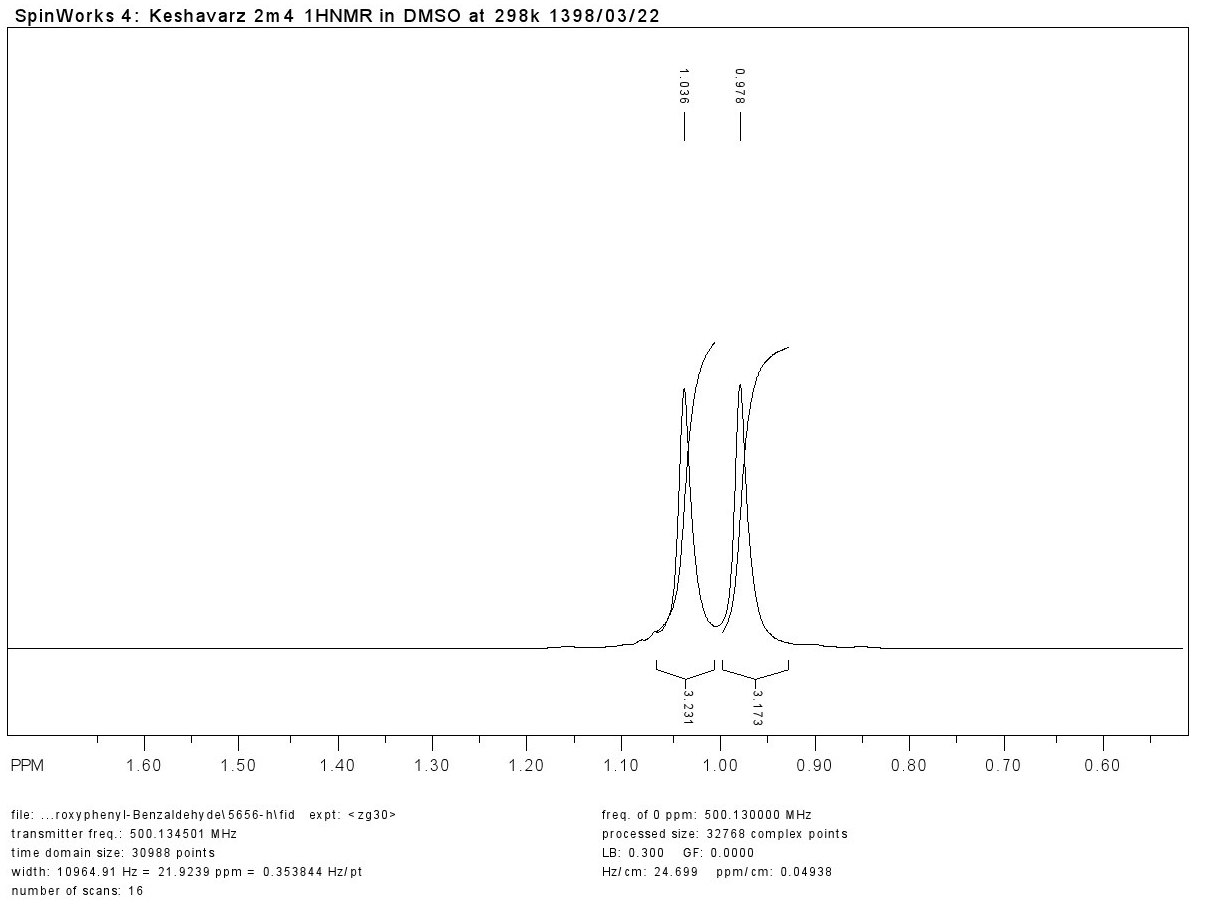


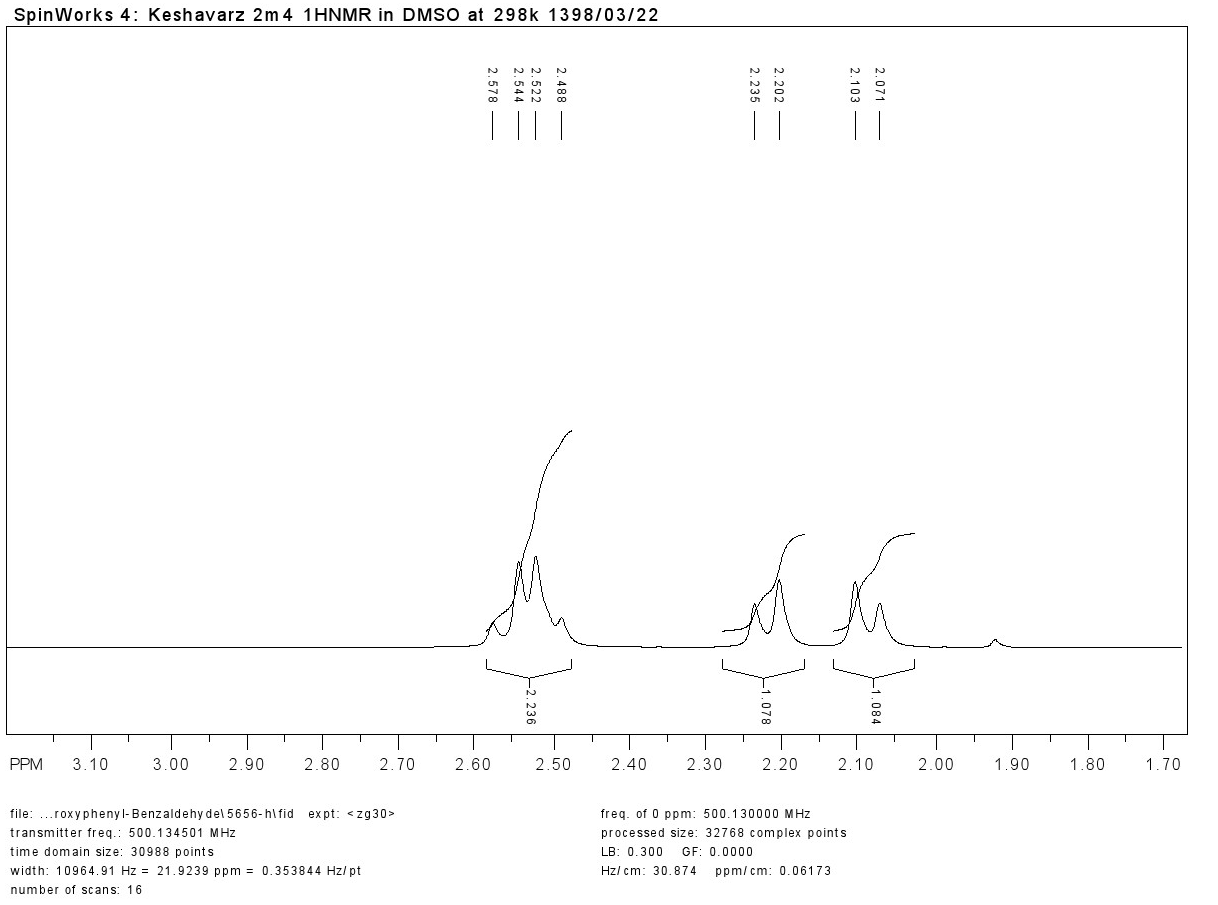


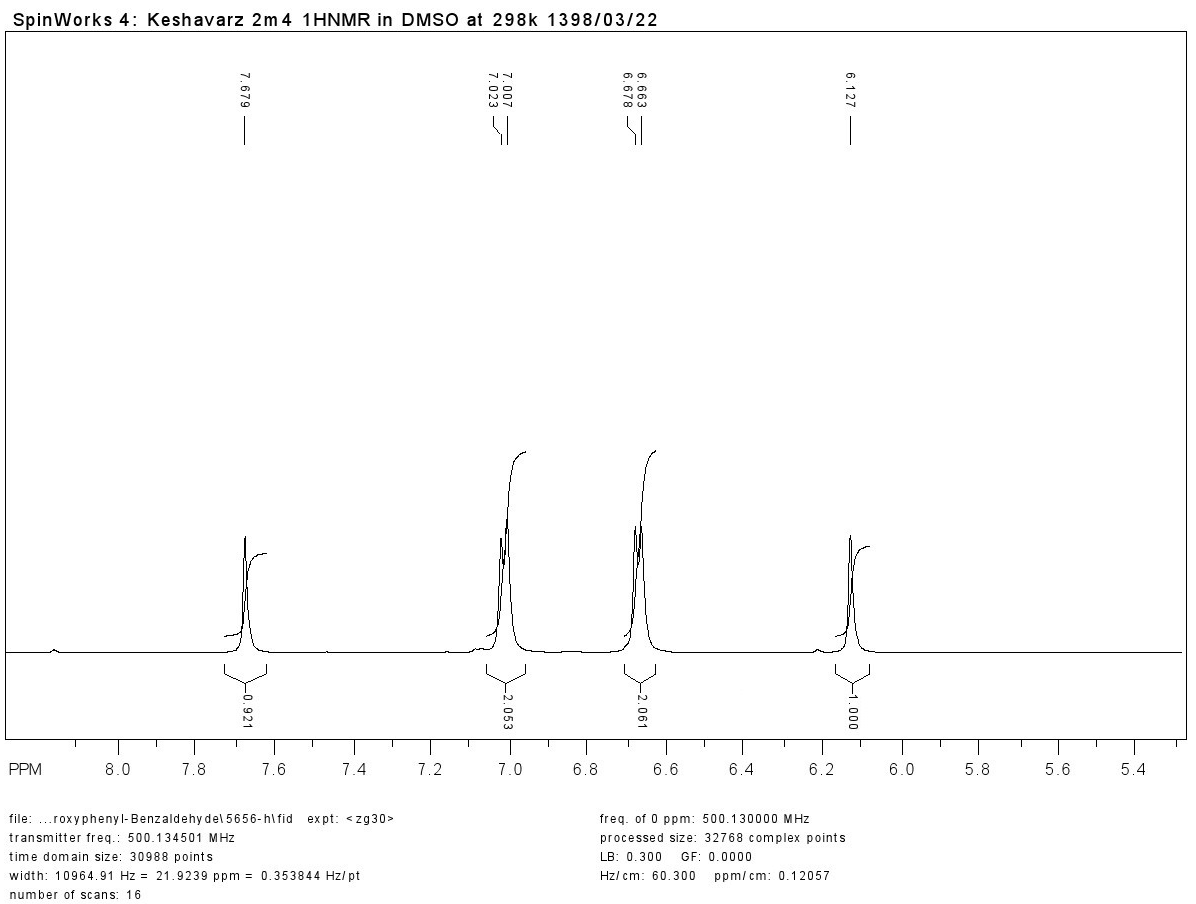


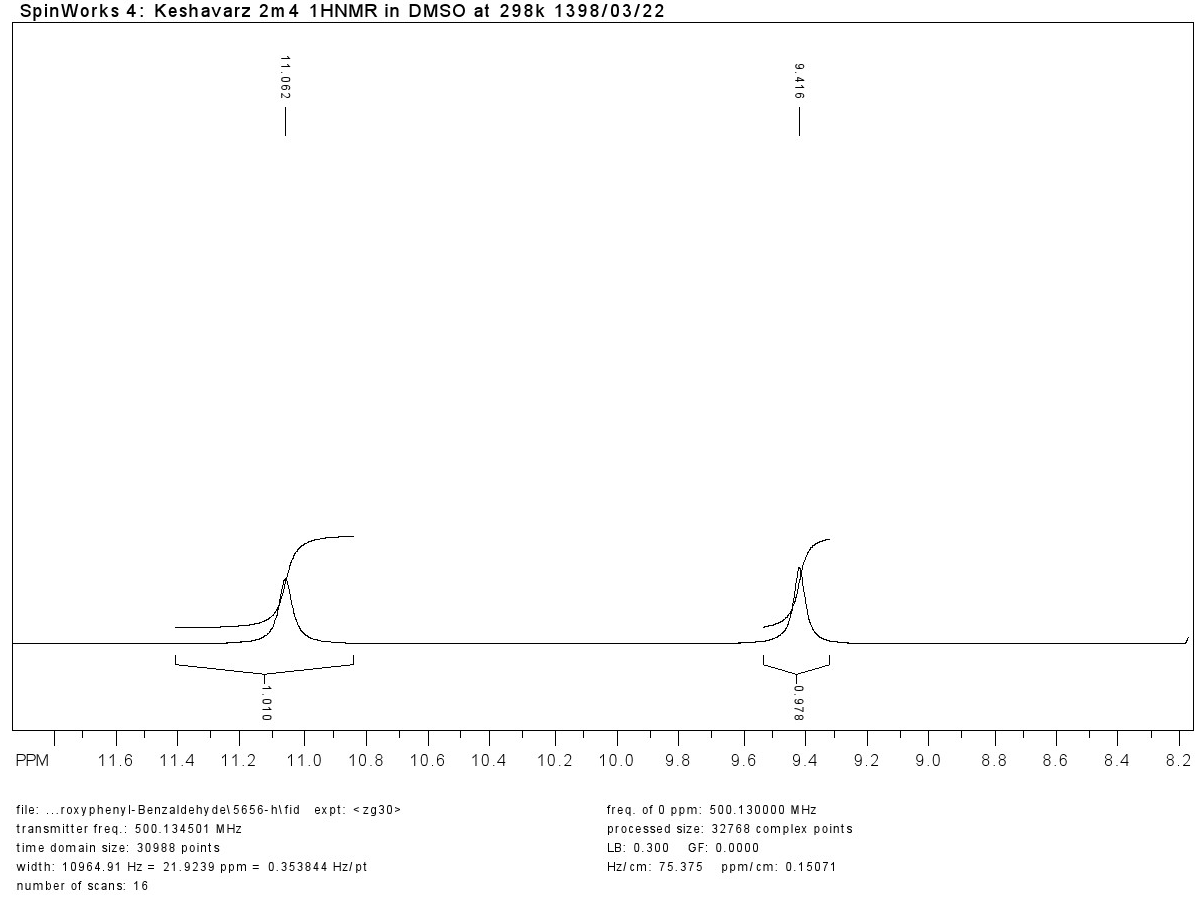


- ^13^C NMR spectrum of (**5h**, 125 MHz, DMSO-*d*_6_)


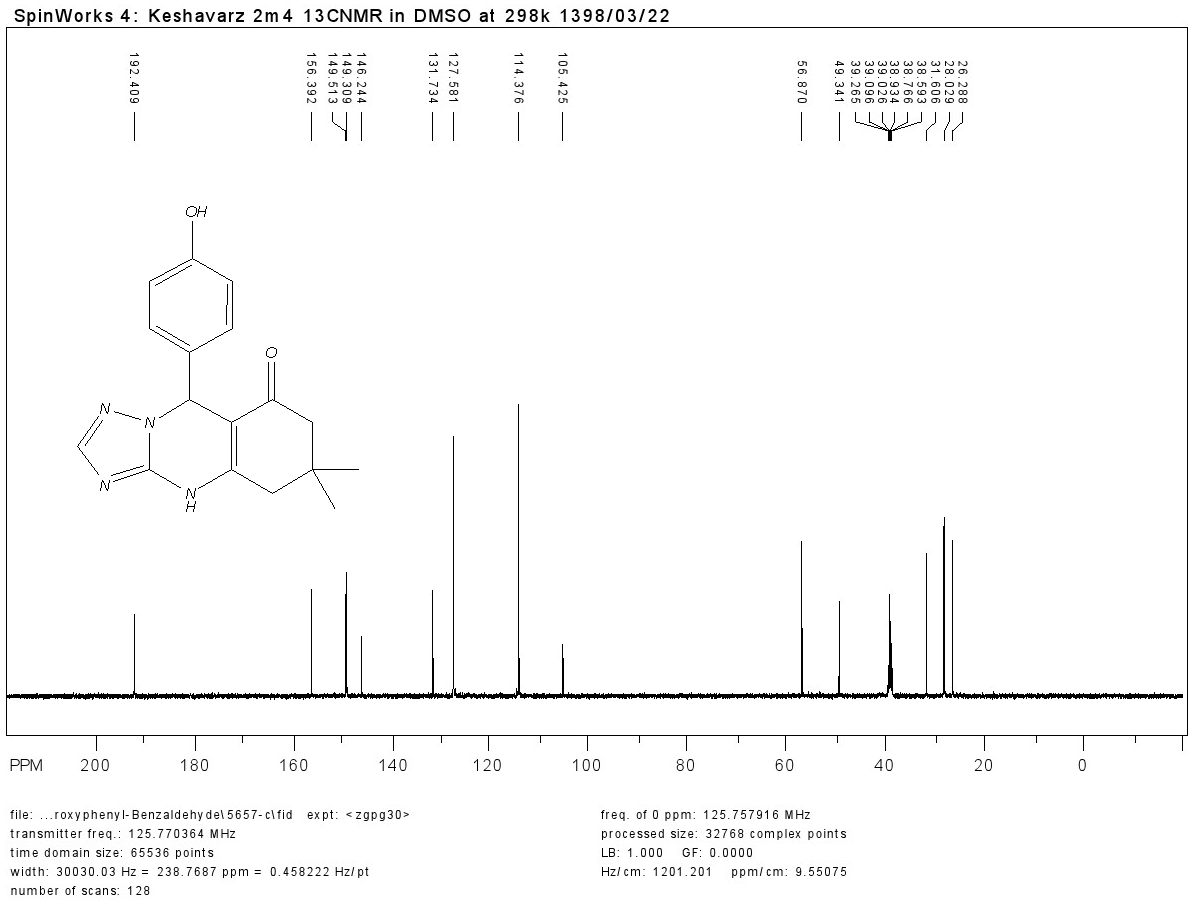


**(5i)**

- FTIR spectrum of (**5i**)


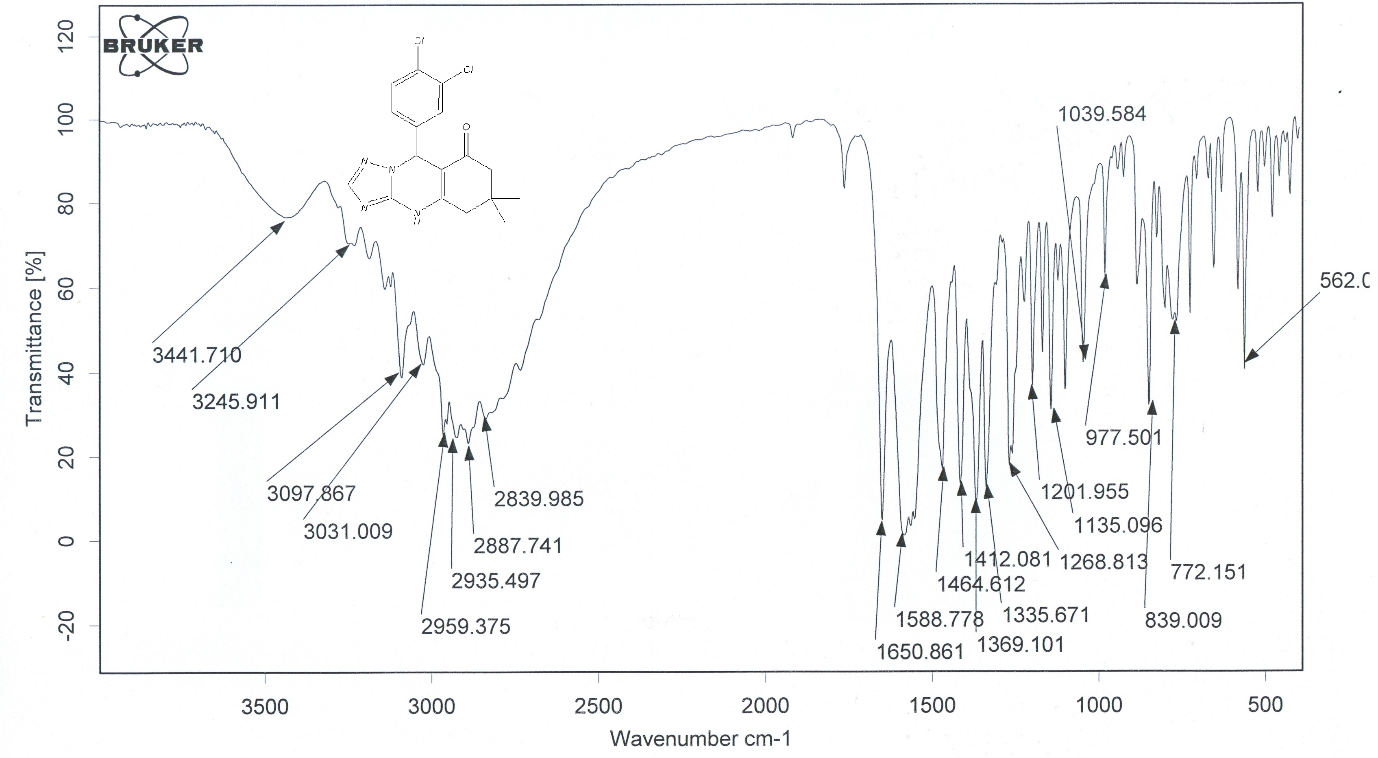


- ^1^H NMR spectrum of (**5i**, 500 MHz, DMSO-*d_6_*)


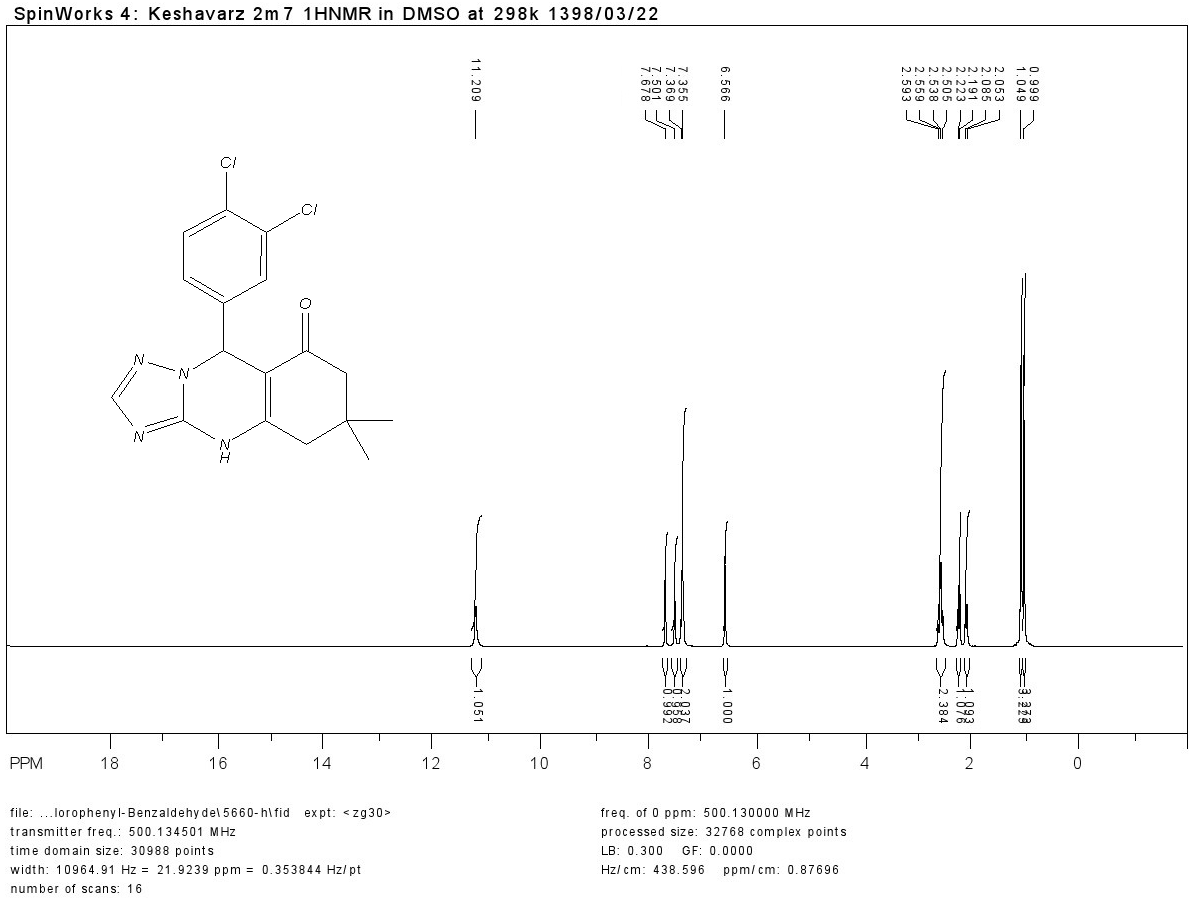


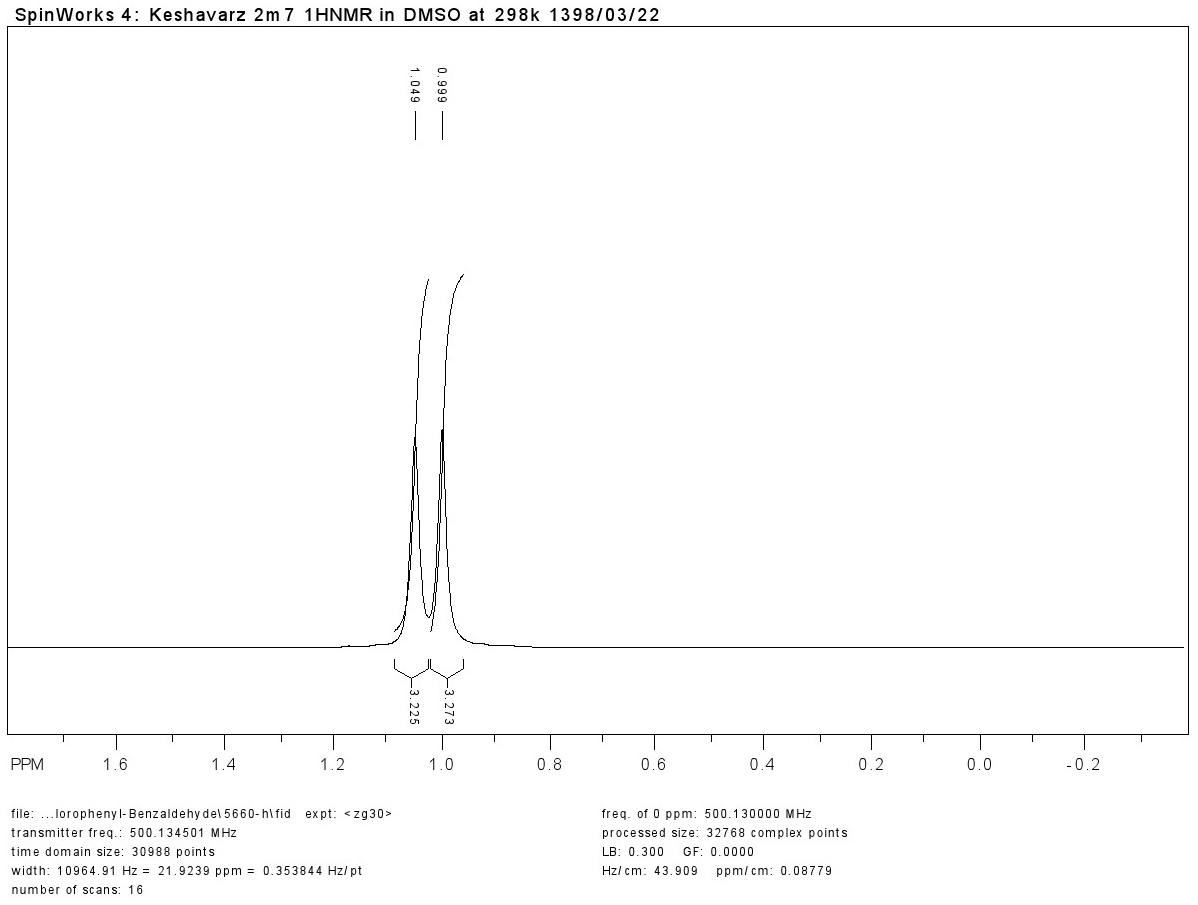


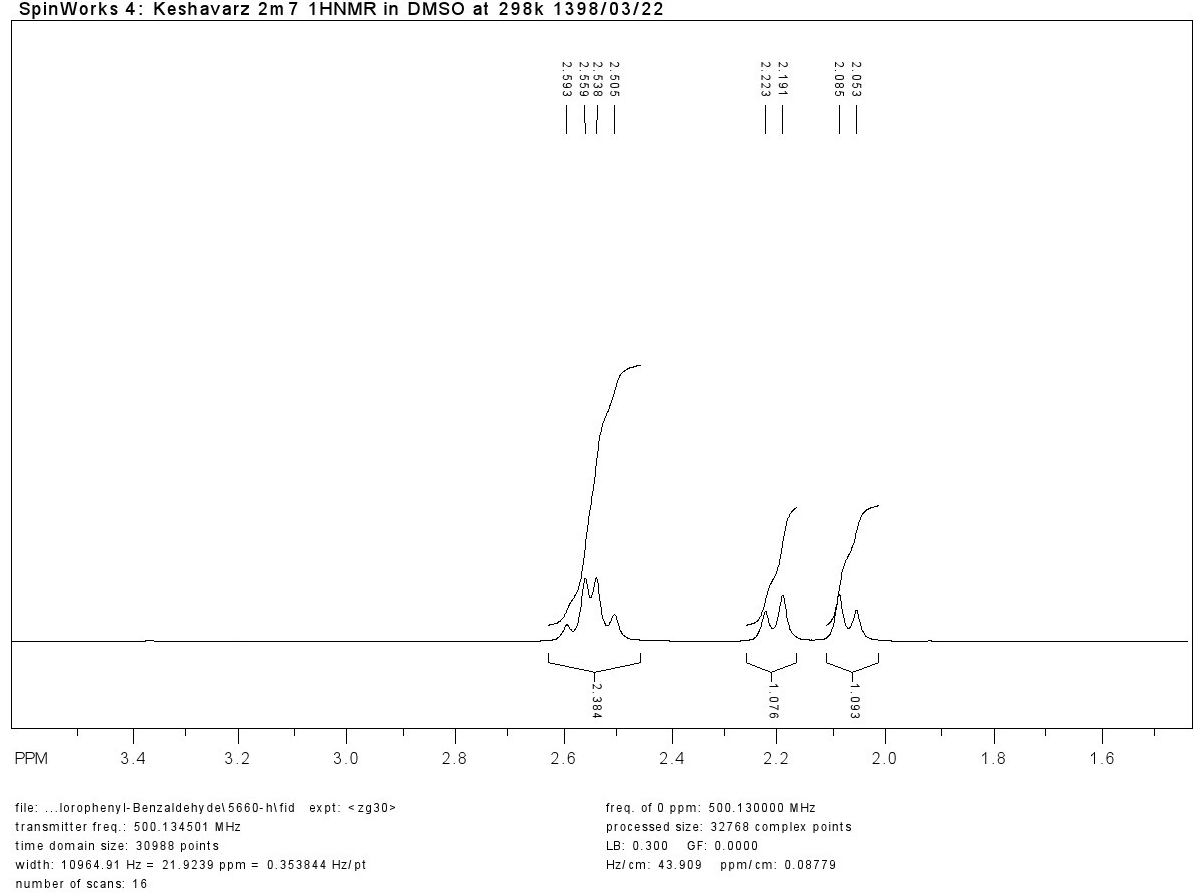


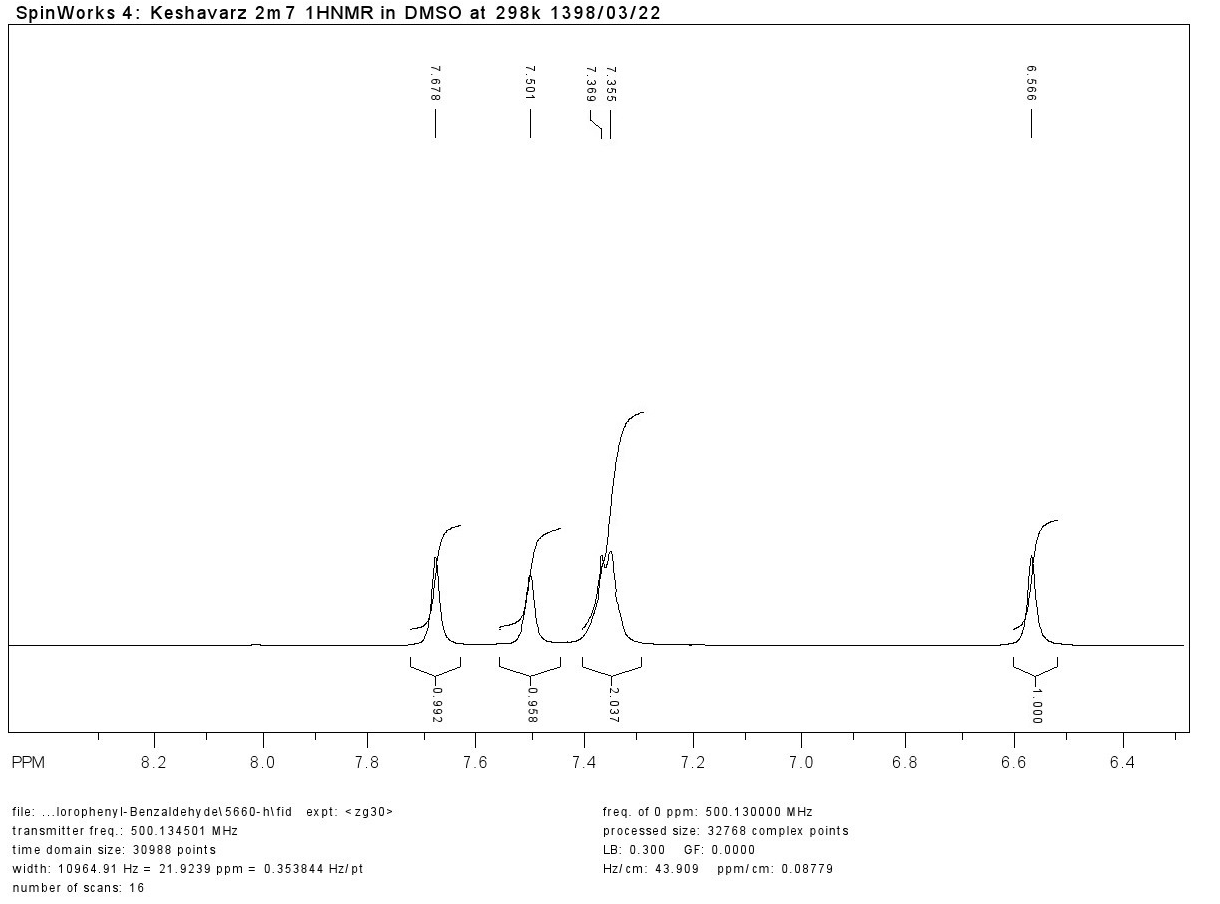


- ^13^C NMR spectrum of (**5i**, 125 MHz, DMSO-*d*_6_)


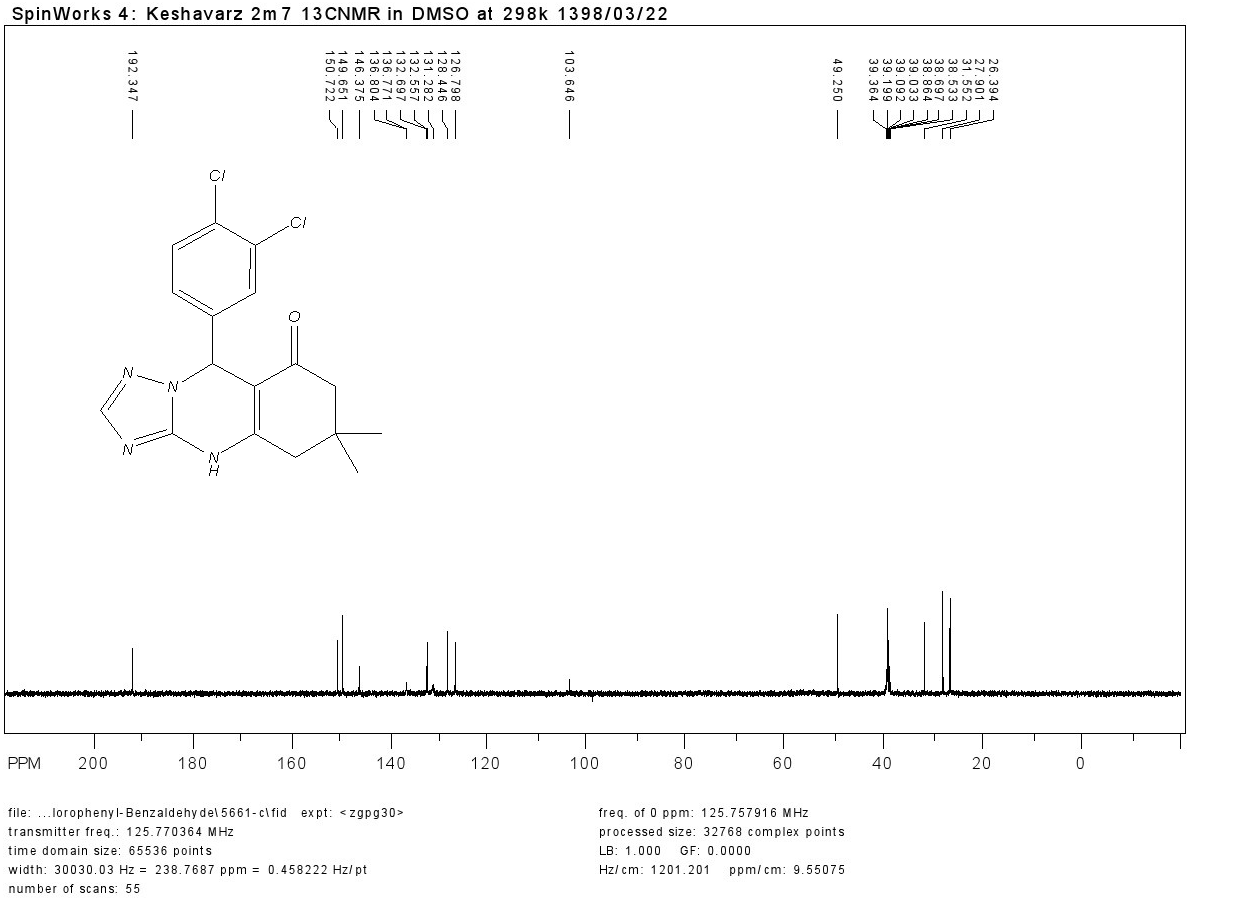


- CHN analysis of **5i**

**(5j)**

- FTIR spectrum of (**5j**)


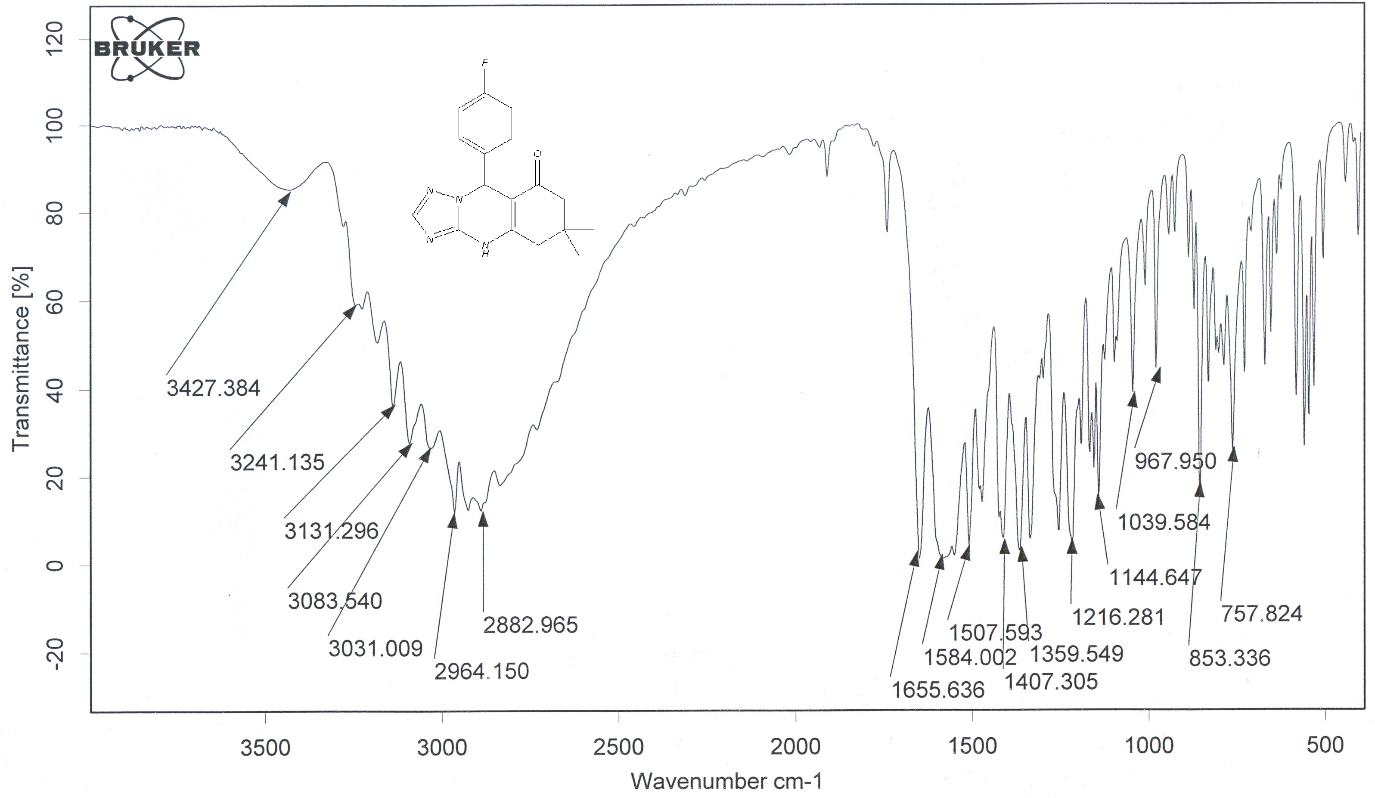


- ^1^H NMR spectrum of (**5j**, 500 MHz, DMSO-*d_6_*)


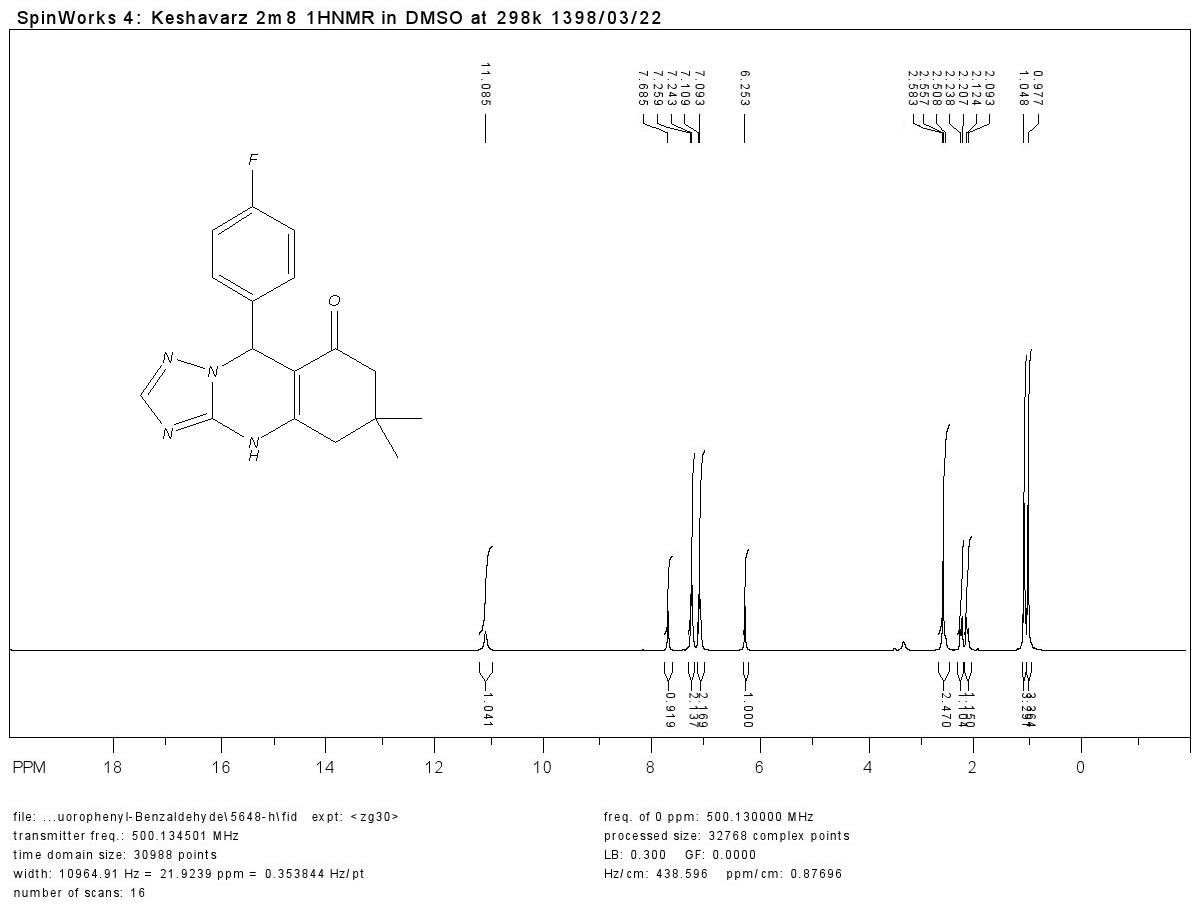


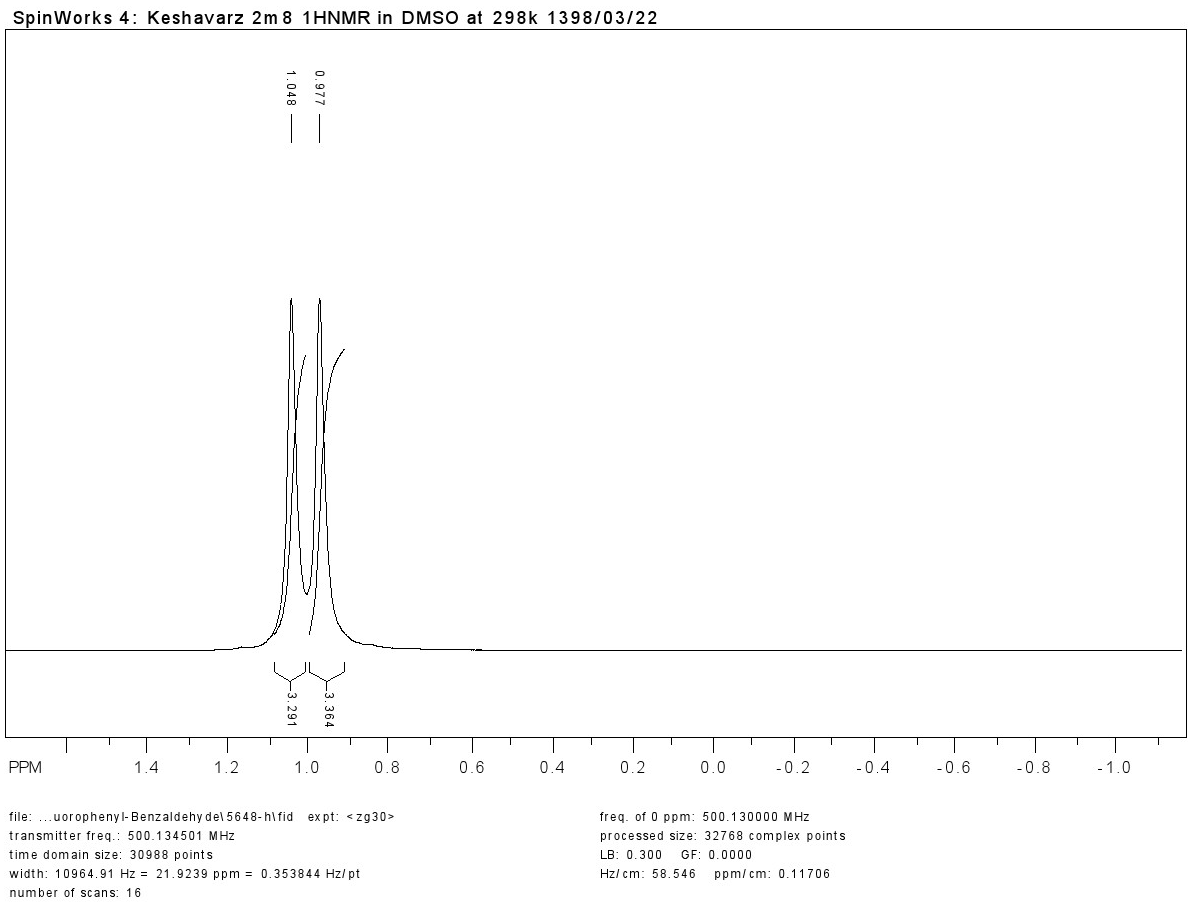


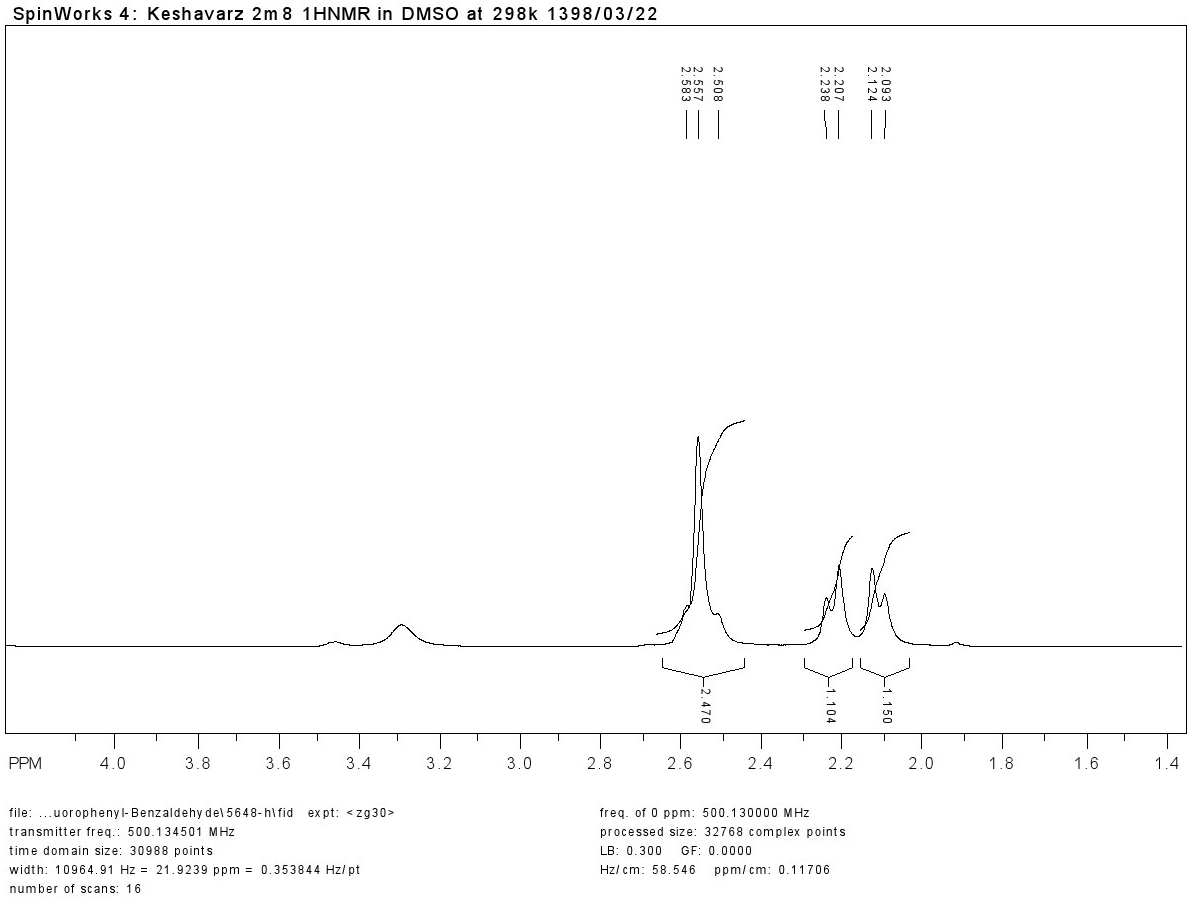


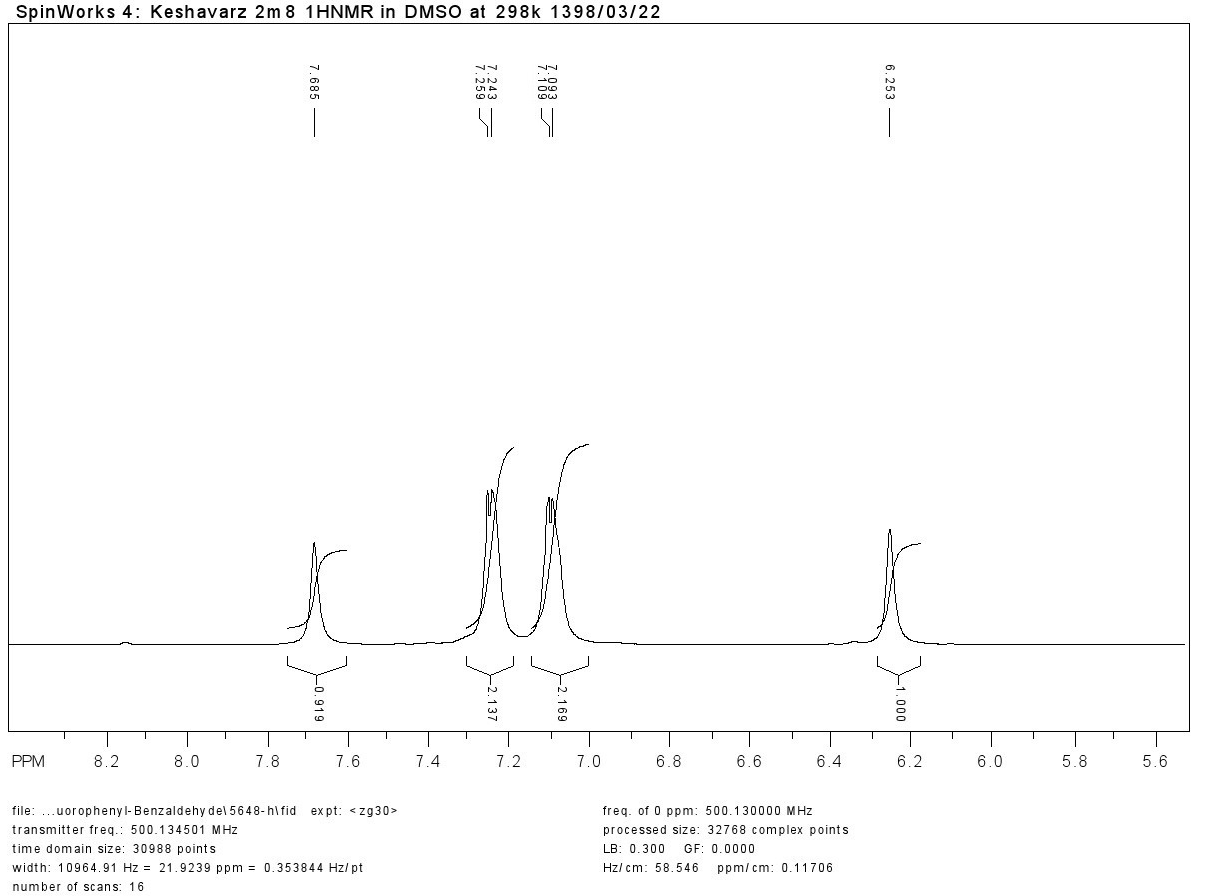


- ^13^C NMR spectrum of (**5j**, 125 MHz, DMSO-*d*_6_)


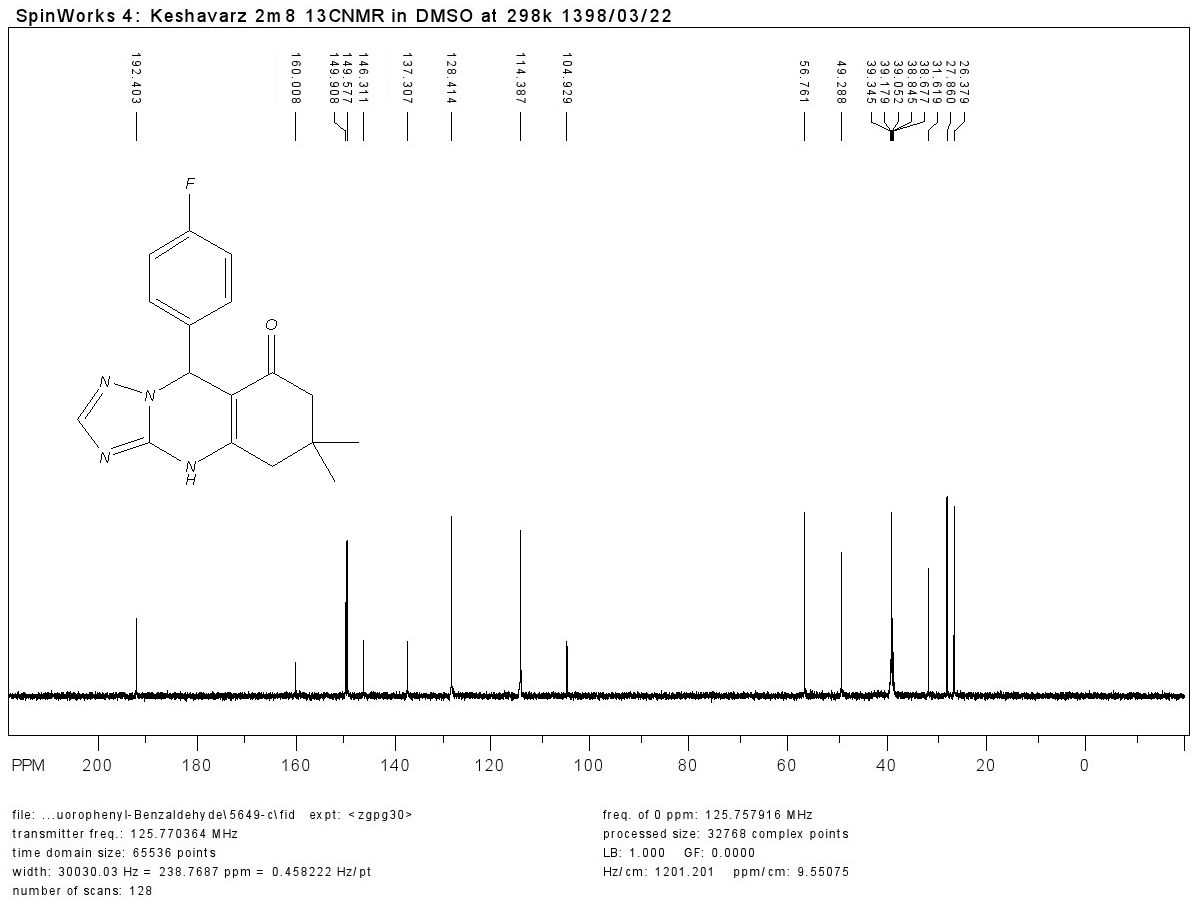

Supplement: Supplementary file 1 — Supplementary Information. [file 41598_2021_91463_MOESM1_ESM.docx]
